# Physiological Network Is Disrupted in Severe COVID-19

**DOI:** 10.3389/fphys.2022.848172

**Published:** 2022-03-10

**Authors:** Antonio Barajas-Martínez, Roopa Mehta, Elizabeth Ibarra-Coronado, Ruben Fossion, Vania J. Martínez Garcés, Monserrat Ramírez Arellano, Ibar A. González Alvarez, Yamilet Viana Moncada Bautista, Omar Y. Bello-Chavolla, Natalia Ramírez Pedraza, Bethsabel Rodríguez Encinas, Carolina Isabel Pérez Carrión, María Isabel Jasso Ávila, Jorge Carlos Valladares-García, Pablo Esteban Vanegas-Cedillo, Diana Hernández Juárez, Arsenio Vargas-Vázquez, Neftali Eduardo Antonio-Villa, Paloma Almeda-Valdes, Osbaldo Resendis-Antonio, Marcia Hiriart, Alejandro Frank, Carlos A. Aguilar-Salinas, Ana Leonor Rivera

**Affiliations:** ^1^Centro de Ciencias de la Complejidad, Universidad Nacional Autónoma de México, Ciudad de México, Mexico; ^2^Facultad de Medicina, Universidad Nacional Autónoma de México, Ciudad de México, Mexico; ^3^Programa de Doctorado en Ciencias Biomédicas, Universidad Nacional Autónoma de México, Ciudad de México, Mexico; ^4^Unidad de Investigación de Enfermedades Metabólicas, Instituto Nacional de Ciencias Médicas y Nutrición “Salvador Zubirán”, Ciudad de México, Mexico; ^5^Instituto de Ciencias Nucleares, Universidad Nacional Autónoma de México, Ciudad de México, Mexico; ^6^Plan of Combined Studies in Medicine (PECEM-MD/PhD), Facultad de Medicina, Universidad Nacional Autónoma de México, Ciudad de México, Mexico; ^7^División de Investigación, Instituto Nacional de Geriatría, Ciudad de México, Mexico; ^8^Departamento de Radiología, Instituto Nacional de Ciencias Médicas y Nutrición “Salvador Zubirán”, Ciudad de México, Mexico; ^9^Departamento de Endocrinología y Metabolismo, Instituto Nacional de Ciencias Médicas y Nutrición “Salvador Zubirán”, Ciudad de México, Mexico; ^10^Instituto Nacional de Medicina Genómica & Coordinación de la Investigación Científica-Red de Apoyo a la Investigación, UNAM, Ciudad de México, Mexico; ^11^Instituto de Fisiología Celular, Universidad Nacional Autónoma de México, Ciudad de México, Mexico; ^12^El Colegio Nacional, Mexico City, Mexico

**Keywords:** homeostasis, network physiology, severe COVID-19, pro-inflammatory state, sex differences

## Abstract

The human body is a complex system maintained in homeostasis thanks to the interactions between multiple physiological regulation systems. When faced with physical or biological perturbations, this system must react by keeping a balance between adaptability and robustness. The SARS-COV-2 virus infection poses an immune system challenge that tests the organism’s homeostatic response. Notably, the elderly and men are particularly vulnerable to severe disease, poor outcomes, and death. Mexico seems to have more infected young men than anywhere else. The goal of this study is to determine the differences in the relationships that link physiological variables that characterize the elderly and men, and those that characterize fatal outcomes in young men. To accomplish this, we examined a database of patients with moderate to severe COVID-19 (471 men and 277 women) registered at the “Instituto Nacional de Ciencias Médicas y Nutrición Salvador Zubirán” in March 2020. The sample was stratified by outcome, age, and sex. Physiological networks were built using 67 physiological variables (vital signs, anthropometric, hematic, biochemical, and tomographic variables) recorded upon hospital admission. Individual variables and system behavior were examined by descriptive statistics, differences between groups, principal component analysis, and network analysis. We show how topological network properties, particularly clustering coefficient, become disrupted in disease. Finally, anthropometric, metabolic, inflammatory, and pulmonary cluster interaction characterize the deceased young male group.

## Introduction

Currently, the world is undergoing a global pandemic of coronavirus infection with SARS-CoV-2 resulting in COVID-19 (Novelli et al., 2021). This infection presents mainly as a severe acute respiratory syndrome that has affected millions of people. Since the beginning of the pandemic, the elderly and men were identified as particularly vulnerable to severe disease, poor outcomes, and death (Carethers, 2021). Although several vaccines have been developed for the prevention of this disease, and great efforts for worldwide vaccination are being made (Case et al., 2021), this disease has the potential to become endemic (Phillips, 2021). A shift in incidence toward younger groups of age, driven by several complex dynamics, has been identified (Malmgren et al., 2021; Monod et al., 2021). Country-specific patterns in these shifts caused by socioeconomic factors provide detail in the changing landscape of this disease (Leong et al., 2021). In Mexico, there appear to be more diseased young men than anywhere else, this is likely due to an underlying epidemic of non-communicable diseases, such as obesity and diabetes (Monterrubio-Flores et al., 2021).

The human body is a complex system maintained in homeostasis as a result of the interactions between multiple physiological regulation systems (the cardiovascular system balances needs like thermoregulation, with and chemo- and baro-regulation, heart rate, blood pressure and vascular tone being the result of the summation of these multiple and constant inputs). When faced with physical or biological perturbations, this system must react by keeping a balance between adaptability and robustness (Fossion et al., 2020). Furthermore, homeostasis is an emergent phenomenon resulting from numerous relationships embedded within the physiological network, the global set of interactions that take place inside the human body (Goldstein, 2019). The SARS-COV-2 virus infection poses an immune system challenge that tests the organism’s homeostatic response (Sieck, 2020). This challenge is not limited to respiratory tract problems. Besides the lungs, several tissues are potential targets to SARS-CoV-2 due to their ACE2 expression (Zou et al., 2020). Particularly, protein expression has been confirmed for endothelial cells in the vasculature of several organs and intestinal enterocytes (Hamming et al., 2004). Autopsy series show that while COVID-19 is conceptualized as a primarily respiratory illness, widespread effects in the body are present (Bryce et al., 2021). Indeed, extra respiratory manifestations are common during and after COVID-19 (Lai et al., 2020; Lopez-Leon et al., 2021).

Consequently, viral-host interaction may be understood as a mix or transition between two dynamics, localized physiological host response and disseminated pathogenic host response (Bohn et al., 2020). Local pulmonary effects propagate through several mechanisms from the infected lungs to the rest of the body, mainly by immune and endothelial cell signaling that generates systemic responses (Teuwen et al., 2020; Gustine and Jones, 2021). The challenge arises in how to characterize the “aberrant regulation” present in severe disease with fatal outcome. The goal of this study is to identify different physiological system states and determine the differences in the relationships that link physiological variables that characterize the traditionally vulnerable populations of elderly and men and those that drive fatal outcomes in young men. The topology of the physiological interactions has immediate functional and dynamic implications that are inaccessible through reductionist approaches and lower-order analysis (Jansson, 2020).

## Materials and Methods

This study included consecutive patients evaluated at the Instituto Nacional de Ciencias Médicas y Nutrición Salvador Zubirán (INCMNSZ), a COVID-19 reference center in Mexico City between 17 March and 31 May 2020. Subjects were initially assessed at triage and required either ambulatory or in-hospital care for COVID-19, confirmed with computerized tomography (CT) and/or *via* RT-qPCR test from nasopharyngeal swabs. All patients had moderate to severe disease as defined by National Institute of Health criteria (National Institutes of Health, 2021). Moderate illness was defined as evidence of lower respiratory disease during clinical assessment or imaging and who have saturation of oxygen (SpO2) ≥ 94% on air, and severe illness as SpO2 < 94% on air, a ratio of arterial partial pressure of oxygen to fraction of inspired oxygen (PaO2/FiO2) <300 mm Hg, respiratory frequency > 30 breaths/min, or lung infiltrates > 50%. In the database, only eight women had moderate disease, but required hospitalization, all other patients had severe disease. All patients underwent a chest CT, and a radiologist determined the degree of pulmonary parenchymal disease and assessed epicardial fat thickness. In addition, a medical history, anthropometric measurements, and laboratory tests were obtained. The present study is a secondary analysis of an existing database that was constructed in the frame of a clinical trial realized at the Instituto Nacional de Ciencias Médicas y Nutrición Salvador Zubirán (INCMNSZ). All study participants gave their written consent that their anonymized data may be used for that clinical trial and other subsequent research protocols that are submitted to and approved by the research and ethics committee of INCMNSZ. The present study was approved by the research and ethics committee (Ref. 3,383) and informed consent was waived due to its nature of being a secondary analysis.

Measurements in this study were taken immediately upon arrival to the hospital, prior to the establishment of medical treatment. This dataset contains 53 physiological variables including vital signs, oxygenation, anthropometry, biochemical and cytological blood parameters, and CT scan evaluation, derived variables employed in the clinical setting, and two variables of hospitalization and ventilatory support time (Supplementary Tables 1 and 2).

This sample was stratified according to sex, age, and disease outcome. Men and women were divided into two age groups for analysis: old (over 60 years old) and young (under 60 years old). Hospitalized patients were followed until they recovered and were medically discharged or died. The database contained 748 patients in total. From the recovered group, 25 patients were transferred to other hospitals prior to full hospital discharge, seven of them were transferred to medical facilities dedicated exclusively to patient recovery. Patients who were still receiving treatment at the end of the study period (*n* = 48) were excluded from the analysis.

**Figure 1:**
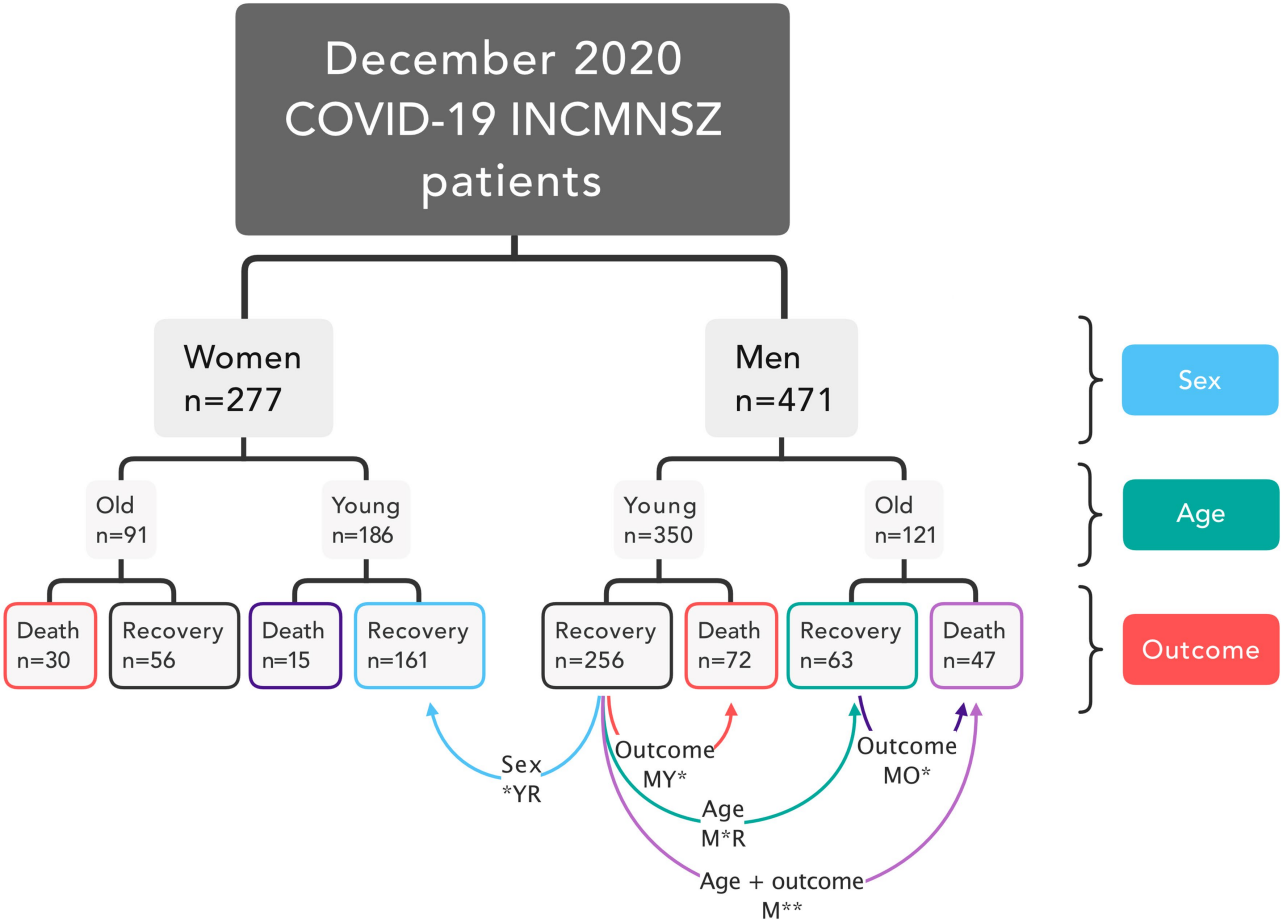
Study design. Patients in the database were classified based on their sex, age, and disease outcome. The network analysis included five groups. Four comparisons were made for groups that differed in sex, outcome, age, and both age and outcome.

This resulted in eight groups of patients. From these groups, only four had more than 60 patients each: recovered young females (FYR), recovered young men (MYR), deceased young men (MYD), recovered old men (MOR), and an additional group comprising deceased old men (MOD) was included. Comparison between these groups allowed us to check for differences likely due to outcome in young males, age in males, and sex in young and recovered, outcome in old males and both age and outcome in males (Figure 1).

### Model Rationale

The *milieu intérieur* (internal media) comprises several-regulated physiological variables necessary for cellular function, being kept within boundaries (setpoints). To keep these regulated variables outside their thermodynamic equilibrium and within healthy limits, entropy must be transferred elsewhere in the system through effector variables (Fossion et al., 2021). In this model, if a variable is kept ideally within its setpoint, no correlation between the regulated variables and the effector variables is found because their perturbations are quenched successfully. Gaussian distributions would suggest a successful homeostatic regulation that “erases” the memory of past perturbations.

However, when an external or internal perturbation “pushes” a regulated variable outside its setpoint, a negative feedback loop is activated to counteract the perturbation (Fossion et al., 2018). For a regulated variable, these perturbations may occur in both directions. In contrast, an effector variable has a preferred direction of action in which it responds. In this loop, the related effector variable changes in function of the deviation of the regulated variable from the setpoint but exerting a balancing effect on the system. Effector variables need time to counteract the perturbations, sustaining their change in one direction for a length of time. As both variables are entrained, their values correlate, showing their shared fluctuation.

Over time, the counteracting of the effector variables absorbs the entropy from the regulated variables. This results in regulated variables keeping Gaussian behavior and effector variables exhibiting long tails (Rivera et al., 2018).

Perturbations cascade from related variable to variable across the system until all alterations of the *milieu intérieur* reach their setpoint. Some regulated variables may be effector variables depending on the direction of this cascade. Multiple simultaneous perturbations may have synergic or counteracting effects as they cascade.

In this model, in a pathological state, the entropy pump becomes less efficient, requiring increasing response of the effector variables to quench the perturbation of the regulated variables. The regulated variable that departs from its Gaussian behavior indicating wear in the system. This results in increasing correlation between physiological variables, producing network topology change characterized by increased transitivity and clustering coefficient (Barajas-Martínez et al., 2020). This cumulative damage hinders the response to future perturbations.

The disease state is established once a regulated variable loses its constraints. This results in a decrease of the correlation between physiological variables, decreasing clustering coefficient, and increasing modularity (Barajas-Martínez et al., 2020). In an infectious disease, a physiological response is elicited by immune recognition of non-self-elements present in the microorganism. Both this response and the damage elicited by the microorganism change the way physiological variables interact. This will result in topological changes that show how physiology is affected by disease.

In the present contribution, unless explicitly stated, the same mathematical methodology and parameters have been used as in our previous publications on physiological networks in this journal (Barajas-Martínez et al., 2020, 2021a,b). All calculations have been carried out in R, in particular using the packages igraph, which includes the Louvain and InfoMAP algorithms implementation, and ForceAtlas2 for the topological clustering (Barajas-Martínez et al., 2020, 2021a,b).

### Normalization Procedure

Physiological variables are measured in several different units to quantify distances, weights, substance concentrations, cellular amounts, etc. These differences make comparisons between variables difficult. We use a normalization procedure based on the normal ranges and criteria reported in clinical guidelines and medical references (Barajas-Martínez et al., 2021a; Supplementary Table 1 for men and Supplementary Table 2 for women). This allows for immediate identification of values above (>1) or below (<0) the thresholds set on the best evidence present in the literature. If there are no applicable ranges or health guidelines in the literature for certain physiological variables, the range of data values was used for normalization.

### Descriptive Statistics

Firstly, data distribution of physiological variables may be examined. Regulated variables have a setpoint in health, and in consequence, their values will oscillate closely around said mean, resulting in a Gaussian distribution. On the other hand, effector variables will increase their variation coefficient, skewness, and excess kurtosis to account for the disturbances of regulated variables (Fossion et al., 2020). As the capacity of the effector variables to quench these disturbances is reduced, this landscape of distributions changes, allowing for a loss of the Gaussian distribution of the regulated variables, and as effector variables become worn or exhausted, are unable to sustain their uni-directional response and may appear more Gaussian.

Descriptive statistics were computed for each physiological variable. For the full dataset and each of the main groups studied in this study, mean (μ) standard deviation (σ), coefficient of variation (σ/μ), skewness (sk), excess kurtosis (k), deviation from Gaussian behavior (⍺), and number of measurements (n) are provided (Supplementary Table 3; Supplementary Figure 1).


$$\alpha =\sqrt{{\frac{\sigma }{\mu }}^{2}+sk^{2}+k^{2}}$$

Because the majority of the physiological variables did not have a Gaussian distribution, Mann–Whitney U tests were used to compare each group to the MYR group. Fisher’s exact test was performed to evaluate whether there were significant differences in the prevalence of certain conditions between groups. Due to the multiple comparisons performed, values of *p* were adjusted with the Holm-Sidak method.

### Principal Component Analysis

Next, the physiological variables’ mutual dependence was examined. The Spearman correlation coefficient is a non-parametric test that indicates monotonic relationships between variables. The correlation matrix of the whole database was examined by principal component analysis (PCA; Vásquez-Correa and Rodas, 2019). Variables were ordered according to the first principal component followed by optimal leaf order for hierarchical clustering (Bar-Joseph et al., 2001). To further analyze the underlying data structure obtained by PCA, we examined the components selected by Bayesian information criterion (Revelle, 2021).

### Network Analysis

Because each group of interest had a different number of patients, from each group 60 patients were randomly selected to generate correlation matrices. This procedure was repeated 30 times and the correlation matrices were averaged. The Spearman rank order correlation matrix of each group was filtered by *p* < 0.05 and determination coefficient > 1%. Perfect correlations between a variable and itself, and between a variable and its derived variable are discarded. This adjacency matrix is then presented as a network, where it is possible to visualize functional clusters of physiological variables and the paths that perturbations follow across the physiological system, as well as the changes in the interactions between physiological variables and in the structure and organization of the physiological network. Results from 30 networks were averaged to analyze differences between graph level indices and centrality measures.

The full physiological networks are provided in (Supplementary Figures 2–6). In these networks node, color shows the median value of the normalized physiological variable, and the size shows the divergence from normal distribution. These nodes are placed according to a LinLog energy model where distance depends on edge density. If two nodes share the same edges they are superimposed. Nodes were grouped by the Spinglass algorithm, which works by quality metric optimization to find which nodes belong to the same community. Network analysis was performed (Barajas-Martínez et al., 2021b). Laplacian centrality was selected as the measure of radial centrality in the network (Qi et al., 2012) while fractional flow betweenness was chosen to measure medial centrality (Koschützki et al., 2005). Physiological variables may behave differently in different networks, altering the centrality values of nodes within networks. Medial node centralities depend on the walk structure, they are prone to change more easily from network to network (Costenbader and Valente, 2003). Spinglass clustering was selected to detect communities in the networks (Reichardt and Bornholdt, 2006). Clustering was compared by normalized mutual information and variation of information (Meilă, 2003). Graph structure was compared by product–moment correlation, structural correlation, and Weisfeiler-Lehman isomorphism test (Shervashidze et al., 2011). Overall, we can use this methodology to look for differences in the structure of the correlation between physiological variables. This, in turn, reflects how COVID-19 alters physiology.

#### Network Simplification

Physiological networks were constructed from the filtered matrices for females young and recovered, males young and recovered, males old and recovered, males young and deceased, and males old and deceased (Supplementary Figures 2–6). To facilitate visualization and meaningful interpretation of the physiological networks, InfoMAP algorithm was used to obtain the minimal description length of a random walker’s movements on a network (Rosvall and Bergstrom, 2008). The network was simplified by collapsing the nodes of each InfoMAP cluster into the node with greatest eigencentrality creating “supernodes” that encompass several closely connected variables. These simplified networks highlight interactions between distant components in the network.

## Results

### Database Description

Our sample comes from a tertiary care referral hospital, and as such may not reflect current national epidemiological trends. Most patients were men, young, and recovered. In contrast, few young women reached a fatal outcome. Diabetes prevalence was similar between recovered and deceased groups, with the exception of young males where diabetes prevalence was doubled (17% in males young and recovered to 36% in males young and deceased, chi-square = 12.62, df = 1, *p* = 0.0004).

### System-Wide Interactions Are Revealed By PCA

**Figure 2:**
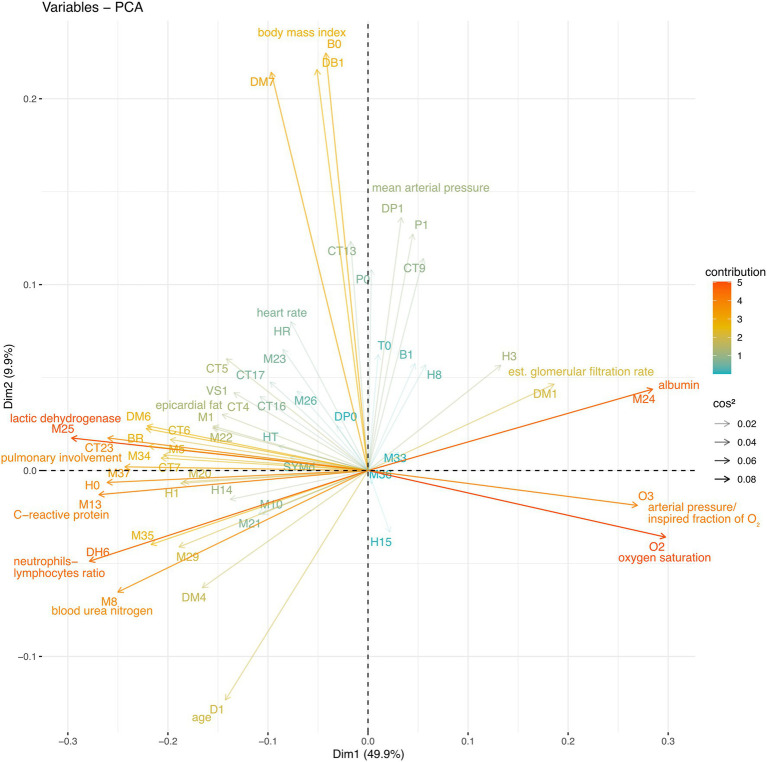
Whole database PCA. The Spearman correlation matrix of the entire database was subjected to PCA. The physiological variable loadings are plotted against the PCA1 and PCA2 projections and colored to indicate their contribution, while the transparency indicates cos^2^.

By avoiding varimax rotation and keeping the orthogonality of the traditional principal components analysis, we were able to isolate “long distance” interactions between physiological variables (Table 1; Figure 2). These interactions showcase first the main physiological problem in the database, COVID-19 pulmonary damage, second the obesity and blood pressure relation, and third the age-related physiological decay.

**Table 1:** Principal components analysis.

	PC1	PC2	PC3	PC4	PC5	PC6	PC7	PC8
Albumin	0.23	0.08	−0.05	−0.12	−0.06	0.11	−0.17	−0.02
Oxygen saturation	0.24	−0.07	−0.05	−0.1	0	0.08	−0.06	−0.08
PaO_2_/FiO_2_	0.22	−0.03	−0.03	−0.04	−0.05	0.02	0.05	−0.04
Estimated glomerular filtration rate	0.15	0.09	−0.13	0.2	0.08	−0.15	0.31	−0.15
Lymphocytes	0.11	0.1	−0.01	0.05	0.02	0.03	−0.13	−0.25
Thoracic subcutaneous a. t.	0.05	0.21	0.27	0.18	−0.02	−0.05	0.05	−0.1
TyG-BMI index	−0.08	0.39	0.2	0.05	−0.05	0.12	−0.03	−0.22
Body mass index	−0.04	0.4	0.17	0.02	−0.12	−0.01	0	−0.21
Weight	−0.03	0.41	0.07	−0.05	−0.21	−0.05	−0.05	−0.1
Hepatic steatosis	−0.01	0.23	−0.08	0.09	−0.07	0.13	0.07	0.06
Heart rate	−0.06	0.15	−0.2	0.11	0.11	0.03	−0.18	0.19
Axillary temperature	0.01	0.11	−0.09	0	0	−0.04	−0.14	0.15
Prothrombin	0	−0.01	−0.14	−0.08	−0.12	−0.15	−0.22	0.03
Vitamin D	0.03	0.02	−0.14	−0.08	−0.07	−0.03	−0.13	−0.02
Height	0.04	0.11	−0.07	−0.03	−0.19	−0.1	−0.05	0.18
Hemoglobin	0.05	0.1	−0.02	−0.11	−0.09	−0.02	−0.13	−0.08
Diastolic pressure	0.04	0.23	−0.09	−0.24	0.27	−0.03	−0.03	0.09
Mean arterial pressure	0.03	0.25	−0.03	−0.27	0.39	−0.07	0.01	0.15
Systolic pressure	0	0.2	0.07	−0.22	0.44	−0.09	0.05	0.19
Pulse pressure	−0.03	0.04	0.14	−0.07	0.29	−0.09	0.09	0.15
Cytopenia	0.02	−0.06	0.13	−0.04	−0.08	0.13	0.03	0.3
Serum creatinine	−0.09	−0.04	0.22	−0.01	−0.04	0.18	−0.33	0.17
Consolidation	−0.09	0.07	−0.07	0.34	−0.03	−0.22	0.05	0.22
Grounded glass opacity	−0.08	0.09	−0.1	0.34	−0.04	−0.22	0.05	0.22
Ventilatory support time	−0.11	0.08	−0.07	0.1	−0.06	0.05	−0.15	0.11
Hospitalization time	−0.07	0.02	−0.11	0.07	−0.05	0.08	−0.15	0.14
Platelets	−0.11	−0.03	−0.17	0.12	0.19	−0.06	0.1	−0.21
Symptomatic time	−0.06	0.01	−0.11	−0.1	0.01	−0.1	0.14	−0.14
Indirect bilirubin	−0.09	−0.04	−0.09	−0.28	−0.1	−0.15	0.02	−0.17
Direct bilirubin	−0.15	−0.01	−0.11	−0.25	−0.15	−0.11	0.05	−0.12
Epicardial fat	−0.12	0.06	−0.1	−0.16	−0.14	−0.05	0	0.01
Creatinine kinase	−0.06	0.08	−0.03	−0.11	−0.17	0.16	−0.04	0.17
Alanine transaminase	−0.07	0.12	−0.19	−0.1	−0.1	0.22	0.35	0.05
Aspartate aminotransferase	−0.13	0.04	−0.12	−0.12	−0.17	0.23	0.34	0.13
Ferritin	−0.18	0.02	−0.18	−0.2	−0.1	0.12	0.09	0.02
Lactic dehydrogenase	−0.24	0.03	−0.09	−0.02	−0.09	0.12	0.11	0.04
Respiratory rate	−0.18	0.04	−0.04	0.11	0.07	−0.03	0	0.05
Pulmonary involvement	−0.21	0.03	−0.06	0.1	−0.03	−0.14	0.02	−0.03
Fibrinogen	−0.2	0	−0.2	0.04	0.13	−0.1	0	−0.12
C-reactive protein	−0.22	−0.02	−0.09	−0.01	0.07	−0.07	−0.07	−0.04
Neutrophils/lymphocytes	−0.23	−0.09	−0.12	−0.04	0.02	−0.06	−0.11	0.08
Leukocytes	−0.21	−0.01	−0.15	0.04	0.09	−0.05	−0.2	−0.16
Total neutrophils	−0.15	−0.01	−0.15	0.03	0.07	−0.03	−0.29	−0.18
Triglycerides	−0.13	0.04	0	0.13	0.13	0.38	0.04	−0.06
TyG index	−0.18	0.04	0.06	0.08	0.16	0.36	−0.02	−0.08
Glucose	−0.17	0.02	0.09	−0.02	0.11	0.14	−0.08	−0.03
Blood urea nitrogen	−0.21	−0.12	0.16	−0.08	0.02	0.14	−0.18	−0.06
BUN to creatinine ratio	−0.14	−0.12	0.15	0.02	0.11	−0.01	0.06	−0.27
D-dimer	−0.18	−0.07	0.08	0.02	0	0.02	0.18	0.01
Ultrasensitive troponin	−0.15	−0.08	0.25	0.1	0.04	0.01	0.08	0.11
Age	−0.12	−0.23	0.26	−0.16	0.04	−0.1	0.08	−0.02
Right pulmonary artery	−0.16	0.03	0.21	−0.19	−0.12	−0.23	0.04	0.05
Left pulmonary artery	−0.17	0.01	0.21	−0.12	−0.15	−0.2	0.04	0.05
Pulmonary artery trunk	−0.12	0.11	0.23	0.03	−0.09	−0.21	−0.04	0.06

*Physiological variable contributions to each selected principal component are tabulated according to optimal leaf order*.

In the entire sample, the first principal component accounted for 50% of the variation (Figure 2). PC1 was mostly determined by biomarkers of COVID-19 severity, neutrophils to lymphocytes ratio (DH6), lactic dehydrogenase (M25), C-reactive protein (M13) and pulmonary involvement (CT23), and health biomarkers albumin (M24) oxygen saturation (O2) and arterial oxygen pressure to inspired fraction of oxygen ratio (O3). Favorable and unfavorable variables are placed in opposition along the first principal component (Figure 2; Table 1).

The second principal component accounted for 10% of the variance in the entire sample, with triglycerides to body mass index (DM7), body mass index (DB1), and weight (B0) contributing to PC2. The second principal component also shows shared variance between the anthropometric indicators and blood pressure (Table 1). The third principal component shows the shared variance between age (D1)-related biomarkers, including ultrasensitive troponin, pulmonary artery main trunk diameter, serum creatinine, and thoracic subcutaneous adipose tissue.

The fifth and sixth principal components show well-known physiologically relevant interactions. The fifth principal component showcases distinctively only blood pressure measures, revealing their mechanical relationship. The sixth principal component selects triglycerides, triglycerides to glucose ratio and hepatic damage enzymes alanine transferase, and aspartate aminotransferase. These variables are indicators of hepatic steatosis.

Abnormal interactions which take place during sepsis are presented in the fourth, seventh, and eight principal components. The fourth principal component shows biomarkers positively related as ferritin, estimated glomerular filtration rate, and biomarkers negatively related as blood pressure and direct and indirect bilirubin. The seventh principal component isolates serum creatinine, estimated glomerular filtration rate, total neutrophils, alanine transferase and aspartate aminotransferase, prothrombin, and D-dimer. Finally, the eight principal component reveals the interaction between cytopenia, BUN to creatinine ratio, lymphocytes with consolidation, and ground-glass image.

### COVID-19 Severity Biomarkers

**Figure 3:**
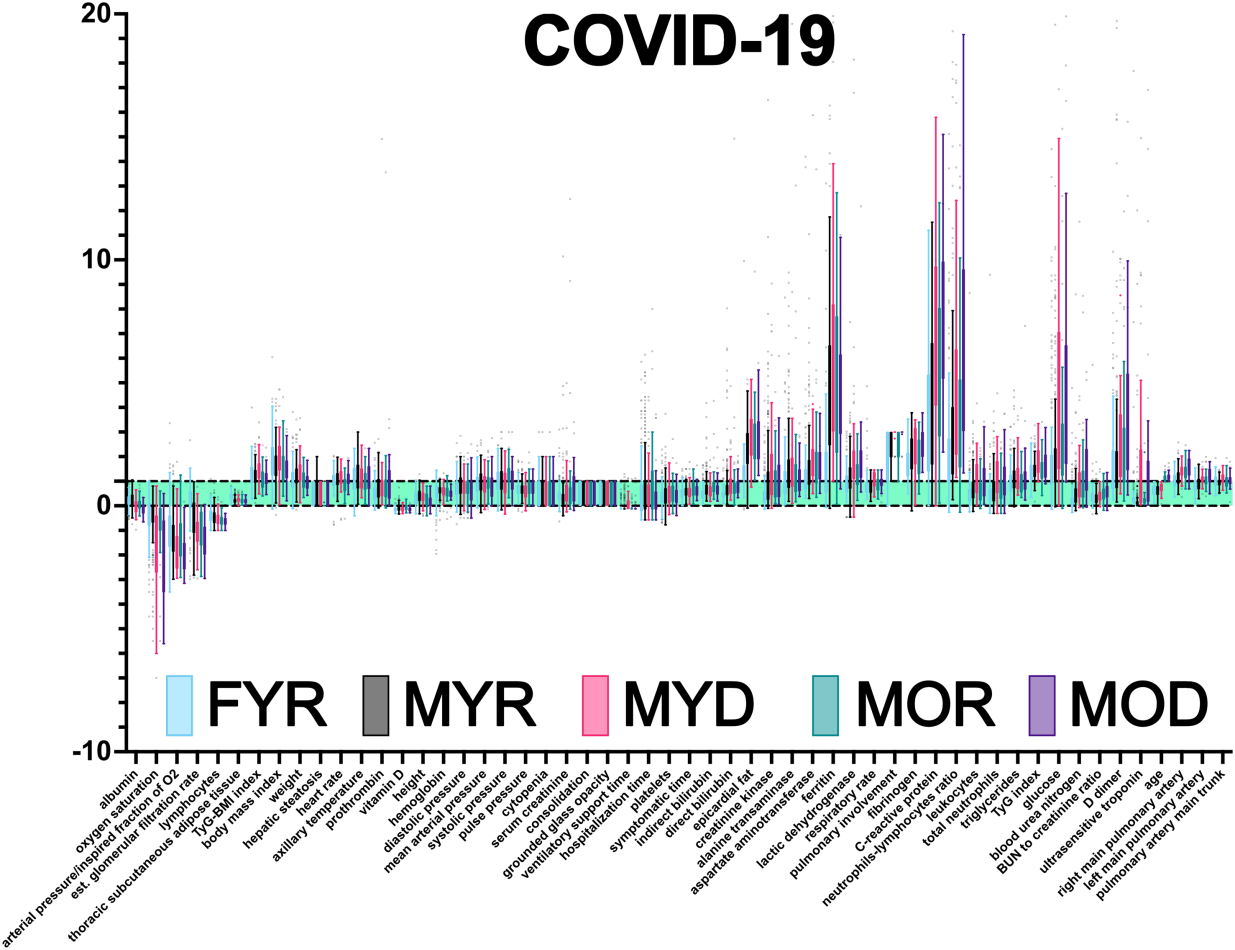
Database normalization. Using health intervals reported in the literature, the raw data were normalized (Supplementary Tables 1 and 2). Values are presented as Tukey’s box-plots. Values less than 0 are below the acceptable minimum. Values greater than one indicate that the individual has exceeded the highest threshold. Values between 0 and 1 (green shadowing area) are considered healthy.

An advantage of our normalization procedure is the immediate identification of physiological variables outside the healthy range (Figure 3). Oxygen saturation, the ratio of O2 arterial partial pressure to O2 inspired fraction (also known as Horowitz index), vitamin D, estimated glomerular filtration rate, total lymphocyte count, and lymphocyte percentage all had lower values. The majority of the other physiological variables had increased values, except height, serum creatinine, direct and indirect bilirubin, albumin, BUN to creatinine ratio, hemoglobin, platelets, and epicardial fat. Three physiological variables were found to be several times outside the healthy range, C-reactive protein, ferritin, and neutrophils to lymphocytes ratio.

Multiple comparisons were performed to assess whether a difference in sex, disease outcome, age, or outcome and age affects a physiological variable (Table 2; Figure 3). These comparisons were performed in the normalized values to control for inherent differences due to sex and age that have been described for some physiological variables. For instance, a difference between MYR and FYR values in a physiological variable that had thresholds adjusted by sex indicates that the alteration in values goes beyond the correction. This means that a group is being explicitly affected by sex. Because differences are sought after normalizing, they should not reflect inherent sex differences but differential affection by disease. Some physiological variables are sensitive to only one stratifying characteristic, while others are affected by all.

**Table 2:** Physiological variables differences between groups.

Groups compared	Summary	FYR	MYR	MYD	MOR	MOD
Albumin	bcd	3.8 ± 0.4	3.8 ± 0.5	3.4 ± 0.4^b^	3.6 ± 0.4^c^	3.3 ± 0.4^d^
Oxygen saturation	bde	85.5 ± 8.8	84.7 ± 9.8	72.2 ± 18.3^b^	83.4 ± 10.6	68.2 ± 16.7^d,e^
PaO_2_/FiO_2_	bde	256.4 ± 104.4	226.4 ± 88.1	169.6 ± 94.9^b^	223.1 ± 91.2	151.4 ± 85.6^d,e^
Estimated glomerular filtration rate	abcd	95.4 ± 23.1^a^	72.8 ± 22.9	59.5 ± 24.9^b^	57.3 ± 22.1^c^	48.6 ± 22.4^d^
Lymphocytes	d	956.6 ± 530.2	816 ± 501.3	700.9 ± 443.3	642.2 ± 361.5	547.6 ± 348.2^d^
Thoracic subcutaneous a. t.	ab	23.2 ± 8.4^a^	14.1 ± 7.9	17.3 ± 9.9^b^	13.9 ± 8.3	13.4 ± 6.9
TyG-BMI index		155.3 ± 34.5	150 ± 28.5	165.4 ± 36.9	141.6 ± 28.2	146.8 ± 26.4
Body mass index		31 ± 6.4	29.9 ± 5	31.9 ± 6	29 ± 5.2	28.6 ± 4.2
Weight	ad	76.9 ± 17^a^	86.5 ± 16.2	90.6 ± 20	80.3 ± 13.1	77.5 ± 11.2^d^
Hepatic steatosis	c	0.4 ± 0.5	0.4 ± 0.5	0.5 ± 0.5	0.1 ± 0.4^c^	0.3 ± 0.4
Heart rate	ce	101.4 ± 20.1	105.4 ± 15.7	107.3 ± 21.1	92.3 ± 18.8^c^	105.6 ± 16.1^e^
Axillary temperature		37.1 ± 0.8	37.3 ± 0.9	37.2 ± 0.9	36.9 ± 0.7	37.2 ± 0.8
Prothrombin	a	11.6 ± 1.3^a^	11.9 ± 1.2	12.3 ± 4.4	14 ± 13.7	14.5 ± 16.2
Vitamin D	a	20.4 ± 7.1^a^	23.7 ± 9.9	20.8 ± 7.4	25.3 ± 9.4	20.3 ± 6.4
Height	d	1.6 ± 0.1	1.7 ± 0.1	1.7 ± 0.1	1.7 ± 0.1	1.6 ± 0.1^d^
Hemoglobin	d	16.6 ± 23.5	16.1 ± 1.5	16 ± 1.6	15.8 ± 1.9	15.2 ± 1.6^d^
Diastolic pressure		74.8 ± 10.4	77.5 ± 10.3	75.8 ± 10.9	74.9 ± 11.2	73.2 ± 12.6
Mean arterial pressure		90.7 ± 10.5	92.8 ± 10.7	90.6 ± 11.7	92.1 ± 11	88.4 ± 14.6
Systolic pressure		122.5 ± 15.1	123.3 ± 15.1	120.1 ± 17.6	126.4 ± 16.2	121.7 ± 17.4
Pulse pressure		47.7 ± 13.2	45.8 ± 12	44.4 ± 14.6	51.5 ± 15	50 ± 18.2
Cytopenia		0.4 ± 0.6	0.4 ± 0.6	0.3 ± 0.5	0.4 ± 0.7	0.3 ± 0.6
Serum creatinine	abd	1.2 ± 4.9^a^	1.1 ± 1.1	1.6 ± 2.7^b^	1.4 ± 1.4	1.7 ± 2.8^d^
Consolidation	c	0.5 ± 0.5	0.6 ± 0.5	0.5 ± 0.5	0.3 ± 0.5^c^	0.5 ± 0.5
Grounded glass opacity	c	0.5 ± 0.5	0.6 ± 0.5	0.5 ± 0.5	0.3 ± 0.5^c^	0.5 ± 0.5
Ventilatory support time	b	1.7 ± 5	2.6 ± 6.4	4.8 ± 7^b^	1.5 ± 4.8	2.2 ± 6.1
Hospitalization time		7.6 ± 8.4	9.5 ± 9.6	8.5 ± 6.9	10.1 ± 11.3	7.6 ± 6.7
Platelets		231.1 ± 98.2	230.3 ± 92.2	237 ± 82.8	235.7 ± 90.7	224.8 ± 75.1
Symptomatic time		7.7 ± 4.1	8.1 ± 3.6	8.7 ± 4.3	9.4 ± 5.2	8.9 ± 3.8
Indirect bilirubin	a	0.4 ± 0.2^a^	0.5 ± 0.3	0.5 ± 0.2	0.6 ± 0.3	0.5 ± 0.2
Direct bilirubin	a	0.1 ± 0.1^a^	0.3 ± 0.3	0.3 ± 0.2	0.3 ± 0.6	0.2 ± 0.1
Epicardial fat	a	9.9 ± 5.4^a^	9.2 ± 3	10.4 ± 3.4	10.5 ± 4.3	10.7 ± 3.8
Creatinine kinase	a	94.7 ± 92.2^a^	271.2 ± 420.8	337.6 ± 367.2	226.9 ± 193.4	238.5 ± 237.1
Alanine transaminase	a	45.9 ± 87.1^a^	53.3 ± 41.8	78.9 ± 197	48.8 ± 58.9	57.6 ± 123.5
Aspartate aminotransferase	ab	44.8 ± 48.9^a^	53.2 ± 38.9	128.9 ± 413.6^b^	68.4 ± 93	77.8 ± 150.2
Ferritin	a	428 ± 1227^a^	895 ± 626	1,355 ± 1,592	906 ± 703	1,130 ± 2,112
Lactic dehydrogenase	abde	325 ± 129^a^	395 ± 185	632 ± 643^b^	403 ± 140	551 ± 379^d,e^
Respiratory rate	bd	26.6 ± 11.8	26.5 ± 7.6	34.7 ± 11.3^b^	28.3 ± 10.4	31.2 ± 8^d^
Pulmonary involvement	bde	2 ± 0.8	2.2 ± 0.7	2.7 ± 0.6^b^	2.3 ± 0.7	2.9 ± 0.3^d,e^
Fibrinogen	a	594.7 ± 175.1^a^	690.5 ± 196.4	740.3 ± 208	659.4 ± 201.7	779 ± 151
C-reactive protein	bde	11.7 ± 8.5	14.9 ± 14.2	30.4 ± 86.7^b^	16.1 ± 9.7	36.8 ± 94.9^d,e^
Neutrophils/lymphocytes	abd	7.4 ± 7^a^	10.1 ± 10.5	17.1 ± 17.6^b^	12.5 ± 11.3	21.5 ± 22.1^d^
Leukocytes	ab	7.4 ± 5.2^a^	8.7 ± 4.2	11.3 ± 5^b^	8.1 ± 3.8	11 ± 5.6
Total neutrophils	b	5.3 ± 5.3	6 ± 4.3	8.9 ± 5.5^b^	6.2 ± 4.1	8.4 ± 6.4
Triglycerides		157 ± 71.3	168.4 ± 83.7	179.1 ± 76.9	159.8 ± 77.5	174.9 ± 132.7
TyG index	b	4.9 ± 0.3	4.9 ± 0.3	5.2 ± 0.4^b^	5 ± 0.4	5.1 ± 0.4
Glucose	bd	134.3 ± 73.9	136.2 ± 70.8	199 ± 116^b^	158.6 ± 86.9	217.5 ± 145.2^d^
Blood urea nitrogen	abcd	13 ± 8.4^a^	16.4 ± 10.5	24.2 ± 21.3^b^	25.2 ± 20.8^c^	31.3 ± 17.2^d^
BUN to creatinine ratio	cd	17.2 ± 11.7	15.4 ± 6	17.8 ± 6.9	19.6 ± 6.9^c^	22 ± 6.9^d^
D-dimer	bd	1,115 ± 2,881	1,654 ± 7,311	3,524 ± 10818^b^	1,169 ± 1,005	3,325 ± 7071^d^
Ultrasensitive troponin	abcde	7.4 ± 24.4^a^	29.1 ± 248.5	86 ± 203.7^b^	77.1 ± 509.1^c^	93.3 ± 216.4^d,e^
Age	bcd	44.5 ± 9.5	43.5 ± 9.4	48.6 ± 8.2^b^	66.1 ± 5.6^c^	67.9 ± 6.6^d^
Right pulmonary artery	abcd	19.9 ± 2.7^a^	21.2 ± 3.2	23.2 ± 2.6^b^	23.3 ± 3.3^c^	24.9 ± 3.4^d^
Left pulmonary artery	bcd	20 ± 2.6	21 ± 2.8	23.1 ± 2.3^b^	22.8 ± 2.9^c^	24.1 ± 3.1^d^
Pulmonary artery trunk	b	27.2 ± 3.9	28 ± 3.4	30.7 ± 4^b^	28.8 ± 5.2	30.1 ± 4.8

*Difference between MYR-FYR due to sex in young and recovered (a). Difference between MYR-MYR due to outcome in young men (b). Difference between MYR-MOR due to age in men that recovered (c). Difference between MYR-MOD due to age and outcome in men (d). Difference between MOR-MOD due to outcome in old men (e)*.

Few variables were unaffected by any factor or affected equally in the four groups. This was the case for blood pressure variables P0, P1, DP0, and DP1, axillary temperature (T0), body mass index (DB1) and triglycerides to body mass index ratio, triglycerides (M1), triglycerides to body mass index ratio (DM7), platelets (H14), and presence of cytopenia (H15). Both hospitalization time (HT) and days with symptoms before hospitalization (SYMd) were similar for all groups. A few variables were different between young recovered males and females, prothrombin, vitamin D, bilirubin (direct and indirect), epicardial fat, enzymes (creatinine kinase and alanine transaminase), and ferritin. As expected by PCA, variables placed along the first principal component contributed most to total variance and thus provide best discrimination among groups. These variables were albumin, oxygen saturation, Horowitz index (PaO_2_/FiO_2_), estimated glomerular filtration rate, lactic dehydrogenase, pulmonary involvement, blood urea nitrogen, ultrasensitive troponin, and right and left pulmonary artery diameters.

**Figure 4:**
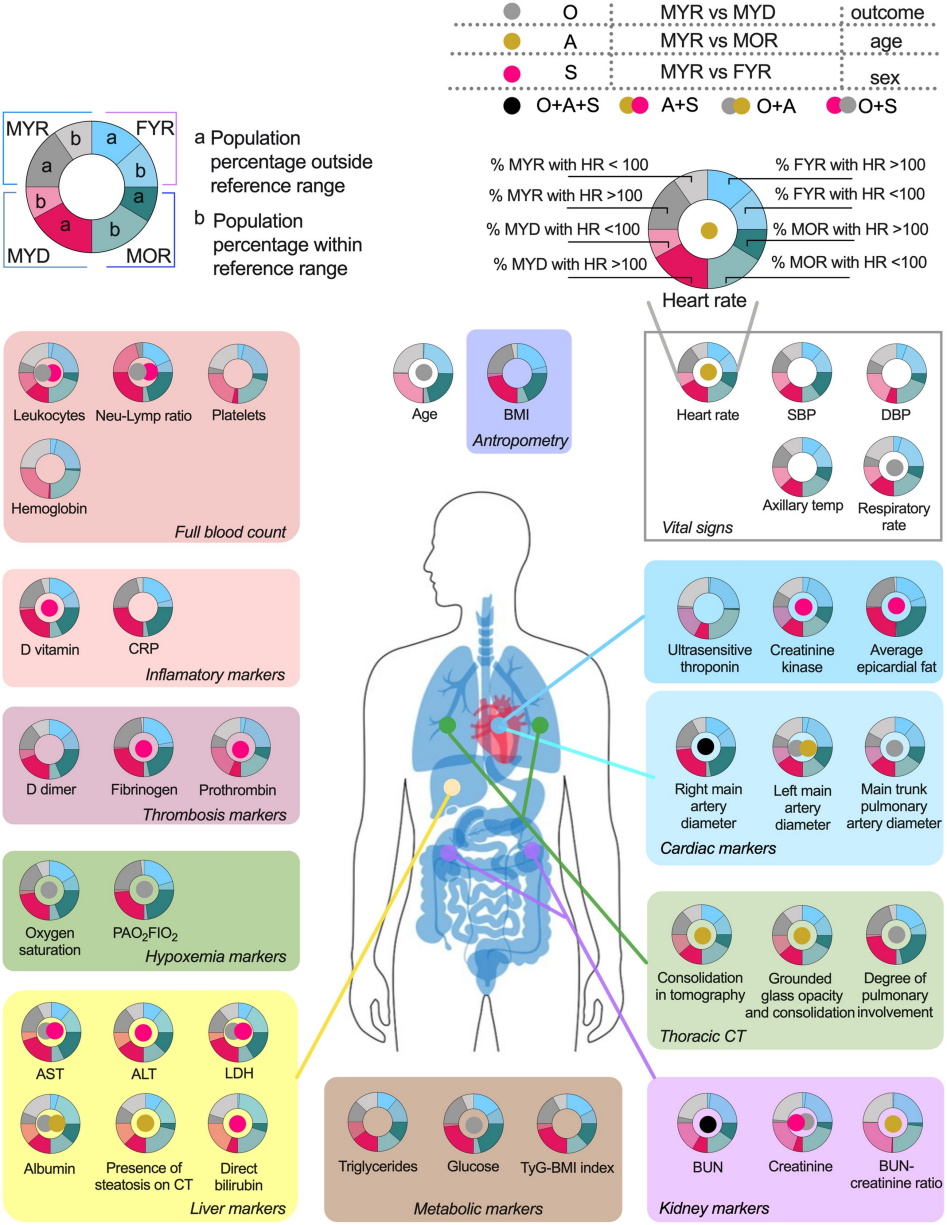
Physiological variables differences and pathologic states prevalence among groups. The variables are grouped according to their usefulness as disorders alterations markers. The pie chart graphics show the behavior of the main study groups, the male young recovered (MYR) group is shown in the light blue pair, the female young recovered (FYR) group in the pink pair, the male old recovered (MOR) group in blue, and the male young deceased (MYD) group in gray. Each pair is made up of a solid shade (a) and a light shade (b). The solid tone (a) represents the population percentage with values outside the reference limits, while the light tone (b) represents the population percentage with values within the reference limits. The circle in the center indicates the statistically significant differences using Mann-Whitney U. Gray circle indicates a significant difference between the MYR and MYD groups (attributed to the outcome), gold circle indicates the difference between the MYR vs MOR groups (attributed to age), red circle indicates the difference between the MYR vs FYR groups (attributed to sex), while the black circle indicates the presence of statistically significant differences between all groups.

COVID-19 affected biomarkers placed across several physiological systems (Figure 4). The proportion of participants within each group that had a physiological variable outside the reference range, and whether a statistical difference in the values was found between groups.

### Correlations Between Physiological Variables Change With COVID-19 Outcome

**Figure 5:**
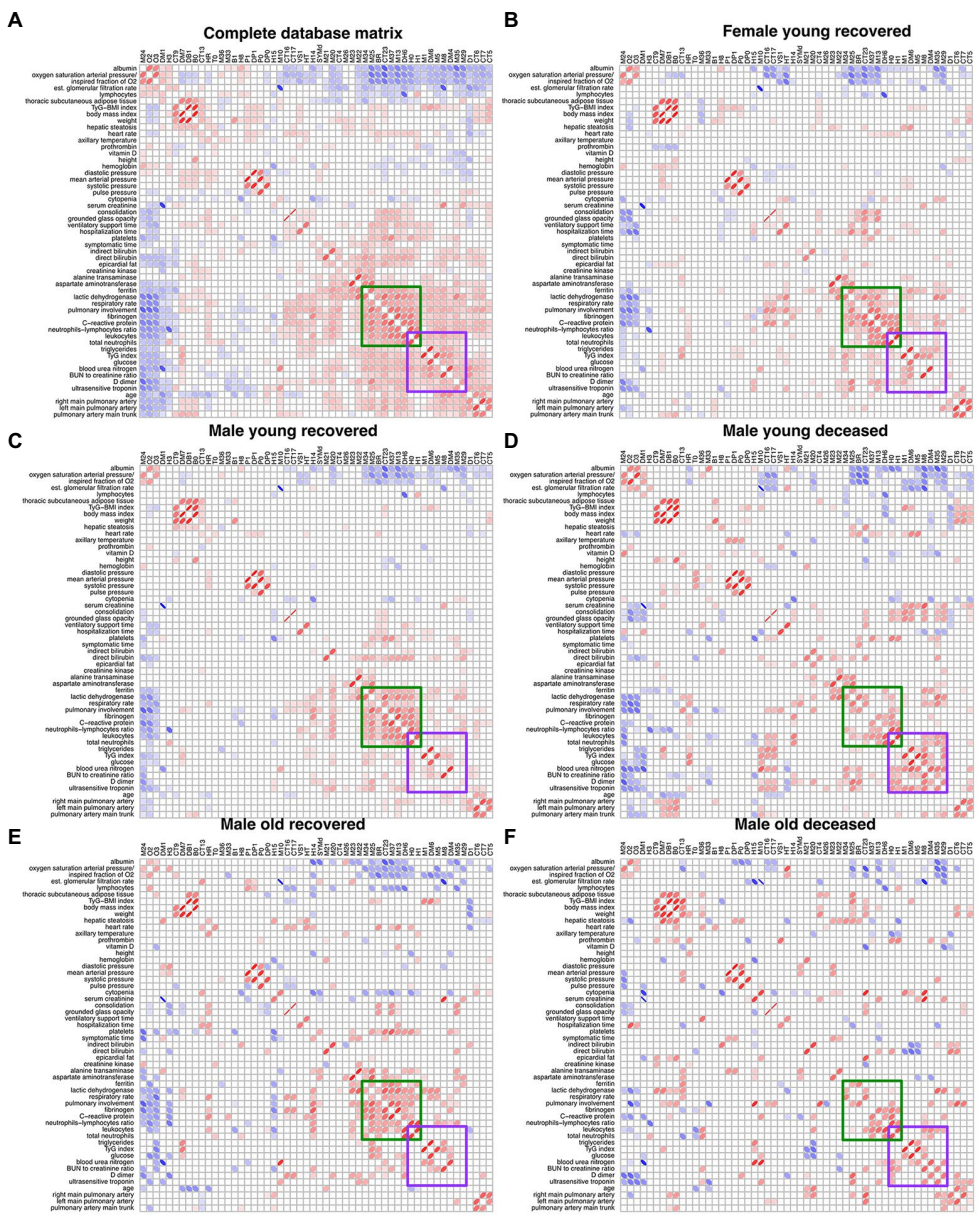
Unfiltered Spearman correlation matrices. **(A)** displays the filtered correlation matrix resulting from the analysis of the entire database. The Spearman correlation matrix for each study group is shown **(B–F)**. Positive correlations are represented by red ellipses, while negative correlations are represented by blue ellipses. The rows and columns are arranged by optimal leaf order. Green square shows a region of correlations present in the networks from recovered groups and the purple square shows a region of correlations present in the deceased groups.

After filtering the Spearman correlation matrices by *p* < 0.05 and determination coefficient above 1%, they were arranged by Optimal Leaf Order to highlight regions where correlations between groups are different (Figure 5). A region of correlations between ferritin, lactic dehydrogenase, respiratory rate, pulmonary involvement, fibrinogen, C-reactive protein, neutrophils to lymphocytes ratio, leukocytes, and neutrophils is present in groups that survived COVID-19 (FYR, MYR, and MOR groups) while is lost for the groups of deceased (Figure 5). In contrast, a region of correlations appears in the non-survival groups between leukocytes, neutrophils, triglycerides, triglycerides to glucose index, glucose, blood urea nitrogen, BUN to creatinine ratio, D_dimer, and ultrasensitive troponin (Figure 5).

### Physiological Network Changes in Severe COVID-19

**Figure 6:**
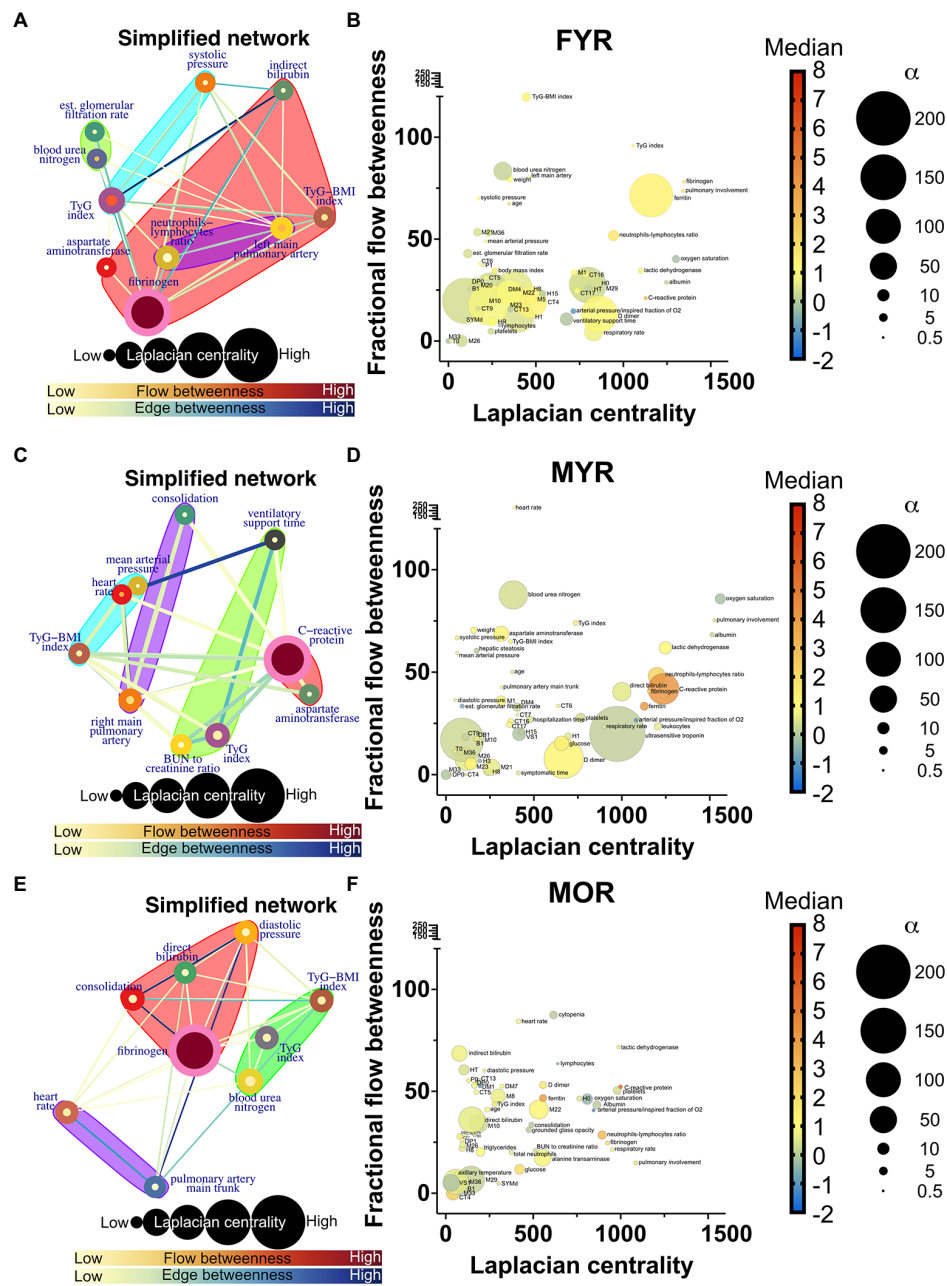
Recovered networks. **(A,C,E)** Represents a simplified network in which supernodes are labeled with the name of the physiological variable with the greatest influence within the InfoMAP community. Edges were kept, resulting in a network with multiple edges. Edge betweenness is represented by the color of the edge. The flow betweenness centrality is represented by node color, and the Laplacian centrality is represented by node size. Clusters are represented by colored shaded areas. **(B,D,F)** The unmerged physiological variables’ Laplacian and fractional flow betweenness centralities were plotted. The color of the nodes represents the normalized median of each variable, whereas the size of the nodes represents the deviation from the normal distribution.

**Figure 7:**
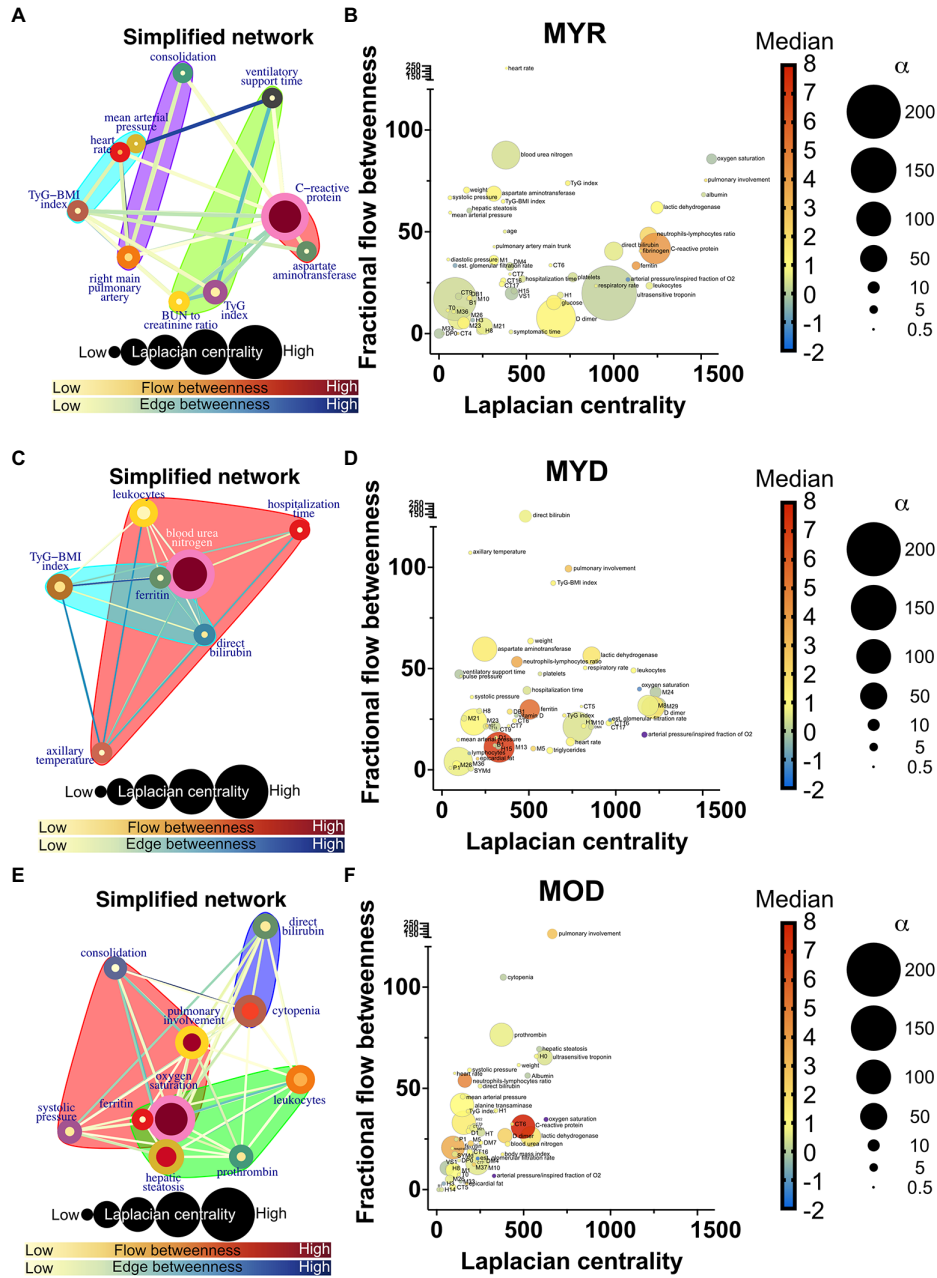
Deceased networks. **(A,C,E)** Represents a simplified network in which supernodes are labeled with the name of the physiological variable with the greatest influence within the InfoMAP community. Edges were kept, resulting in a network with multiple edges. Edge betweenness is represented by the color of the edge. The flow betweenness centrality is represented by node color, and the Laplacian centrality is represented by node size. Clusters are represented by colored shaded areas. **(B,D,F)** The unmerged physiological variables’ Laplacian and fractional flow betweenness centralities were plotted. The color of the nodes represents the normalized median of each variable, whereas the size of the nodes represents the deviation from the normal distribution.

Network simplification procedure reduced the number of meaningful nodes from 53 to 10 or less (Figures 6, 7). In these networks, we found that a main supernode-related pulmonary damage that is surrounded by an ecosystem of other physiological variables. This landscape changes from group to group. Normal physiological modules are preserved in the networks from the recovered groups (FYR, MYR, and MOR). For young and recovered networks (FYR and MYR), the main supernode is connected with aspartate aminotransferase supernode; pulmonary artery diameters supernodes are related with pulmonary damage supernode (neutrophils-lymphocytes ratio for FYR and consolidation for MYR). A metabolic supernode is related to blood pressure supernode (TyG index in FYR or TyG-BMI index in MYR). For the networks with severe COVID-19, these physiological groups are changed. In the networks with decease outcome (MYD and MOD), ferritin supernode interacts closely with the main supernode dominated by blood urea nitrogen (MYD) or oxygen saturation (MOD). For the MYR network, the metabolic supernode TyG-BMI index correlates with ferritin and direct bilirubin supernodes. In the MOD network, prothrombin supernode interacts with leukocytes supernode and hepatic steatosis supernode. Physiological variables that would not interact in more favorable conditions are clustered in the networks with fatal outcome.

The role of each node in the network either as an information flow intermediary or as an influential node was assessed by fractional flow betweenness and Laplacian centrality, respectively, in the right panels. Node color shows the median value of the normalized physiological variable, and the size shows the divergence from normal distribution. For instance, in the MYR networks, oxygen saturation is a variable that is both influential and intermediary, heart rate is only intermediary but not very influential, and the degree of pulmonary involvement is only influential. Physiological networks show that variables that may be well within healthy parameters play critical roles in disease either acting as exchange mediators or drivers within a network, particularly, albumin, bilirubin, and blood urea nitrogen.

**Figure 8:**
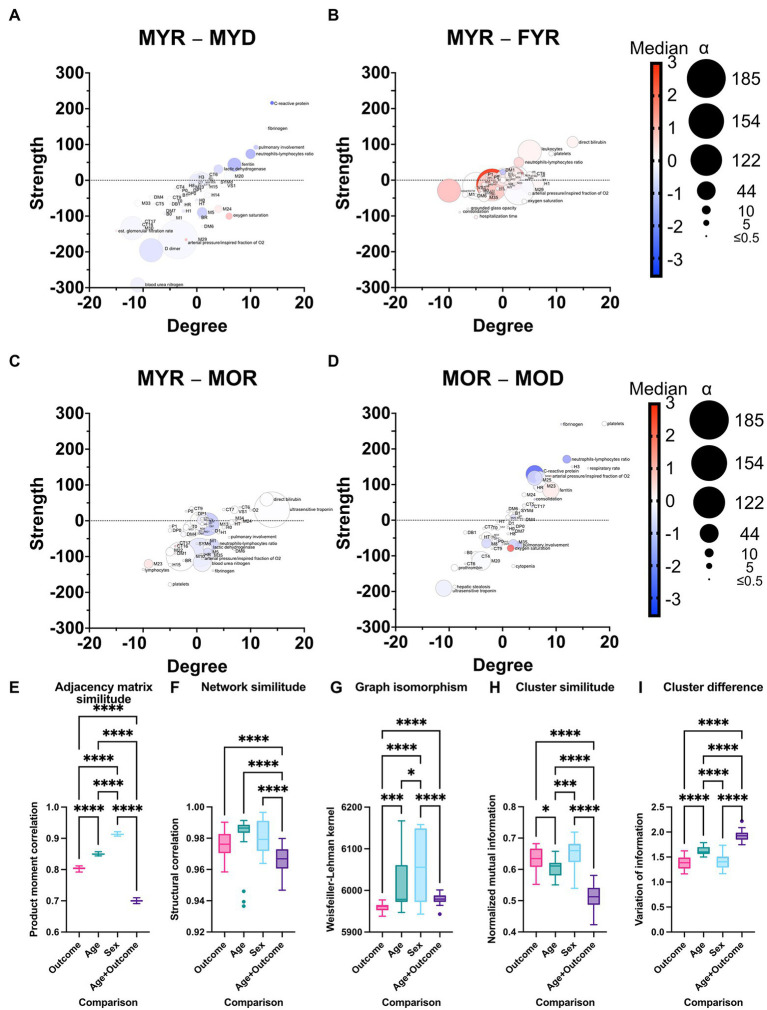
Differences between physiological networks. The difference in node degree and strength against the reference network (MYR) is plotted for comparisons of outcome in young men **(A)**, sex in young recovered patients **(B)**, age in recovered men **(C)**, and outcome in old men **(D)**. The gradient of the normalized median value difference is shown in red to blue. The difference between the distributions is indicated by the size of the nodes. To compare the network structure to the reference, the product–moment correlation **(D)**, structural correlation **(F)**, and Weisfeiler-Lehman isomorphism tests **(G)** were used. Normalized mutual information **(H)** was used to quantify cluster similarity, while information variation was used to quantify cluster difference **(I)**. Statistical difference is shown as for * *p* value <0.05, *** *p* value <0.001, and **** *p* value <0.0001.

Networks were compared by the difference between node degree (number of correlations) and strength (weight of the correlations), and the difference between normalized median value and normality divergence. A positive correlation is observed between the MYR and the MYD strength and degree differences (Figure 8A) and for the MOR and MOD difference (Figure 8D) and between MYR and MOD (Supplementary Figure 7). This positive correlation shows that while some health-related variables lost correlations and strength, the disease-related variables gained correlations and decreased their strength. This is a direct view of the homeostatic process in the organism. In health, physiological processes are kept in balance by several low intensity correlations that fine-tune requirements according to requirements. However, in disease, the pathological process dominates over other physiological regulations; this increases the number of correlations and their strength among disease biomarkers. On the other hand, correlations become lost under pathological conditions. This is seen by the predominance of red nodes in the lower half and blue nodes in the upper half. These strength differences are not observed when comparing networks between the recovered groups (Figures 8B,C).

To further examine where the difference between physiological networks lies, graph structure and clustering were compared. Product–moment correlation shows that vertex labeling controls for underlying structure while structural correlation coefficient shows whether this labeling is exchangeable (Figures 8E,F). Last, the Weisfeiler-Lehman isomorphism test is performed to assess whether different networks are, in fact, the same (Figure 8G). By considering these results, it is possible to see that while isomorphism is quite low, node neighborhoods are different, but both product–moment and structural correlation is high (Supplementary Figure 8). In all cases, FYR network is more similar to MYR network than outcome and age different groups. Despite the differences between networks, the clustering of variables is quite similar (Figures 8H,I). As expected, MOD network was the most dissimilar to the others with respect to all measurements. This is a result of both age and disease outcome changes in the network.

Results of clustering procedure are summarized in Supplementary Figure 9 for each of the 30 iterations of the network that were performed. Clusters were identified by the node with the highest Laplacian centrality, i.e., the core physiological variable around which the other nodes group. Sometimes the same cluster was named differently, but containing the same variables, because two or more nodes were similarly influential. By observing which variables were contained by each cluster, functional roles were suggested for each cluster.

A measure of similitude, the normalized mutual information is higher and the variation of information is lower for females young and recovered. Although changes between network topology are extensive in the MYD group, clustering remains similar, this is not the case in the MOR group. This may well be a reflection of the multiple comorbidities reached by age above 60 that changes the landscape of clusters. Taken together, these comparisons show that young and recovered networks are similar and that age and diseases change both topology and clustering of the networks.

### Topological Changes as Biomarkers of Health and Disease

In a previous study, we examined the differences between young healthy male (MYH) and female (FYH) participants (Barajas-Martínez et al., 2021a). In this work, we hypothesized that FYH networks are more robust while MYH networks are more adaptable. We use these control networks as a reference to interpret the results in our networks of patients with COVID-19, although the set of physiological variables is different for each study.

**Figure 9:**
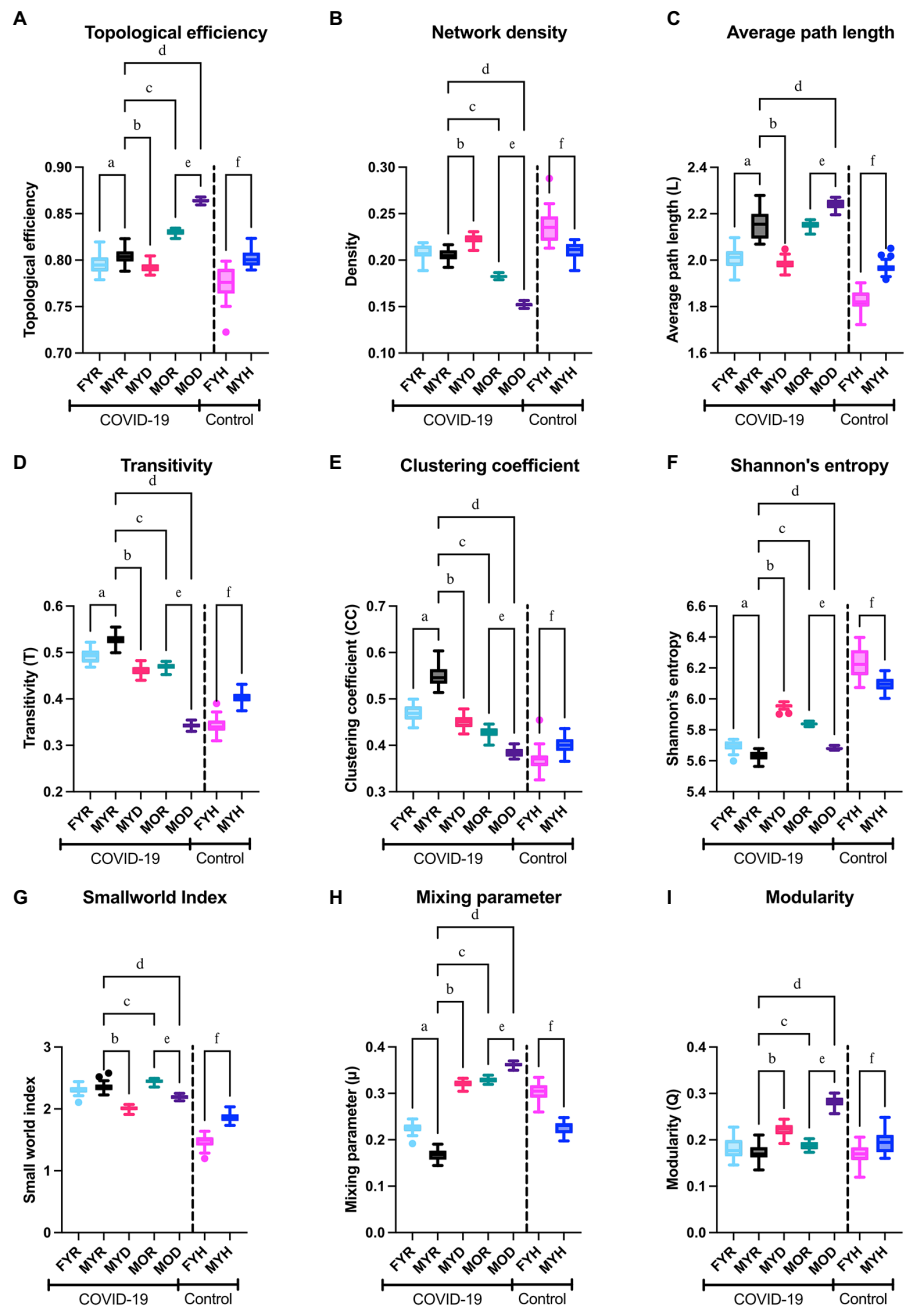
Network topology. The topological efficiency **(A)**, network density **(B)**, average path length **(C)**, transitivity **(D)**, clustering coefficient **(E)**, Shannon’s entropy **(F)**, small world index **(G)**, estimated mixing parameter **(H)**, and modularity **(I)** of the networks from the study groups were compared to those of the reference group (MYR). To demonstrate the behavior of the networks in health conditions, male and female healthy control groups were compared in the columns placed right in each panel. Significant differences between groups MYR and FYR (a), MYD (b), MOR (c), MOD (d), between MOR and MOD (e) and between MYH and FYH (f).

For both healthy participants, sex differences between networks were observed. Female networks are more dense, less efficient, with shorter average path length, higher entropy, and less small world (Figure 9). In the COVID-19 networks, most topological differences between sexes are preserved, except density and small world index, which was to be expected due to COVID-19 disruption of the physiological network.

MYD network is characterized by a decrease in average path length, clustering coefficient, transitivity, and small world index, with an increase in entropy, the mixing parameter, and modularity. Although a simultaneous increase in modularity with decrease in clustering coefficient may seem counterintuitive, a network with a close to zero transitivity can still have a clearly defined community structure (Orman et al., 2012). This speaks of a network that has been “disordered” from the characteristics of homeostatic networks. Furthermore, these changes are increased in the MOD network. When considering outcome differences in old male transitivity, clustering coefficient, small world index, mixing parameter, and modularity remain indicators of fatal outcome for both young and old groups (Figure 9).

### Interactions in Severe COVID-19 With Fatal Outcome

**Figure 10:**
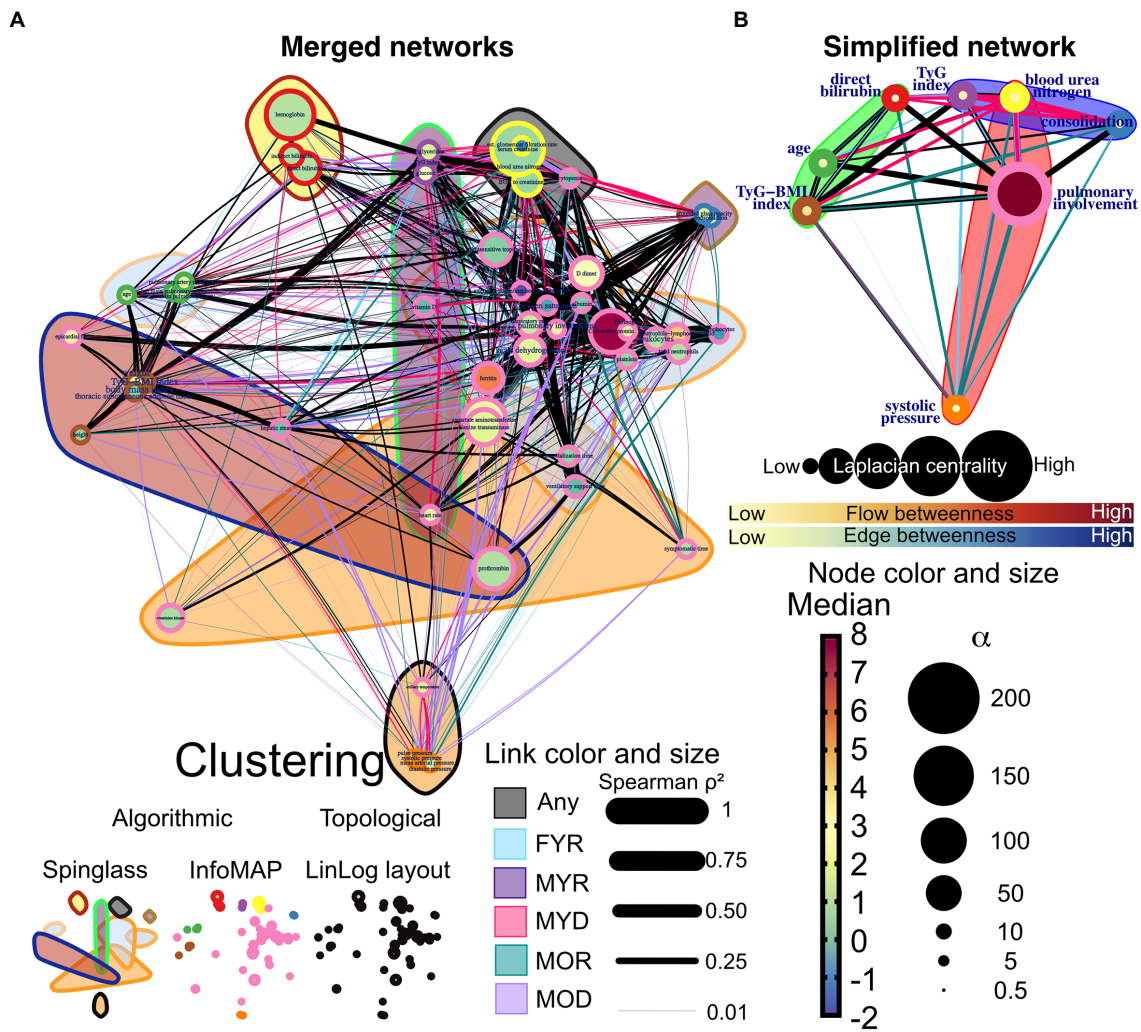
Network differences. **(A)** The networks of the five study groups were merged to determine which edges differed between them. Links that are unique to a study group are highlighted in color, while shared links are black. Each correlation’s strength is indicated by the width of the links. LinLog layout was used to arrange the nodes. The color of the node represents the normalized median value for the whole database, and the size represents the deviation from the normal distribution. The color-shaded areas in this network represent spinglass clusters, while the node border represents the nodes that will be collapsed into a single supernode by InfoMAP clustering. **(B)** Depicts a simplified network in which supernodes are labeled with the name of the physiological variable with the greatest influence within the InfoMAP community. Edges were kept, resulting in a network with multiple edges. The flow betweenness centrality is represented by node color, and the laplacian centrality is represented by node size.

We superimposed networks as a quick visual strategy to identify which relations between physiological variables are exclusive of a group (Figure 10). By visual inspection, MYR and FYR have very few specific edges, with low strength. However, it is notable that the correlations of hepatic steatosis with triglycerides and glucose are present only in FYR network. Age-specific correlations are present between blood pressure variables and pulmonary involvement, pulmonary consolidation, blood urea nitrogen, and bilirubin. Most unique links were present in the MYD and MOD networks. In these networks, the interaction between metabolic variables (triglycerides and glucose) with the pulmonary involvement variables through heart rate is notable for the MYD group. For the MYD group, axillary temperature and blood pressure exhibit a strong correlation. Kidney function biomarkers correlate in the MYD network with metabolic variables, pulmonary involvement cluster and tomographic evidence of ground-glass opacity, and consolidation. Finally, for the MOD group, the blood pressure variables become correlated across the entire network, particularly with hepatic steatosis, hepatic enzymes (aspartate aminotransferase and alanine transaminase), and albumin. From these observations, we can conclude that physiological variables in our analysis are related differently in a way that depends on the outcome of the patient. For the MYD group, the interactions between the anthropometric, metabolic, and pulmonary clusters are apparent and may drive the fatal outcome.

## Discussion

A considerable amount of information regarding possible biomarkers relevant to COVID-19 exists in the literature (Supplementary Table 4). These biomarkers encompass a wide array of physiological systems that include vitamins, cardiovascular, metabolic, tomographic innate, and adaptive immune indicators. Most of these biomarkers are identified by traditional analysis, but network-based strategies (Ahmed, 2020), metabolomics (Hasan et al., 2021), and PCA (Zhang et al., 2021) have been employed successfully, although less commonly. This growing array of available biomarkers lacks an explicative framework on how and why homeostasis becomes altered by COVID-19. Here, we show how the three approaches to data analysis reveal different aspects of data structure collected in a cross-sectional study. Principal components analysis displays relevant components that group salient physiological variables into outcome, age, sex, and adiposity components (Table 1). Traditional analysis shows differences in physiological values between groups (Table 2). Finally, by network representation of physiological interactions, it is possible to identify which variables act as mediators of information flow and which physiological variables are the main drivers in the system (Figures 6, 7). In this work, we show that system-wide changes are present in the physiological network of male young individuals whose disease outcome is fatal. Regulated physiological variables become widely disconnected from their multiple influences in health, and in turn become dependent upon a few physiological variables (Figure 8). In consequence, topological changes take place, indicating that the network has become disordered (Figure 9).

In previous work on healthy young participants, we had shown differences in the connectivity between the networks men and women (Barajas-Martínez et al., 2021a). In this contribution, we provide a detailed explanation of network rigidity, which we believe contributes to the different physiological response to infection, in addition to many other previously described differences between males and females. Regarding network topology, we have found that local clustering coefficients decrease with age, with a simultaneous pathological states increase (Barajas-Martínez et al., 2020), both observations were corroborated in this dataset (Figure 9). A modularity increase accompanied by a clustering coefficient decrease characterizes pathological states of the physiological networks. An advantage of our approach is that the difference we observe occurs at a higher organizational level than, for example, immune differences.

Clustering comparison shows that physiological variables are grouped similarly in all young groups regardless of the topological changes. In contrast, physiological variables are clustered differently in old age (Figure 8). This suggests that the presence of several chronic alterations is required to change the coupling landscape in the organism, such as long-term diabetes and hypertension.

Network analysis allows us to pinpoint the physiological players that are drivers and mediators of disease, and how their importance is different according to disease outcome, age, and sex. An important part of this work was to survey the literature in order to evaluate if our findings were consistent with previous knowledge (Supplementary Table 4). Several biomarkers identified here have also been highlighted in different studies. Particularly, we found that several inflammatory, thrombotic, and hepatic biomarkers change their behavior in COVID-19. In agreement with current knowledge (Supplementary Table 4), well-known cardiovascular biomarkers such as ultrasensitive troponin (Zhu et al., 2021), C-reactive protein, and lactic dehydrogenase were increased in the groups with fatal outcome (Supplementary Table 4). Our work reinforces the role of other less well identified biomarkers such as hepatic features (Ramos-Lopez et al., 2021) and pulmonary artery diameters (Spagnolo et al., 2020). We also provide evidence that several biomarkers could benefit from adjusted thresholds by sex such as ferritin, respectively (Supplementary Table 4). We show that COVID-19 alters the correlation structure of physiological variables and that key mediators and drivers in the network that play a role are not necessarily outside of healthy ranges. This was the case for albumin and blood urea nitrogen. Some specific biomarkers and interactions may be most useful only for certain patient groups. For instance, heart rate in men that are to recover is correlated between physiological clusters, acquiring a high flow betweenness value in the network (Figure 6). This central role is not observable by mean value alone and becomes lost in patients with fatal outcome. This outlines further requirements for identifying biomarkers beyond differences between the mean values of groups (Supplementary Figure 10). Finally, we show that cluster interactions, that show the involvement of different physiological mechanisms, are key features in the group of young male deceased where metabolic, anthropometric, and immune clusters interact (Figure 10).

While Supplementary Table 4 highlights the common observation that the mean values of these biomarkers are found to be altered previously dependent on the severity of the disease, the advantage the data structure approaches allow us to observe how relations cascade between different physiological function modules. Physiological variables increase and decrease their dependency between themselves in the different groups. An integrative view of physiology is useful to follow how apparently unrelated physiological systems are thrown out of balance by systemic effects of COVID-19. After acute disease resolution, several pulmonary and extrapulmonary affections persist, resulting in chronic health loss (Al-Aly et al., 2021). Taken together, it is possible to generate a network-based model of physiological interactions that sustains homeostasis. Our results show that, since the whole physiological network is affected, return to normal homeostatic regulation may be slow, and system-wide alterations are to be expected.

### Limitations

One limitation of our transversal study is that causal connections can be difficult to deduce from cross-sectional analysis. We believe that the literature review of Supplementary Table 4 is paramount for the adequate interpretation of our networks.

It is worth noting that the correlations found in this study are the product of a network that was filtered with a value of *p* threshold. Other approaches, on the other hand, are feasible (Musciotto et al., 2018). The network approach is a powerful tool for the visualization and exploratory analysis multivariate complex datasets, but it has the drawback of only being able to define links using bivariate similarity measurements.

The biomarkers we employed were chosen based on their availability, accessibility, and current medical expertise. However, examining the physiological network using a systematic way would yield a larger and more unbiased set of biomarkers, better characterizing the architecture of the underlying physiology we are attempting to study.

The hospital where we conducted our research is part of the government’s healthcare system. Because this institute is a third-level facility that only accepts referrals, inferring population trends from this dataset would be challenging. Our sample was not subjected to genomic surveillance. National surveillance for the study’s geographical area and period is, however, available (Taboada et al., 2020).

## Conclusion

Relevant physiological biomarkers of disease severity and its outcome can be identified by analyzing data structure in cross-sectional studies considering the interactions between multiple physiological regulation systems. Network analysis indicates the relevance of albumin, blood urea nitrogen, D-dimer, and heart rate correlations in the characteristic network of physiological healthy young men reflecting the homeostatic balance between adaptability and robustness. The SARS-COV-2 virus infection alters this balance as seen in the topological properties of the physiological network, particularly clustering coefficient, anthropometric, metabolic, inflammatory, and pulmonary cluster interaction become disrupted in disease. Because the physiological network of women is more rigid, they have a better prognosis that men under a systemic disease like COVID-19.

## Data Availability Statement

The raw data supporting the conclusions of this article will be made available by the authors, without undue reservation. Requests to access these datasets should be directed to RM, roopamehta@yahoo.com.

## Ethics Statement

The studies involving human participants were reviewed and approved by the Ethics Committee from Instituto Nacional de Ciencias Médicas y Nutrición Salvador Zubiran. The patients/participants provided their written informed consent to participate in this study.

## Author Contributions

RM and CA-S conceptualized the project, designed the protocol, and funding it. AB-M and AR constructed the physiological network model. RM, PA-V, YB, OB-C, NP, BE, CC, MA, JV-G, PV-C, DJ, AV-V, and NA-V participated in data collection. RM, PA-V, NA-V, and AR did the data curation. AB-M, EI-C, and AR performed the data analysis. For results and discussion, AB-M, RF, OR-A, AF, and AR participated in the complexity interpretation, while CA-S, RM, MH, PA-V, NA-V, VM, MA, and IG participated in the medical interpretation. All authors contributed to the article and approved the submitted version.

## Funding

This work was partially funded by UNAM DGAPA PAPIIT IN113619 and PAPIME PE103519 and Consejo Nacional de Ciencia y Tecnología CONACYT Fronteras 610285/2020 and 263377/2020. The funders had no role in study design, data collection and analysis, decision to publish, or preparation of the manuscript.

## Conflict of Interest

The authors declare that the research was conducted in the absence of any commercial or financial relationships that could be construed as a potential conflict of interest.

## Publisher’s Note

All claims expressed in this article are solely those of the authors and do not necessarily represent those of their affiliated organizations, or those of the publisher, the editors and the reviewers. Any product that may be evaluated in this article, or claim that may be made by its manufacturer, is not guaranteed or endorsed by the publisher.
