# Supplementary material for: Physiological Network Is Disrupted in Severe COVID-19
Source: Front Physiol. 2022 Mar 10;13:848172. doi: 10.3389/fphys.2022.848172 (PMC8961032; doi:10.3389/fphys.2022.848172)
Supplement: Supplementary file 1 [file Data_Sheet_1.PDF]

Supplementary Material

1 Supplementary Table 1

Table 1. Criteria in men.

ID	VARIABLE	UNITS	CLINICAL CRITERIA		PROJECT 42*		CLINICAL CRITERIA REFERENCE
			low	High	min	Max	
**D0**	sex: man				1	1	
**D1**	age	years old			18	25	
**P0**	systolic blood pressure (SBP)	mmHg	90	120	90	120	(Whelton et al., 2017)
**P1**	diastolic blood pressure (DBP)	mmHg	60	80	60	80	(Whelton et al., 2017)
**DP0**	pulse pressure (PP)	mmHg	20	60	30	60	(Whelton et al., 2017)
**DP1**	mean arterial pressure (MAP)	mmHg	70	93	70	93	(Whelton et al., 2017)
**T0**	axillary temperature	°C	35.5	37.0	35.5	37.0	(Sund-Levander et al., 2002)
**O2**	oxygen saturation	%	90	100			(O'Driscoll et al., 2017)
**O3**	PAO_2FIO_2 arterial oxygen pressure/inspired fraction of oxygen	mmHg/%	350	450			(Hübler et al., 2014)
**BR**	breathing rate	bpm	8	30			(Metlay et al., 2019)
**HR**	heart rate	bpm	50	100			(Kusumoto Fred M. et al., 2019)

**B0**	weight	kg			46.1	78.5	
**B1**	height	m	1.60		1.60	1.85	(Grimberg et al., 2016)
**DB1**	body mass index (BMI)	kg/m^2	18	25	18	25	(Almeda-Valdes et al., 2016)
**M1**	triglycerides	mg/dL	40	150	40	126	(Baigent et al., 2019)
**M2**	total cholesterol	mg/dL		200	109	200	(Mach et al., 2020)
**M3**	HDL cholesterol	mg/dL	40	90	44.6	67.3	(Mach et al., 2020)
**M4**	LDL cholesterol	mg/dL		116	58	109	(Mach et al., 2020)
**M5**	glucose	mg/dL	70	100	70	94	(Alberti K.G.M.M. et al., 2009)
**M8**	blood urea nitrogen (BUN)	mg/dL	9	23	9	23	(Tyagi & Aeddula, 2021)
**M10**	serum creatinine (SCr)	mg/dL	0.8	1.5	0.8	1.2	(Hosten, 1990)
**M12**	HbA1c	%		5.7	4.7	5.7	(American Diabetes Association, 2020)
**M13**	C-reactive protein (PCR)	mg/dL		1	0.009	0.323	(Oda et al., 2006)
**M20**	direct bilirubin	mg/dL	0	0.3	0.1	0.3	(G.-M. Zhang & Hu, 2018)
**M21**	indirect bilirubin	mg/dL	0.09	0.65	0.09	0.65	(G.-M. Zhang & Hu, 2018)
**M22**	aspartate aminotransferase (AST)	UI/L	5	35	18	28	(Lala Anuradha et al., 2020)

**M23**	alanine transaminase (ALT)	mU/mL	5	35			(Lala et al., 2021)
**M24**	Albumin	g/dL	3.5	5.0			(Kopple et al., 2012)
**M25**	lactic dehydrogenase	U/L	45	90			(Koukourakis et al., 2009)
**M26**	creatinine kinase	U/L	20	215			(Laoutidis & Kioulos, 2014)
**M27**	creatinine kinase MB	ng/mL	5	25			(Kurapati & Soos, 2021)
**M28**	procalcitonin	ng/mL		0.05			(Cleland & Eranki, 2021)
**M29**	ultrasensitive troponin	pg/mL		36			(X. Zhang et al., 2019)
**M30**	natriuretic peptides (BNP)	pg/mL		100			(Potter et al., 2009)
**M31**	interleukin-6 (IL-6)	pg/mL	5	15			(Alecu et al., 1998)
**M32**	thyroid stimulating hormone (TSH)	mU/mL	0.4	4.8			(Khandelwal & Tandon, 2012)
**M33**	vitamin D	ng/mL	30	100			(Sizar et al., 2021)
**M34**	ferritin	ng/mL	20	200			(Wang et al., 2010)
**M35**	dimer D	ng/mL		500			(Bounds & Kok, 2021)
**M36**	prothrombin	s	10	12.5			(Lala et al., 2021)
**M37**	fibrinogen	mg/dL	150	400			(Roshal, 2013)
**DM1**	estimated glomerular filtration rate (eGFR)	ml/min	90	120	90	120	(Musso et al., 2016)

**DM2**	estimated average glucose (eAG)	mg/dL			88	120	(Guo et al., 2020)
**DM3**	eAG-fasting glucose	mg/dL			8	32	(Guo et al., 2020)
**DM4**	BUN to creatinine ratio		8.17		8.18	21.36	(Haines et al., 2019)
**DM5**	METS-IR			51.13	26.80	30.85	(Bello-Chavolla et al., 2018)
**DM6**	TyG index			4.65	4.05	4.65	(Simental-Mendía et al., 2008)
**DM7**	TyG-BMI index			135.4	86.2	115.7	(Lee et al., 2021)
**DM8**	triglycerides-HDL ratio			2.75	1.08	1.72	(Cordero et al., 2008)
**H0**	leukocytes	10^9/L	3.8	10.4	4.4	8.5	(Adeli et al., 2015)
**H1**	total neutrophils	10^9/L	1.9	8.0	2.0	5.9	(Coates, 2021)
**DH1**	total neutrophils percentage	%	40	70	40	70	(Coates, 2021)
**H3**	lymphocytes	10^9/L	1.5	3	1.5	3	(Rosenthal, 2020)
**DH2**	lymphocytes percentage	%	20	50	23	47	(Rosenthal, 2020)
**DH6**	neutrophils-lymphocytes relation		0.78	3.53	0.86	3.02	(Forget et al., 2017)
**H8**	hemoglobin	g/dL	12	18.7	14.8	17.9	(Adeli et al., 2015)
**H14**	platelets	10^3/µL	152	324	159	322	(Adeli et al., 2015)
**H15**	cytopenia	%	0				

**CT1**	average right atrial ventricular epicardial fat	mm			(Eroğlu, 2015)
**CT2**	average left atrial ventricular epicardial fat	mm			(Eroğlu, 2015)
**CT3**	average intraventricular groove epicardial fat	mm			(Eroğlu, 2015)
**CT4**	average epicardial fat	mm	1	9.5	(Iacobellis & Willens, 2009)
**CT5**	main trunk pulmonary artery diameter	mm		29	(Truong et al., 2012)
**CT6**	right main artery diameter	mm		19.8	(Bozlar et al., 2007)
**CT7**	left main artery diameter	mm		22.1	(Bozlar et al., 2007)
**CT8**	thoracic or extra-pericardial fat volume	cm^3			
**CT9**	thoracic subcutaneous adipose tissue	mm			
**CT10**	Hounsfield units	HU	0	20	
**CT11**	epicardial spill on tomography				
**CT12**	extrapericardial fat in tomography				
**CT13**	presence of steatosis on tomography				

**CT14**	type of tomography according to its characteristics<br><br>0=negative,<br>1=typical,<br>2=unspecific,<br>3=atypical
**CT15**	presence of ground glass opacity in tomography
**CT16**	consolidation in tomography
**CT17**	presence of grounded glass opacity and consolidation
**CT18**	presence of aerial bronchogram in tomography
**CT19**	presence of atelectasis in tomography
**CT20**	pulmonary distribution in tomography (0=central, 1=peripheral, 2=both)
**CT21**	lateral involvement in tomography (1=unilateral, 0=bilateral)
**CT22**	pulmonary zone involved in tomography

	(0=upper, 1=central, 2=lower, 3=generalized)		
**CT23**	degree of pulmonary involvement (0=no affection, 1=mild<20%, 2=moderate 20-50%, 3=severe≥50%)		
**CT24**	pulmonary thromboembolia		
**CT25**	evidence of PTE in tomography (1=lobar, 2=segmentary)		
**COM**	number of comorbidities	0	0
**SYMN**	number of symptoms include: fever, cough, dyspnea, myalgias, headache, anosmia, diarrhea, cardiovascular event.	0	0
**SYMD**	days with symptoms before arrival to hospital	0	0

* (Barajas-Martínez et al., 2021)

2 Supplementary Table 2

Table 2. Criteria in women.

ID	VARIABLE	UNITS	CLINICAL CRITERIA		PROJECT 42*		CLINICAL CRITERIA REFERENCE
			low	high	min	Max	
**D0**	sex: woman				0	0	
**D1**	age	years old			18	28	
**P0**	systolic blood pressure (SBP)	mmHg	90	120	90	120	(Whelton et al., 2017)
**P1**	diastolic blood pressure (DBP)	mmHg	60	80	60	80	(Whelton et al., 2017)
**DP0**	pulse pressure (PP)	mmHg	20	60	20	60	(Whelton et al., 2017)
**DP1**	mean arterial pressure (MAP)	mmHg	70	93	70	93	(Whelton et al., 2017)
**T0**	axillary temperature	°C	35.5	37.0	35.5	37.0	(Sund-Levander et al., 2002)
**O2**	oxygen saturation	%	90	100			(O'Driscoll et al., 2017)
**O3**	PAO_2FIO_2 arterial oxygen pressure/inspired fraction of oxygen	mmHg/%	350	450			(Hübler et al., 2014)
**BR**	breathing rate	bpm	8	30			(Metlay et al., 2019)
**HR**	heart rate	bpm	50	100			(Kusumoto Fred M. et al., 2019)
**B0**	weight	kg			43.7	73.2	
**B1**	height	m	1.50		1.50	1.73	(Grimberg et al., 2016)
**DB1**	body mass index (BMI)	kg/m^2	18	25	18	25	(Almeda-Valdes et al., 2016)
**M1**	triglycerides	mg/dL	40	150	44	150	(Baigent et al., 2019)
**M2**	total cholesterol	mg/dL		200	106	200	(Mach et al., 2020)
**M3**	HDL cholesterol	mg/dL	50	90	50	66.4	(Mach et al., 2020)
**M4**	LDL cholesterol	mg/dL		116	50	112	(Mach et al., 2020)
**M5**	glucose	mg/dL	70	100	70	90	(Alberti K.G.M.M. et al., 2009)

**M8**	blood urea nitrogen (BUN)	mg/dL	9	23	9	19	(Tyagi & Aeddula, 2021)
**M10**	serum creatinine (SCr)	mg/dL	0.5	1.1	0.55	0.94	(Hosten, 1990)
**M12**	HbA1c	%		5.7	4.6	5.6	(American Diabetes Association, 2020)
**M13**	C-reactive protein (PCR)	mg/dL		1	0.009	0.323	(Oda et al., 2006)
**M20**	direct bilirubin	mg/dL	0	0.3	0.1	0.3	(G.-M. Zhang & Hu, 2018)
**M21**	indirect bilirubin	mg/dL	0.09	0.65	0.09	0.65	(G.-M. Zhang & Hu, 2018)
**M22**	aspartate aminotransferase (AST)	UI/L	5	35	12	25	(Lala Anuradha et al., 2020)
**M23**	alanine transaminase (ALT)	mU/mL	5	35			(Lala et al., 2021)
**M24**	Albumin	g/dL	3.5	5.0			(Kopple et al., 2012)
**M25**	lactic dehydrogenase	U/L	45	90			(Koukourakis et al., 2009)
**M26**	creatinine kinase	U/L	20	160			(Laoutidis & Kioulos, 2014)
**M27**	creatinine kinase MB	ng/mL	5	25			(Kurapati & Soos, 2021)
**M28**	Procalcitonin	ng/mL		0.05			(Cleland & Eranki, 2021)
**M29**	ultrasensitive troponin	pg/mL		15			(X. Zhang et al., 2019)
**M30**	natriuretic peptides (BNP)	pg/mL		100			(Potter et al., 2009)
**M31**	interleukin-6 (IL-6)	pg/mL	5	15			(Alecu et al., 1998)
**M32**	thyroid stimulating hormone (TSH)	mU/mL	0.4	4.8			(Khandelwal & Tandon, 2012)
**M33**	vitamin D	ng/mL	30	100			(Sizar et al., 2021)
**M34**	ferritin	ng/mL	20	200			(Wang et al., 2010)
**M35**	dimer D	ng/mL		500			(Bounds & Kok, 2021)
**M36**	prothrombin	s	10	13			(Lala et al., 2021)

**M37**	fibrinogen	mg/dL	150	400			(Roshal, 2013)
**DM1**	estimated glomerular filtration rate (eGFR)	ml/min	90	120	90	120	(Musso et al., 2016)
**DM2**	estimated average glucose (eAG)	mg/dL			85	114	(Guo et al., 2020)
**DM3**	eAG-fasting glucose	mg/dL			5.9	37	(Guo et al., 2020)
**DM4**	BUN to creatinine ratio		8.17		10.34	31	(Haines et al., 2019)
**DM5**	METS-IR			51.13	25.61	33.91	(Bello-Chavolla et al., 2018)
**DM6**	TyG index			4.65	4.07	4.65	(Simental-Mendía et al., 2008)
**DM7**	TyG-BMI index			135.4	74.6	108.2	(Lee et al., 2021)
**DM8**	triglycerides-HDL ratio			2.75	0.79	2.47	(Cordero et al., 2008)
**H0**	leukocytes	10^9/L	3.8	10.4	4.6	10.4	(Adeli et al., 2015)
**H1**	total neutrophils	10^9/L	1.9	8.0	1.9	7.0	(Coates, 2021)
**DH1**	total neutrophils percentage	%	40	70	41	70	(Coates, 2021)
**H3**	lymphocytes	10^9/L	1.5	3	1.5	3	(Rosenthal, 2020)
**DH2**	lymphocytes percentage	%	20	50	20	49	(Rosenthal, 2020)
**DH6**	neutrophils-lymphocytes relation		0.78	3.53	0.88	3.53	(Forget et al., 2017)
**H8**	hemoglobin	g/dL	12	16	13	16	(Adeli et al., 2015)
**H14**	Platelets	10^3/µL	153	361	158	357	(Adeli et al., 2015)
**H15**	cytopenia	#	0				
**CT1**	average right atrial ventricular epicardial fat	mm					(Eroğlu, 2015)
**CT2**	average left atrial ventricular epicardial fat	mm					(Eroğlu, 2015)
**CT3**	average intraventricular	mm					(Eroğlu, 2015)

	groove epicardial fat					
**CT4**	average epicardial fat	mm	1	7.5		(Iacobellis & Willens, 2009)
**CT5**	main trunk pulmonary artery diameter	mm		27		(Truong et al., 2012)
**CT6**	right main artery diameter	mm		19.8		(Bozlar et al., 2007)
**CT7**	left main artery diameter	mm		22.1		(Bozlar et al., 2007)
**CT8**	thoracic or extra-pericardial fat volume	cm^3				
**CT9**	thoracic subcutaneous adipose tissue	mm				
**CT10**	Hounsfield units	HU	0	20		
**CT11**	epicardial spill on tomography					
**CT12**	extrapericardial fat in tomography					
**CT13**	presence of steatosis on tomography					
**CT14**	type of tomography according to its characteristics: 0=negative, 1=typical, 2=unspecific, 3=atypical					
**CT15**	presence of ground glass opacity in tomography					
**CT16**	consolidation in tomography					
**CT17**	presence of grounded glass opacity and consolidation					

**CT18**	presence of aerial bronchogram in tomography		
**CT19**	presence of atelectasis in tomography		
**CT20**	pulmonary distribution in tomography		
**CT21**	lateral involvement in tomography		
**CT22**	pulmonary zone involved in tomography (0=upper, 1=central, 2=lower, 3=generalized)		
**CT23**	degree of pulmonary involvement (0=no affection, 1=mild<20%, 2=moderate 20-50%, 3=severe≥50%)		
**CT24**	pulmonary thromboembolia		
**CT25**	evidence of PTE in tomography (1=lobar, 2=segmentary)		
**COM**	number of comorbidities	0	0
**SYMN**	number of symptoms include: fever, cough, dyspnea, myalgias, headache, anosmia, diarrhea, cardiovascular event.	0	0

**SYMD**	days with symptoms before arrival to hospital	0	0
**VSO**	supplemental oxygen therapy		
**VS1**	number of days with ventilatory support		

* (Barajas-Martínez et al., 2021)

Adeli, K., Raizman, J. E., Chen, Y., Higgins, V., Nieuwesteeg, M., Abdelhaleem, M., Wong, S. L., & Blais, D. (2015). Complex Biological Profile of Hematologic Markers across Pediatric, Adult, and Geriatric Ages: Establishment of Robust Pediatric and Adult Reference Intervals on the Basis of the Canadian Health Measures Survey. *Clinical Chemistry*, *61*(8), 1075–1086. https://doi.org/10.1373/clinchem.2015.240531

Alberti K.G.M.M., Eckel Robert H., Grundy Scott M., Zimmet Paul Z., Cleeman James I., Donato Karen A., Fruchart Jean-Charles, James W. Philip T., Loria Catherine M., & Smith Sidney C. (2009). Harmonizing the Metabolic Syndrome. *Circulation*, *120*(16), 1640–1645. https://doi.org/10.1161/CIRCULATIONAHA.109.192644

Alecu, M., Geleriu, L., Coman, G., & Gălățescu, L. (1998). The interleukin-1, interleukin-2, interleukin-6 and tumour necrosis factor alpha serological levels in localised and systemic sclerosis. *Romanian Journal of Internal Medicine = Revue Roumaine de Medecine Interne*, *36*(3–4), 251–259.

Almeda-Valdes, P., Aguilar-Salinas, C. A., Uribe, M., Canizales-Quinteros, S., & Méndez-Sánchez, N. (2016). Impact of anthropometric cut-off values in determining the prevalence of metabolic alterations. *European Journal of Clinical Investigation*, *46*(11), 940–946. https://doi.org/10.1111/eci.12672

American Diabetes Association, A. D. (2020). 6. Glycemic Targets: Standards of Medical Care in Diabetes—2020. *Diabetes Care*, *43*(Supplement 1), S66–S76. https://doi.org/10.2337/dc20-S006

Baigent, C., Mach, F., Halliday, A., Mihaylova, B., Catapano, A. L., & Ference, B. A. (2019). 2019 ESC/EAS guidelines for the management of dyslipidaemias: Lipid modification to reduce cardiovascular risk. The Task Force for the management of dyslipidaemias of the European Society of Cardiology (ESC) and European Atherosclerosis Society (EAS). *European Heart Journal*, *41*(1).

Barajas-Martínez, A., Ibarra-Coronado, E., Sierra-Vargas, M. P., Cruz-Bautista, I., Almeda-Valdes, P., Aguilar-Salinas, C. A., Fossion, R., Stephens, C. R., Vargas-Domínguez, C., Atzatzi-Aguilar, O. G., Debray-García, Y., García-Torrentera, R., Bobadilla, K., Naranjo Meneses, M. A., Mena Orozco, D. A., Lam-Chung, C. E., Martínez Garcés, V., Lecona, O. A., Marín-García, A. O., … Rivera, A. L. (2021). Physiological Network From Anthropometric and Blood Test Biomarkers. *Frontiers in Physiology*, *11*. https://doi.org/10.3389/fphys.2020.612598

Bello-Chavolla, O. Y., Almeda-Valdes, P., Gomez-Velasco, D., Viveros-Ruiz, T., Cruz-Bautista, I., Romo-Romo, A., Sánchez-Lázaro, D., Meza-Oviedo, D., Vargas-Vázquez, A., Campos, O. A., Sevilla-González, M. del R., Martagón, A. J., Hernández, L. M., Mehta, R., Caballeros-Barragán, C. R., & Aguilar-Salinas, C. A. (2018). METS-IR, a novel score to evaluate insulin sensitivity, is predictive of visceral adiposity and incident type 2 diabetes. *European Journal of Endocrinology*, *178*(5), 533–544. https://doi.org/10.1530/EJE-17-0883

Bounds, E. J., & Kok, S. J. (2021). D Dimer. En *StatPearls*. StatPearls Publishing. http://www.ncbi.nlm.nih.gov/books/NBK431064/

Bozlar, U., Ors, F., Deniz, O., Uzun, M., Gumus, S., Ugurel, M. S., Yazar, F., & Tayfun, C. (2007). Pulmonary Artery Diameters Measured by Multidetector-Row Computed Tomography in Healthy Adults. *Acta Radiologica*, *48*(10), 1086–1091. https://doi.org/10.1080/02841850701545755

Cleland, D. A., & Eranki, A. P. (2021). Procalcitonin. En *StatPearls*. StatPearls Publishing. http://www.ncbi.nlm.nih.gov/books/NBK539794/

Coates, T. D. (2021). *Approach to the patient with neutrophilia—UpToDate*. https://www-uptodate-com.pbidi.unam.mx:2443/contents/approach-to-the-patient-with-neutrophilia

Cordero, A., Laclaustra, M., León, M., Casasnovas, J. A., Grima, A., Luengo, E., Ordoñez, B., Bergua, C., Bes, M., Pascual, I., Alegría, E., & MESYAS Registry Investigators. (2008). Comparison of serum lipid values in subjects with and without the metabolic syndrome. *The American Journal of Cardiology*, *102*(4), 424–428. https://doi.org/10.1016/j.amjcard.2008.03.079

Eroğlu, S. (2015). How do we measure epicardial adipose tissue thickness by transthoracic echocardiography? *Anatolian Journal of Cardiology*, *15*(5), 416–419. https://doi.org/10.5152/akd.2015.5991

Forget, P., Khalifa, C., Defour, J.-P., Latinne, D., Van Pel, M.-C., & De Kock, M. (2017). What is the normal value of the neutrophil-to-lymphocyte ratio? *BMC Research Notes*, *10*(1), 12. https://doi.org/10.1186/s13104-016-2335-5

Grimberg, A., DiVall, S. A., Polychronakos, C., Allen, D. B., Cohen, L. E., Quintos, J. B., Rossi, W. C., Feudtner, C., Murad, M. H., & Society, on behalf of the D. and T. C. and E. C. of the P. E. (2016). Guidelines for Growth Hormone and Insulin-Like Growth Factor-I Treatment in Children and Adolescents: Growth Hormone Deficiency, Idiopathic Short Stature, and Primary Insulin-Like Growth Factor-I Deficiency. *Hormone Research in Paediatrics*, *86*(6), 361–397. https://doi.org/10.1159/000452150

Guo, J., Lei, S., Zhou, Y., & Pan, C. (2020). The ratio of estimated average glucose to fasting plasma glucose level as an indicator of insulin resistance in young adult diabetes. *Medicine*, *99*(40). https://doi.org/10.1097/MD.0000000000022337

Haines, R. W., Zolfaghari, P., Wan, Y., Pearse, R. M., Puthucheary, Z., & Prowle, J. R. (2019). Elevated urea-to-creatinine ratio provides a biochemical signature of muscle catabolism and persistent critical illness after major trauma. *Intensive Care Medicine*, *45*(12), 1718–1731. https://doi.org/10.1007/s00134-019-05760-5

Hosten, A. O. (1990). BUN and Creatinine. En H. K. Walker, W. D. Hall, & J. W. Hurst (Eds.), *Clinical Methods: The History, Physical, and Laboratory Examinations* (3rd ed.). Butterworths. http://www.ncbi.nlm.nih.gov/books/NBK305/

Hübler, M., Koch, T., & Domino, K. B. (2014). *Complications and Mishaps in Anesthesia: Cases – Analysis – Preventive Strategies*. Springer.

Iacobellis, G., & Willens, H. J. (2009). Echocardiographic Epicardial Fat: A Review of Research and Clinical Applications. *Journal of the American Society of Echocardiography*, *22*(12), 1311–1319. https://doi.org/10.1016/j.echo.2009.10.013

Khandelwal, D., & Tandon, N. (2012). Overt and Subclinical Hypothyroidism. *Drugs*, *72*(1), 17–33. https://doi.org/10.2165/11598070-000000000-00000

Kopple, J. D., Massry, S. G., & Kalantar-Zadeh, K. (2012). *Nutritional Management of Renal Disease*. Academic Press.

Koukourakis, M. I., Kontomanolis, E., Giatromanolaki, A., Sivridis, E., & Liberis, V. (2009). Serum and Tissue LDH Levels in Patients with Breast/Gynaecological Cancer and Benign Diseases. *Gynecologic and Obstetric Investigation*, *67*(3), 162–168. https://doi.org/10.1159/000183250

Kurapati, R., & Soos, M. P. (2021). CPK-MB. En *StatPearls*. StatPearls Publishing. http://www.ncbi.nlm.nih.gov/books/NBK557591/

Kusumoto Fred M., Schoenfeld Mark H., Barrett Coletta, Edgerton James R., Ellenbogen Kenneth A., Gold Michael R., Goldschlager Nora F., Hamilton Robert M., Joglar José A., Kim Robert J., Lee Richard, Marine Joseph E., McLeod Christopher J., Oken Keith R., Patton Kristen K., Pellegrini Cara N., Selzman Kimberly A., Thompson Annemarie, & Varosy Paul D. (2019). 2018 ACC/AHA/HRS Guideline on the Evaluation and Management of Patients With Bradycardia and Cardiac Conduction Delay: Executive Summary. *Journal of the American College of Cardiology*, *74*(7), 932–987. https://doi.org/10.1016/j.jacc.2018.10.043

Lala Anuradha, Johnson Kipp W., Januzzi James L., Russak Adam J., Paranjpe Ishan, Richter Felix, Zhao Shan, Somani Sulaiman, Van Vleck Tielman, Vaid Akhil, Chaudhry Fayzan, De Freitas Jessica K., Fayad Zahi A., Pinney Sean P., Levin Matthew, Charney Alexander, Bagiella Emilia, Narula Jagat, Glicksberg Benjamin S., … null null. (2020). Prevalence and Impact of Myocardial Injury in Patients Hospitalized With COVID-19 Infection. *Journal of the American College of Cardiology*, *76*(5), 533–546. https://doi.org/10.1016/j.jacc.2020.06.007

Lala, V., Goyal, A., Bansal, P., & Minter, D. A. (2021). Liver Function Tests. En *StatPearls*. StatPearls Publishing. http://www.ncbi.nlm.nih.gov/books/NBK482489/

Laoutidis, Z. G., & Kioulos, K. T. (2014). Antipsychotic-induced elevation of creatine kinase: A systematic review of the literature and recommendations for the clinical practice. *Psychopharmacology*, *231*(22), 4255–4270. https://doi.org/10.1007/s00213-014-3764-2

Lee, J., Kim, B., Kim, W., Ahn, C., Choi, H. Y., Kim, J. G., Kim, J., Shin, H., Kang, J. G., & Moon, S. (2021). Lipid indices as simple and clinically useful surrogate markers for insulin resistance in the U.S. population. *Scientific Reports*, *11*(1), 2366. https://doi.org/10/gjh56j

Mach, F., Baigent, C., Catapano, A. L., Koskinas, K. C., Casula, M., Badimon, L., Chapman, M. J., Backer, G. G. D., Delgado, V., Ference, B. A., Graham, I. M., Halliday, A., Landmesser, U., Mihaylova, B., Pedersen, T. R., Riccardi, G., Richter, D. J., Sabatine, M. S., Taskinen, M.-R., … Wiklund, O. (2020). 2019 ESC/EAS Guidelines for themanagement of dyslipidaemias: Lipid modification to reduce cardiovascular risk. *Russian Journal of Cardiology*, *25*(5), 3826. https://doi.org/10.15829/1560-4071-2020-3826

Metlay, J. P., Waterer, G. W., Long, A. C., Anzueto, A., Brozek, J., Crothers, K., Cooley, L. A., Dean, N. C., Fine, M. J., Flanders, S. A., Griffin, M. R., Metersky, M. L., Musher, D. M., Restrepo, M. I., & Whitney, C. G. (2019). Diagnosis and Treatment of Adults with Community-acquired Pneumonia. An Official Clinical Practice Guideline of the American Thoracic Society and Infectious Diseases Society of America. *American Journal of Respiratory and Critical Care Medicine*, *200*(7), e45–e67. https://doi.org/10.1164/rccm.201908-1581ST

Musso, C. G., Álvarez-Gregori, J., Jauregui, J., & Macías-Núñez, J. F. (2016). Glomerular filtration rate equations: A comprehensive review. *International Urology and Nephrology*, *48*(7), 1105–1110. https://doi.org/10.1007/s11255-016-1276-1

Oda, E., Oohara, K., Abe, A., Veeraveedu, P. T., Watanabe, K., Kato, K., & Aizawa, Y. (2006). The Optimal Cut-off Point of C-Reactive Protein as an Optional Component of Metabolic Syndrome in Japan. *Circulation Journal*, *70*(4), 384–388. https://doi.org/10.1253/circj.70.384

O'Driscoll, B. R., Howard, L. S., Earis, J., & Mak, V. (2017). BTS guideline for oxygen use in adults in healthcare and emergency settings. *Thorax*, *72*(Suppl 1), ii1–ii90. https://doi.org/10.1136/thoraxjnl-2016-209729

Potter, L. R., Yoder, A. R., Flora, D. R., Antos, L. K., & Dickey, D. M. (2009). Natriuretic Peptides: Their Structures, Receptors, Physiologic Functions and Therapeutic Applications. En H. H. H. W. Schmidt, F. Hofmann, & J.-P. Stasch (Eds.), *CGMP: Generators, Effectors and Therapeutic Implications* (pp. 341–366). Springer. https://doi.org/10.1007/978-3-540-68964-5_15

Rosenthal, D. S. (2020). *Evaluation of the peripheral blood smear—UpToDate*. https://www-uptodate-com.pbidi.unam.mx:2443/contents/evaluation-of-the-peripheral-blood-smear

Roshal, M. (2013). Chapter 123—Thrombin Time and Fibrinogen Determination. En B. H. Shaz, C. D. Hillyer, M. Roshal, & C. S. Abrams (Eds.), *Transfusion Medicine and Hemostasis (Second Edition)* (pp. 793–798). Elsevier. https://doi.org/10.1016/B978-0-12-397164-7.00123-3

Simental-Mendía, L. E., Rodríguez-Morán, M., & Guerrero-Romero, F. (2008). The product of fasting glucose and triglycerides as surrogate for identifying insulin resistance in apparently healthy subjects. *Metabolic Syndrome and Related Disorders*, *6*(4), 299–304. https://doi.org/10.1089/met.2008.0034

Sizar, O., Khare, S., Goyal, A., Bansal, P., & Givler, A. (2021). Vitamin D Deficiency. En *StatPearls*. StatPearls Publishing. http://www.ncbi.nlm.nih.gov/books/NBK532266/

Sund-Levander, M., Forsberg, C., & Wahren, L. K. (2002). Normal oral, rectal, tympanic and axillary body temperature in adult men and women: A systematic literature review. *Scandinavian Journal of Caring Sciences*, *16*(2), 122–128. https://doi.org/10.1046/j.1471-6712.2002.00069.x

Truong, Q. A., Massaro Joseph M., Rogers Ian S., Mahabadi Amir A., Kriegel Matthias F., Fox Caroline S., O'Donnell Christopher J., & Hoffmann Udo. (2012). Reference Values for Normal Pulmonary Artery Dimensions by Noncontrast Cardiac Computed Tomography. *Circulation: Cardiovascular Imaging*, *5*(1), 147–154. https://doi.org/10.1161/CIRCIMAGING.111.968610

Tyagi, A., & Aeddula, N. R. (2021). Azotemia. En *StatPearls*. StatPearls Publishing. http://www.ncbi.nlm.nih.gov/books/NBK538145/

Wang, W., Knovich, M. A., Coffman, L. G., Torti, F. M., & Torti, S. V. (2010). Serum ferritin: Past, present and future. *Biochimica et Biophysica Acta (BBA) - General Subjects*, *1800*(8), 760–769. https://doi.org/10.1016/j.bbagen.2010.03.011

Whelton, P., Carey, R., & Aronow, W. (2017). Guideline for high blood pressure in adults. *American College of Cardiology*, 1–56.

Zhang, G.-M., & Hu, Z.-D. (2018). Conjugated bilirubin as a reflex test for increased total bilirubin in apparently healthy population. *Journal of Clinical Laboratory Analysis*, *32*(2), e22233. https://doi.org/10.1002/jcla.22233

Zhang, X., Lv, H., Li, Y., Zhang, C., Wang, P., Liu, Q., Ai, B., Xu, Z., & Zhao, Z. (2019). Ultrasensitive sandwich-type immunosensor for cardiac troponin I based on enhanced electrocatalytic reduction of H2O2 using β-cyclodextrins functionalized 3D porous graphene-supported Pd@Au nanocubes. *Journal of Materials Chemistry B*, *7*(9), 1460–1468. https://doi.org/10.1039/C8TB03362E

3 Supplementary Table 3

	Number of observations						Mean						Standard deviation						Skewness						Excess kurtosis						α					
	Full	FYR	MYR	MYD	MOR	MOD	Full	FYR	MYR	MYD	MOR	MOD	Full	FYR	MYR	MYD	MOR	MOD	Full	FYR	MYR	MYD	MOR	MOD	Full	FYR	MYR	MYD	MOR	MOD	Full	FYR	MYR	MYD	MOR	MOD
albumin	689	156	250	72	63	47	0.1	0.2	0.2	-0.03	0.1	-0.1	0.3	0.3	0.3	0.3	0.3	0.2	0.3	-0.1	-0.1	-0.1	0.7	-0.3	1.6	0.4	-0.3	0.1	0.2	-0.6	3	1.4	1.4	8.7	4.5	2.2
oxygen saturation	699	160	256	72	63	47	-0.9	-0.4	-0.5	-1.8	-0.7	-2.2	1.3	0.9	1	1.8	1.1	1.7	-1.6	-1.6	-2.3	-0.9	-2.4	-0.02	2.4	3.4	6.5	-0.1	7.3	-1.3	3.3	4.3	7.1	1.4	7.8	1.5
arterial pressure/inspired fraction of O_2	660	152	232	72	60	47	-1.3	-0.9	-1.2	-1.8	-1.3	-2	1	1	0.9	0.9	0.9	0.9	0.7	0.7	0.6	1.3	0.4	1	0.9	1.7	0.9	1.9	0.1	-0.1	1.4	2.2	1.3	2.3	0.8	1.1
est. glomerular filtration rate	688	157	248	72	63	47	-0.6	0.2	-0.6	-1	-1.1	-1.4	0.9	0.8	0.8	0.8	0.7	0.7	-0.3	-1.3	-0.2	0.2	-0.3	0.2	-0.4	3.4	-0.3	-0.5	-0.6	-0.8	1.7	5.6	1.4	1	1	1
lymphocytes	700	161	256	72	63	47	-0.5	-0.4	-0.5	-0.6	-0.6	-0.6	0.3	0.4	0.3	0.3	0.3	0.2	0.6	0.4	0.4	0.5	0.3	0.6	0.9	0.6	0.8	0.3	0.1	0.9	1.3	1.2	1.2	0.8	0.6	1.2
thoracic subcutaneous adipose tissue	700	161	256	72	63	47	0.3	0.4	0.2	0.3	0.2	0.2	0.2	0.1	0.1	0.1	0.1	0.1	1	0.7	1.4	1.9	1	0.5	1.5	1.5	2.8	6.7	0.5	-0.6	1.9	1.7	3.2	7	1.3	1
TyG-BMI index	426	103	130	56	39	35	1.3	1.3	1.2	1.5	1.1	1.2	0.5	0.5	0.4	0.6	0.4	0.4	1.4	1.4	0.7	1	0.9	0.9	4.3	4.9	0.8	1.4	0.8	0.6	4.5	5.1	1.1	1.8	1.2	1.1
body mass index	648	148	234	68	60	47	1.7	1.9	1.7	2	1.6	1.5	0.8	0.9	0.7	0.9	0.7	0.6	1.4	1.1	0.8	0.8	1.1	0.6	4.5	2.4	1.6	1.8	2.1	0.4	4.7	2.6	1.8	2	2.4	0.8
weight	656	148	237	70	60	47	1.1	1.1	1.2	1.4	1.1	1	0.5	0.6	0.5	0.6	0.4	0.3	1.2	1.3	1	1.1	0.3	0.6	3.5	3.4	2.6	2.1	-0.5	-0.5	3.8	3.7	2.8	2.4	0.7	0.9
hepatic steatosis	700	161	256	72	63	47	0.4	0.4	0.4	0.5	0.1	0.3	0.5	0.5	0.5	0.5	0.4	0.4	0.7	0.5	0.6	0	2	1.1	-1.5	-1.8	-1.4	-2	2	-0.8	2.1	2.2	2	2.3	3.8	2.2
heart rate	699	160	256	72	63	47	1	1	1.1	1.1	0.8	1.1	0.4	0.4	0.3	0.4	0.4	0.3	-0.9	-1.2	-0.05	-1.7	-0.7	-0.1	3.1	5.1	-0.3	4.8	1.2	0.02	3.2	5.2	0.4	5.1	1.5	0.3
axillary temperature	692	159	254	70	63	45	1.1	1.1	1.2	1.2	1	1.1	0.6	0.6	0.6	0.6	0.5	0.5	0.5	0.3	0.6	0.4	0.5	0.2	-0.1	-0.5	-0.4	-0.6	0.1	-0.6	0.8	0.8	0.8	0.9	0.7	0.8
prothrombin	662	150	238	70	60	45	0.8	0.5	0.8	0.9	1.6	1.8	2.5	0.4	0.5	1.7	5.5	6.5	15.1	1.3	0.8	7.3	6.4	6.1	247.8	3.4	0.5	55.3	42.8	37	248.3	3.7	1.1	55.8	43.4	37.7
vitamin D	522	113	193	54	49	35	-0.1	-0.1	-0.1	-0.1	-0.1	-0.1	0.1	0.1	0.1	0.1	0.1	0.1	1.5	1.1	1.6	0.01	0.7	0.5	5.2	1.7	6.2	-0.8	-0.01	-0.1	5.5	2.2	6.6	1.2	2.1	0.8
height	649	147	235	68	61	47	0.3	0.3	0.4	0.3	0.3	0.2	0.3	0.3	0.3	0.3	0.3	0.2	0.1	0.2	0.2	-0.5	-0.1	0.3	-0.2	-0.5	-0.2	0.2	-0.8	-0.2	1	1	0.8	1	1.3	1.3
hemoglobin	688	156	249	72	63	47	0.7	1.2	0.6	0.6	0.6	0.5	2.8	5.9	0.2	0.2	0.3	0.2	19.9	9.4	-1.1	-0.2	-0.9	-1.2	420.9	92.1	4	2	2.6	3.3	421.3	92.8	4.2	2	2.8	3.5
diastolic pressure	693	159	255	70	63	45	0.7	0.7	0.9	0.8	0.7	0.7	0.6	0.5	0.5	0.5	0.6	0.6	0.2	0.4	0.3	-0.1	0.04	0.6	0.2	0.4	0.3	-0.3	-0.2	0.2	0.8	0.9	0.7	0.7	0.8	1.2
mean arterial pressure	692	159	255	70	63	44	0.9	0.9	1	0.9	1	0.8	0.5	0.5	0.5	0.5	0.5	0.6	0.3	0.5	0.4	-0.1	0.3	1	0.4	0.5	0.5	-0.7	0.5	1.4	0.8	0.9	0.7	0.9	0.8	1.8
systolic pressure	693	159	255	70	63	45	1.1	1.1	1.1	1	1.2	1.1	0.5	0.5	0.5	0.6	0.5	0.6	0.3	0.5	0.5	-0.3	0.6	0.8	0.4	-0.03	1	-0.7	0.8	0.9	0.7	0.7	1.2	0.9	1.1	1.3
pulse pressure	692	159	255	70	63	44	0.7	0.7	0.6	0.6	0.8	0.7	0.3	0.3	0.3	0.4	0.4	0.4	0.5	0.7	0.4	0.04	1	0.1	0.9	1.5	0.6	-0.3	2.3	-0.6	1.2	1.7	0.9	0.7	2.6	0.8
cytopenia	686	156	248	72	63	46	0.4	0.4	0.4	0.3	0.4	0.3	0.6	0.6	0.6	0.5	0.7	0.6	1.5	1.7	1.4	1.3	1.9	1.6	2.1	2.4	1.8	0.4	3.3	1.5	3	3.4	2.8	2.1	4.2	2.8

serum creatinine	688	157	248	72	63	47	1.3	1.2	0.5	1.1	0.8	1.3	12.3	8.1	1.5	3.8	2	4.1	22	11.8	10.5	7.6	4.6	6.1	521.8	140.3	126	58.9	22	37.1	522.3	141	126.5	59.5	22.6	37.8
consolidation	694	159	253	72	63	47	0.5	0.5	0.6	0.5	0.3	0.5	0.5	0.5	0.5	0.5	0.5	0.5	-0.1	-0.2	-0.2	-0.2	0.8	-0.1	-1.9	-2	-2	-2	-1.3	-2	2.1	2.2	2.2	2.2	2.2	2.2
grounded glass opacity	694	159	253	72	63	47	0.5	0.5	0.6	0.5	0.3	0.5	0.5	0.5	0.5	0.5	0.5	0.5	-0.1	-0.2	-0.2	-0.2	0.8	-0.1	-2	-2	-2	-2	-1.3	-2	2.2	2.2	2.2	2.2	2.2	2.2
ventilatory support time	700	161	256	72	63	47	-0.03	-0.1	-0.03	0.1	-0.1	-0.03	0.2	0.2	0.3	0.3	0.2	0.3	2.9	3.2	2.8	1.8	4	3.4	8.7	10.5	7.6	3	18.3	11.5	12.1	11.5	11.8	5.5	19	14.4
hospitalization time	700	161	256	72	63	47	0.6	0.5	0.8	0.6	0.9	0.5	1.3	1.2	1.4	1	1.6	1	2	1.9	1.6	1.8	2.3	2.1	4.8	4	2.3	3.8	6.6	4.2	5.6	5	3.3	4.5	7.2	5
platelets	688	156	249	72	63	47	0.4	0.4	0.5	0.5	0.5	0.4	0.5	0.5	0.5	0.5	0.5	0.4	1.3	1	1.6	0.6	1.5	0.7	3.9	2.5	5.7	-0.3	3.7	0.4	4.3	3	6	1.2	4.2	1.3
symptomatic time	700	161	256	72	63	47	0.6	0.5	0.6	0.6	0.7	0.6	0.3	0.3	0.3	0.3	0.4	0.3	1	0.6	0.7	1.2	0.6	1.1	1.8	0.3	0.9	1.9	-0.4	2	2.1	0.9	1.2	2.3	0.9	2.3
indirect bilirubin	686	156	249	71	63	47	0.7	0.5	0.7	0.7	0.9	0.7	0.4	0.3	0.5	0.3	0.6	0.4	3.3	1.7	3.5	1.4	3.6	1.6	19.4	4.1	18.1	1.9	16.3	3.8	19.7	4.5	18.5	2.4	16.7	4.2
direct bilirubin	687	156	250	71	63	47	0.8	0.5	0.8	0.9	1	0.8	0.9	0.3	1	0.7	1.9	0.4	8.1	2.2	4.5	2.8	6.5	1.1	100.3	7.2	22.7	10.2	45.1	0.5	100.6	7.6	23.2	10.6	45.6	1.3
epicardial fat	700	161	256	72	63	47	2.1	1.4	2.3	2.7	2.7	2.8	1	0.8	0.8	1	1.2	1.1	1.6	6.7	0.2	0.2	2.5	0.7	8	67.6	0.1	-0.6	12.2	0.2	8.2	67.9	0.4	0.7	12.5	0.8
creatinine kinase	654	150	237	70	61	44	1	0.5	1.3	1.6	1.1	1.1	1.6	0.7	2.2	1.9	1	1.2	5.4	2.7	5.1	2.1	1.4	2	46.1	9.1	35.6	4.3	1.6	4.4	46.5	9.5	36.1	5	2.3	5
alanine transaminase	688	156	250	71	63	47	1.6	1.4	1.6	2.5	1.5	1.8	3	2.9	1.4	6.6	2	4.1	11.4	10	2.7	6.7	4.1	6.2	164.4	111.3	9.5	47.4	18.9	37.8	164.8	111.8	9.9	48	19.4	38.3
aspartate aminotransferase	688	156	250	71	63	47	1.9	1.3	1.6	4.1	2.1	2.4	4.9	1.6	1.3	13.8	3.1	5	16.1	6.1	3.3	6.3	4.7	6.2	308.6	44	15.6	40.7	24.2	38	309	44.4	16	41.3	24.7	38.6
ferritin	677	154	243	71	62	47	4.4	2.3	4.9	7.4	4.9	6.2	6.2	6.8	3.5	8.8	3.9	11.7	8	10.9	1.6	4.6	1.5	6	88.7	126.1	4.3	27.1	3.3	36.6	89	126.6	4.6	27.5	3.7	37.1
lactic dehydrogenase	700	161	256	72	63	47	1.3	0.9	1.2	2.3	1.3	2	1.3	0.6	0.9	2.8	0.6	1.7	7	0.8	1.9	4.4	0.7	4.9	79.6	1.9	10.4	20.8	0.1	27.7	79.9	2.2	10.6	21.3	0.8	28.1
respiratory rate	663	160	245	58	60	40	0.9	0.8	0.8	1.1	0.9	1	0.4	0.5	0.3	0.3	0.3	0.3	3.9	4.1	0.5	-0.5	0.3	-0.5	30.4	23.2	-0.6	-1	-0.8	0.3	30.7	23.5	0.8	1.1	1	0.7
pulmonary involvement	700	161	256	72	63	47	2.3	2	2.2	2.7	2.3	2.9	0.8	0.8	0.7	0.6	0.7	0.3	-0.7	-0.3	-0.6	-2	-0.6	-2.9	-0.4	-0.7	-0.3	2.8	-0.9	6.4	0.9	0.8	0.7	3.4	1.1	7
fibrinogen	635	144	227	70	59	41	2.1	1.8	2.2	2.4	2	2.5	0.8	0.7	0.8	0.8	0.8	0.6	0.1	0.6	-0.2	-0.4	-0.1	0.1	-0.8	-0.1	-0.7	-0.6	-1.1	-1.1	0.9	0.7	0.8	0.8	1.2	1.1
C-reactive protein	700	161	256	72	63	47	5.7	3.8	4.8	10	5.4	12.3	13	2.9	4.7	28.7	3.2	31.6	16.2	0.8	6	8	0.2	6.4	285.6	-0.1	65.2	63.2	-1	39.5	286	1.1	65.4	63.8	1.2	40.1
neutrophils-lymphocytes ratio	658	145	243	68	60	46	4	2.4	3.4	5.9	4.3	7.5	4.9	2.5	3.8	6.4	4.1	8	3.9	2.5	3.8	2.6	2	3.2	22	7.4	20	7.8	4.5	13	22.4	7.9	20.3	8.3	5	13.5
leukocytes	688	156	249	72	63	47	0.8	0.5	0.7	1.1	0.7	1.1	0.7	0.8	0.6	0.8	0.6	0.9	2.9	7.3	1.5	0.9	1.2	1.1	20.3	70.7	2.9	1.6	1	0.3	20.5	71	3.4	2	1.8	1.4
total neutrophils	700	161	256	72	63	47	0.7	0.5	0.6	1.1	0.7	1.1	0.8	0.9	0.7	0.9	0.7	1	2.6	6.6	1.1	0.6	0.9	0.9	17.5	63.5	2.3	1.2	0.6	0.2	17.8	63.8	2.8	1.6	1.5	1.3
triglycerides	455	108	146	57	40	36	1.2	1.1	1.2	1.3	1.1	1.2	0.8	0.6	0.8	0.7	0.7	1.2	2.8	1.3	2.2	1.5	1.8	3.7	12.7	1.9	6.2	2.4	4.7	15.4	13	2.4	6.6	2.9	5.1	15.8
TyG index	453	108	145	57	40	35	1.5	1.4	1.5	1.8	1.5	1.7	0.5	0.5	0.5	0.6	0.6	0.6	0.9	0.7	0.9	0.8	1.1	0.7	0.7	0.2	1.5	-0.5	1.6	-0.6	1.2	0.8	1.8	1	2	1
glucose	687	157	248	72	63	46	2.8	2.1	2.2	4.3	3	4.9	3	2.5	2.4	3.9	2.9	4.8	2.4	2.6	3.2	1.3	2.3	1.6	6.6	7.1	14.2	0.9	6	1.5	7.1	7.6	14.6	1.8	6.5	2.4
blood urea nitrogen	688	157	248	72	63	47	0.7	0.3	0.5	1.1	1.2	1.6	1.1	0.6	0.8	1.5	1.5	1.2	4.1	4	5.9	4.8	3.4	1.2	26.7	22.6	54	28.7	13.4	1.2	27.1	23	54.3	29.1	13.9	1.9

BUN to creatinine ratio	688	157	248	72	63	47	0.4	0.4	0.3	0.4	0.5	0.6	0.4	0.5	0.3	0.3	0.3	0.3	5	7.4	1.5	1.2	0.8	0.2	63.5	73.8	4.3	1.9	0.3	0.1	63.7	74.1	4.7	2.4	1	0.6
D dimer	676	154	247	69	61	46	4.3	2.5	3.9	8.6	2.7	8.1	15.5	7.2	18.3	27	2.5	17.7	10	8.9	10	5.6	1.8	3.7	113.7	89.4	104	34.7	2.9	14.1	114.2	89.8	104.5	35.3	3.5	14.7
ultrasensitive troponin	627	141	222	66	56	46	2	0.5	0.8	2.4	2.2	2.6	11.9	1.6	7.1	5.8	14.5	6.2	9.8	9	14.1	4	7.1	3.8	102.9	88.3	203.7	18.7	49.1	16.2	103.6	88.9	204.4	19.3	50	16.9
age	700	161	256	72	63	47	0.8	0.6	0.6	0.7	1.1	1.2	0.3	0.2	0.2	0.2	0.1	0.2	0.1	-0.3	-0.4	-0.8	1.2	1.3	-0.4	-0.9	-0.7	0.4	1.2	1.8	0.6	1.1	0.9	0.9	1.7	2.2
right main pulmonary artery	700	161	256	72	63	47	1.2	1	1.2	1.4	1.4	1.6	0.4	0.3	0.4	0.3	0.4	0.4	0.3	0.3	0.5	-0.4	0.3	-0.5	0.1	-0.3	0.5	1.2	0.1	-0.6	0.5	0.5	0.8	1.3	0.5	0.8
left main pulmonary artery	700	161	256	72	63	47	0.9	0.8	0.9	1.1	1.1	1.2	0.3	0.2	0.3	0.2	0.3	0.3	0.5	0.2	0.5	0.1	0.7	0.1	0.7	0.4	0.02	0.5	1.2	-0.6	0.9	0.6	0.6	0.6	1.4	0.7
pulmonary artery main trunk	700	161	256	72	63	47	1	1	0.9	1.1	1	1.1	0.2	0.2	0.2	0.2	0.3	0.3	0.7	0.5	0.3	0.2	0.9	-0.3	1.8	1	0.2	-0.3	1.1	2.9	1.9	1.1	0.4	0.4	1.4	3

4 Supplementary Table 4

ID	variable	Relation with COVID-19	Indicator	Mechanism
M24	Albumin	- Biomarker on admission for risk of death (Tian et al., 2020)	- Decreased levels of albumin (−3.7 g/L, 95% CI, −5.3 to −2.1; P < .00001) (Tian et al., 2020)	
		-Decreased albumin level in the severe group (Ghahramani et al., 2020; Gong et al., 2020; Huang et al., 2020b; Youssef et al., 2020; Küçükceran et al., 2021; Liu et al., 2021a)	- Marked hypoalbuminemia occurred in 38.2%, 71.2%, and 82.4% patients in non-critically ill, critically ill, and death groups, respectively, on admission and 45.9%, 77.7%, and 95.6% of these three groups, respectively, during hospitalization. (Huang et al., 2020b)	
			- Compared to the mild patients, the level of albumin was lower in the severe patients (36 [33-38.5] vs 49.9 [37.4-43.6]; P < .0001) (Wan et al., 2020)	
O2	oxygen saturation	- Hypoxemia as predictor for patient is at risk of requiring admission to the intensive care unit (ICU) (Xie et al., 2020a)	- A concomitant decrease in SpO2, median in the Covid-19 cohort dropped below 93% one day prior to the event (Pimentel et al., 2020)	-Arterial hypoxemia early in SARS-CoV-2 infection is primarily caused by V/Q mismatch and thus persistence of pulmonary arterial blood flow to non-ventilated alveoli, reflected by a marked increase in P(A-a) O2 gradient (Gattinoni et al., 2020)
		- Hypoxemia has such an impact on prognosis and timely treatment decisions (Dhont et al., 2020)		The persistence of high pulmonary blood flow to non-aerated lung alveoli appears to be caused by the relative failure of the hypoxic pulmonary vasoconstriction mechanism (constriction of small intrapulmonary
		- Significantly correlated with death events identified using bivariate logistic regression (Pan et al., 2020a)		

		- Oxygen saturation among the most important predictors (Incerti et al., 2021)<br>- Low oxygen saturation in ARDS course of severe and critical COVID-19 types (Ghahramani et al., 2020)<br>- SpO2 and respiration rate were consistently selected as predictive features across outcomes and modeling methods (Wang et al., 2021a)<br>- Patients of ICU Group had lower oxygen saturation (Carlino et al., 2020)		arteries in response to alveolar hypoxia) during SARS-CoV-2 infection (Lang et al., 2020)
O3	PAO_2FIO_2 arterial oxygen pressure/ inspired fraction of oxygen	- Oxygen saturation to fraction of inspired oxygen ratio (SpO2/FiO2) as potential predictor of poor outcome for COVID-19 (Lu et al., 2020)<br>-Difference in PaO2/FiO2 ratio between survivors and non-survivors indicates this is associated with the severity of illness and thus prognosis (Yang et al., 2020)	- A strong and significant association between the square root SpO2/FiO2 value and the risk for death, with a unit decrease in the marker corresponding to 1.82-fold increase in the mortality risk (95% CI: 1.56–2.13) (Lu et al., 2020)	
DM1	Estimated glomerular filtration rate (eGFR)	- Reduction of eGFR may be an indicator of disease severity in COVID-19 (Pelayo et al., 2020) (Ouyang et al., 2020)<br>- Lower eGFRrate was associated with an increased hazard of death (Berenguer et al., 2020)	- Nearly half (49.3%) of the patients with COVID-19 had AKI. Patients with AKI had a significantly lower baseline estimated glomerular filtration rate (eGFR) and higher FiO2 requirement and D-dimer levels on admission (Pelayo et al., 2020)	-Heart-kidney crosstalk could be contributory to this observation, as cardiomyopathy can lead to renal congestion, hypotension, and renal hypoperfusion leading to a reduction in GFR. The myocardial dysfunction can possibly be attributed to hypoxia, thrombotic events, direct viral damage, and cytokine storm in critically ill patients with severe COVID-19 (Pelayo et al., 2020)

H3	Lymphocytes	- Lymphopenia indicator of propensity to ICU (Huang et al., 2020a; Wang et al., 2020a)	-Mean (IQR) ICU patients: 0.8 (0.5-0.9) x109/L (p= 0.03)	
		- ↑Lymphopenia prevalence in Non-Survivor patients. (p= 0.0001) (Zhou et al., 2020a)	Mean (IQR) Non-ICU patients: 0.9 (0.6-1.2) x109/L (p= 0.03)	
		- Lymphopenia high prevalence in patients with ARDS. (p= <0.001) (Wu et al., 2020a)	(Wang et al., 2020a)	
		- Significantly correlated with death events identified using bivariate logistic regression (Pan et al., 2020a)	-Mean (IQR) ICU patients: 0.4 (0.2–0.8) x109/L (p= 0.0041)	
		-Lower levels of lymphocytes count than mild severe patients ($P < 0.05$) (Ren et al., 2020)	Mean (IQR) Non-ICU patients: 1.0 (0.7–1.1) x109/L (p= 0.0041) (Huang et al., 2020a)	For patients with ARDS who died, lymphocyte counts (difference, −0.23 ×109/L; 95% CI, −0.41 to −0.07 ×109/L; P = .004) and CD8 T cells (difference, −134 cells/μL; 95% CI, −221 to −10 cells/μL; P = .05) were significantly decreased compared with patients with ARDS who survived. (Wu et al., 2020a)
			-Mean (IQR) of Non-Survivor patients: 0.6 (0.5–0.8) x109/L	
			(p= 0.0001). Mean (IQR) of Survivor patients: 1.1 (0.8–1.5) x109/L (p= 0.0001) (Zhou et al., 2020a)	
			- Lymphopenia (lymphocyte count, 0.8 × 109/L [interquartile range {IQR}, 0.6-1.1]) occurred in 97 patients (70.3%) (Wang et al., 2020a)	
			-Mean (IQR) of patients without ARDS 1.08 (0.72-1.45) x109/L. Mean (IQR) of patients with ARDS 0.67 (0.49-0.99) x109/L.	
			(p= <0.001) (Wu et al., 2020a)	
			- Patients requiring hospital admission also had a higher incidence of bilateral	

			infiltrates on chest radiographs (182 patients [54.7%] vs 5 patients [8.5%]), more pronounced lymphopenia (median lymphocyte count, 0.8 cells/µL [IQR, 0.6-1.1 cells/µL] vs 1.0 cells/µL [IQR, 0.7-1.6 cells/µL] (Suleyman et al., 2020)	
			- Lymphocyte counts of the severe patients (median = 0.8 × 109/L) were significantly lower than that of the mild patients (median = 1.2 × 109/L) (Wan et al., 2020)	
			- Lymphopenia was common in 75.4% of the patients (Zhang et al., 2020c)	
CT9	thoracic subcutaneous adipose tissue	- Subcutaneous adipose tissue attenuation do not progress with the severity in COVID-19 (Iacobellis et al., 2020a)		
DM7	TyG-BMI index	- Risk factor (Albashir, 2020)	- Overweight patients were associated with an 86% higher risk, and obesity with a 142% higher risk, of developing severe pneumonia compared with normal weight patients (Albashir, 2020)	The highest BMI was seen more often in serious cases and non-survivors. (Peng et al., 2020)
DB1	body mass index (BMI)	- BMI >40 kg/m2 was the second strongest independent predictor of hospitalization (Bhasin et al., 2020; Petrilli et al., 2020)	- Random-effects dose-response meta-analysis showed a linear association between BMI and both severe COVID-19 and mortality (Du et al., 2021)	
		- BMI was associated strongly with risk of death related to COVID-19(Sattar et al., 2020)		
		- Obesity (BMI > 30 kg/m2) was associated with a significantly increased risk of critical COVID-19 and mortality (Du et al., 2021)		
B0	weight	- Associated with BMI		

		(ICNARC – Intensive Care National Audit & Research Centre)		
CT13	presence of steatosis on tomography	- The liver tissue showed moderate microvesicular steatosis and mild lobular activity, but there was no conclusive evidence to support SARS-CoV-2 infection or drug-induced liver injury as the cause (Xu et al., 2020).<br>-Presence of fatty liver is a strong predictor for severe disease. (Medeiros et al., 2020; Parlak et al., 2021)		Novel coronavirus may produce, in some cases, relevant hepatic damage, probably through the immune interactions requiring the action of intrahepatic cytotoxic T cells and Kupffer cell (Zhang et al., 2020c). Direct cytotoxicity due to active viral replication in hepatic cells: SARS-CoV-2 binds to target cells through ACE2. Because ACE2 is expressed abundantly in the liver and in particular on biliary epithelial cells, the liver is a potential target for direct infection (Amin, 2021)
HR	heart rate	- Cardiac arrhythmia (Kang et al., 2020)<br>- Patients of ICU group had higher heart rate (Carlino et al., 2020)	- ≥125 beats per min, was present in 4% of Non-Survivor patients. (p=0.024) (Zhou et al., 2020a)	
T0	axillary temperature	- Main clinical symptom of COVID-19 patients - ↑ (Rod et al., 2020)	- Fever in 88.5% of COVID-19 patients.	
		- Higher temperature was related to severe 2019 novel coronavirus pneumonia and composite endpoint. (R1 = 0.362, R2 = 0.383, P3<0.001, P4<0.001) (Zhang et al., 2020b)	Body temperature is a risk factor with a high consistency as predictor for COVID-19 severity (Rod et al., 2020)	
M36	Prothrombin	- ↑ indicator of propensity to ICU. (p= 0.012) (Huang et al., 2020a)	- Mean (IQR) ICU patients: 12.2 (11.2-13.4) s (p= 0.012)	For patients with ARDS who died, coagulation function indices (D-dimer [difference, 2.10 μg/mL; 95% CI, 0.89-5.27 μg/mL; P = .001]) were significantly elevated compared with
		- ↑ time, in Non-Survivor patients (p= 0.0004) (Zhou et al., 2020a)	Mean (IQR) Non-ICU patients: 10.7 (9.8-12.1) s (p= 0.012) (Huang et al., 2020a)	

		- Coagulation indexes of all of the patients were nearly in the normal range, the Pt, APTT, and d-dimer of the severe patients were higher than those of the mild patients (Wan et al., 2020)	- Mean (IQR) of Non-Survivor patients: 12.1 (11.2-13.7) s	patients with ARDS who survived. (Wu et al., 2020a)
			(p= 0.0004). Mean (IQR) of Survivor patients: 11.4 (10.4-12.6) s (p= 0.0004) (Zhou et al., 2020a)	
			- Mean (IQR) of Non-Survivor patients: 15.5 (14.4-16.3) s (p= <0.001). Mean (IQR) of Survivor patients: 13.6 (13.0-14.3) s (p= <0.001) (Tang et al., 2020)	
			- Baseline characteristics of the first 99 patients hospitalized in Wuhan found that 5% had elevated prothrombin (PT). (Chen et al., 2020b)	
			- Prolonged prothrombin time (13.0 seconds [IQR, 12.3-13.7]) in 80 patients (58%) (Wang et al., 2020a)	
			- Prolonged PT in patients at high risk of severe COVID-19 (Elshazli et al., 2020; Ghahramani et al., 2020; Liao et al., 2020; Liu et al., 2021a)	
M33	vitamin D	- Association between vitamin D deficiency and poor clinical outcome (Raharusun, 2020; Munshi et al., 2021) (Hastie et al., 2020; Radujkovic et al., 2020)		Vitamin D is a key regulator of the renin-angiotensin system that is exploited by SARS-CoV-2 for entry into the host cells. Reduces concentration of pro- inflammatory cytokines and increases levels of anti-inflammatory cytokines, enhances the production of natural antimicrobial peptide and activates defensive cells such as macrophages that could destroy SARS-CoV-2 (Kumar et al., 2021)

B1	Height	- Associated with BMI (ICNARC – Intensive Care National Audit & Research Centre)		
H8	Hemoglobin	- The severity of disease and prognosis of patients with COVID-19 might depend on lower hemoglobin levels as severe cases had significantly lower hemoglobin levels than moderate cases. (Taneri et al., 2020)	- Severe COVID-19 cases had lower hemoglobin [weighted mean difference (WMD), − 4.08 g/L (95% CI − 5.12; − 3.05)] and red blood cell count [WMD, − 0.16 × 1012 /L (95% CI − 0.31; − 0.014)], and higher ferritin [WMD, − 473.25 ng/mL (95% CI 382.52; 563.98)] and red cell distribution width [WMD, 1.82% (95% CI 0.10; 3.55)] (Taneri et al., 2020).	Low hemoglobin in COVID-19 patients, especially on populations at risk of complications and mortality, could indicate that the patients could suffer from a decreased capability of hemoglobin to support the increased peripheral tissue demands for oxygen due to the hyper-metabolic states during infection (Taneri et al., 2020)
			- In severe COVID-19 hematocrit (HCT) was significantly higher in patients with CVD than in non-CVD patients (P=0.007) (Xie et al., 2020c)	
P1	diastolic blood pressure (DBP)			
DP1	Mean Arterial Pressure (MAN)			
P0	systolic blood pressure (SBP)	- Severe patients had significantly higher value of respiratory rate, systemic blood pressure (SBP), and mortality and/or more likely to receive auxiliary ventilation, and invasive mechanical ventilation (all $P < 0.05$)(Ren et al., 2020)	- Arterial hypertension is risk of death in patients odds ratio [OR], 2.5; 95% confidence interval [CI], 2.1-3.1; P < .00001)	
		- Pearson correlations showed that hypertension and systolic blood pressure (SBP) were associated with death and	Coronary heart disease is risk of death in patients odds ratio (OR, 3.8; 95% CI, 2.1-6.9; P < .00001) (Tian et al., 2020)	

		respiratory distress parameters. SBP but not hypertension was a covariate in both mortality and survival prediction models. SBP was elevated in deceased compared with discharged COVID-19 patients.(Caillon et al., 2021)	- Significant predictors of heart failure were average systolic blood pressure (SBP) (hazard ratio (HR) per 10 mmHg 1.89, 95% confidence interval (CI): 1.15, 3.13) and pulse pressure (HR per 10 mmHg 2.71, 95% CI: 1.39, 5.29). The standard deviations of SBP and diastolic BP were independently associated with mortality and ICU admission. (Ran et al., 2020)	
DP0	pulse pressure (PP)	- Hypertension is a risk factor (Sardu et al., 2020)		Hypertension (Sardu et al., 2020)
		- Risk of death in patients with COVID-19 infection (Sardu et al., 2020).		
H15	Cytopenia	- High prevalence in Non-ICU patients. (p= <0.041) (Huang et al., 2020a)	- White blood cell count less than 4x109/L	Available data of 33 COVID-19 patients who either recovered or died. Characteristics of the 5 nonsurvivors compared with the 28 survivors included rising D-dimer, progressive lymphopenia, and renal dysfunction. (Wang et al., 2020a)
		- High prevalence in survivor patients (p= <0.0001) (Zhou et al., 2020a)	8% of ICU patients	
		- High prevalence in patients without ARDS (p= <0.001) (Wu et al., 2020a)	33% of Non-ICU patients	
			(p= <0.041)	
			-White blood cell count less than 4x109/L	
			9% of Non-Survivor patients	
			20% of Survivor patients	
			(p= <0.0001) (Zhou et al., 2020a)	
			- White blood cell count. Mean (IQR) of patients without ARDS 5.02 (3.37-7.18) x109/L. Mean (IQR) of patients with	

			ARDS 8.32 (5.07-11.20) x109/L. (p= <0.001) (Wu et al., 2020a)	
M10	Creatinine	- ↑ indicator of propensity to ICU. (p= <0.04) (Wang et al., 2020a)	- Mean (IQR) ICU patients: 80 (66-106) µmol/L (p= <0.04)	
		- Severe group had higher levels of creatinine (Ghahramani et al., 2020; Iavarone et al., 2020; Mudatsir et al., 2020; Shao et al., 2020; Malik et al., 2021)	- Mean (IQR) Non-ICU patients: 71 (58-84) µmol/L (p= <0.04) (Wang et al., 2020a)	
			- Severe group had higher levels of aspartate aminotransferase (AST), lactate dehydrogenase (LDH), creatinine, uric acid (UA), CRP, and FPG, compared with mild group (P < 0.05)(Ren et al., 2020)	
CT16	consolidation in tomography	- Main sign on CT imagen of COVID-19 lesion (Li and Xia, 2020)	- CT showed consolidation in 49 of the 51 (96.1%) patients	In COVID-19 patients, consolidation may relate to cellular fibromyxoid exudates in alveoli (Xu et al., 2020)
		- This manifestation could serve as an alert in the management of patients (Larici et al., 2020)	CT showed increasing density of consolidation on the first and second follow-up studies, which indicates marked disease progression, and showed findings suggestive of improvement on the third and fourth follow-up studies. Occurrence rate of 2~64% (Li and Xia, 2020)	
			- The majority (134, 99.3%) had abnormal results, showing typical images that were bilateral multiple ground-glass opacities or consolidation (Zhang et al., 2020c)	
CT17	presence of grounded glass (GG) and consolidation		- Enlargement (greater than 3 mm in diameter) of subsegmental pulmonary vessels within the areas of GG has also been described in the early phase of	

	(0=no, 1=yes) (Larici et al., 2020)		COVID-19 pneumonia in up to 89 % of cases. (Larici et al., 2020)	
			(Grassi et al., 2020; Pan et al., 2020b)	
H14	platelets		- Mild thrombocytopenia has been detected in 58–95% of severe cases of COVID-19 (Chen et al., 2020b; Cheng et al., 2020a; He et al., 2020; Hu et al., 2020; Suleyman et al., 2020; Wan et al., 2020; Wu et al., 2020a; Yang et al., 2020; Abrishami et al., 2021; Kim et al., 2021; Wool and Miller, 2021)	Lung injury (Wool and Miller, 2021)
symD	days with symptoms before arrival to hospital	- The mean time from symptom onset to hospitalization overall is 5.74 days, which is slightly longer as compared to the reported delay in other countries, but depending on the patient population, estimates range between 3 and 10.4 days (Faes et al., 2020)	- The time from symptom onset to hospitalization is largest in the working age population (20–60 years), followed by the elderly (60–80) years. (Faes et al., 2020)	
M21	indirect bilirubin	- Increased serum bilirubin level was observed in fatal cases (Wu et al., 2020b)		- Risk factor of coronary heart disease
				(Deng et al., 2020; Ouyang et al., 2020; Wu et al., 2020a; Lopes et al., 2021; Sun et al., 2021)
M20	direct bilirubin	- Higher direct bilirubin levels is associated with severe Covid-19 (Gong et al., 2020; Liang et al., 2020)	- Patients with severe Covid-19 displayed higher bilirubin levels compared to those with milder forms (mean difference ranging between 0.27 and 0.95 μmol/L) (Paliogiannis et al., 2020)	
			- Bilirubin concentration was significantly higher in patients with severe Covid-19 (SMD: 0.48 μmol/L;	

			95% CI, 0.11 to 0.85 µmol/L, P = .012) (Paliogiannis and Zinellu, 2020)	
CT4	average epicardial fat	- Its elevation increases mortality in COVID-19 (Deng et al., 2020; Iacobellis et al., 2020b, 2020a; Kim and Han, 2020; Malavazos et al., 2020; Zhao, 2020; Abrishami et al., 2021; Bihan et al., 2021; Grodecki et al., 2021; Slipczuk et al., 2021)		Is associated with extent of pneumonia and adverse outcomes (Grodecki et al., 2021)
M26	creatinine kinase	- Elevated during COVID-19, it is a nonspecific marker of muscle damage (Chen et al., 2020b; Deng et al., 2020, 2020; Henry et al., 2020; Huang et al., 2020b; Izcovich et al., 2020; Liu et al., 2020; Mudatsir et al., 2020; Qiu et al., 2020; Rodriguez-Morales et al., 2020; Suleyman et al., 2020; Wang et al., 2020a; Zhang et al., 2020d; Abrishami et al., 2021; Malik et al., 2021)	- Creatine kinase, U/L were 39·0 (19·5–151·0) in non survivors patients versus 18·0 (12·5–52·1) survivors patients $p<0.001$(Zhou et al., 2020a)	
M23	alanine transaminase (ALT)	- Elevated in COVID (Zhang et al., 2020b)<br>- High values of the pyridoxal phosphate-dependent enzymes AST and ALT were significantly associated with the COVID-19 disease (Ferrari et al., 2020; Ou et al., 2020)<br>- Related to severe coronavirus pneumonia and composite endpoint (Zhang et al., 2020b)<br>-Increase alanine aminotransferase (ALT) in the severe group compared with the non-severe group. (Ghahramani et al., 2020; Huang et al., 2020b; Mudatsir et al.,		

		2020; Youssef et al., 2020; Liu et al., 2021a; Malik et al., 2021)		
M22	aspartate aminotransferase (AST)	- ↑ indicator of propensity to ICU (p= <0.001) (Wang et al., 2020a)	- Mean (IQR) ICU patients: 53 (30-70) U/L (p= <0.001)	For patients with ARDS who died, the value of liver damage indices (total bilirubin [difference, 2.60 μM; 95% CI, 0.30-5.20 μM; P = .03]), were significantly elevated compared with patients with ARDS who survived (Wu et al., 2020a)
		- High levels associated with patients among ARDS. (p= <0.001) (Wu et al., 2020a)	Mean (IQR) Non-ICU patients: 29 (21-38) U/L (p= <0.001) (Wang et al., 2020a)	
		-Increase levels of aspartate aminotransferase (AST) in the severe group compared with the non-severe group. (Ghahramani et al., 2020; Huang et al., 2020b; Lei et al., 2020; Mudatsir et al., 2020; Youssef et al., 2020; Malik et al., 2021; Zhao et al., 2021)	- Mean (IQR) of patients without ARDS 30.0 (24.0-38.50) U/L. Mean (IQR) of patients with ARDS 38.0 (30.5-53.0) U/L. (p= <0.001) (Wu et al., 2020a)	
			-Severe group had higher levels of aspartate aminotransferase (AST), lactate dehydrogenase compared with mild group (P < 0.05) (Ren et al., 2020)	
M34	Ferritin	- Biomarkers, such high serum procalcitonin and ferritin have also emerged as poor prognostic factors in COVID-19 patients (Terpos et al., 2020) (Rostami and Mansouritorghabeh, 2020)	- Indicator of thrombotic complication, mean (min-max) 1182 (697-2081) µg/L (P=0.0020) (Al-Samkari et al., 2020).	-Levels of serum ferritin, d-dimer, lactate dehydrogenase, and IL-6 are increased during the worsening of the disease, providing an indication of the risk of mortality (Zhou et al., 2020a)
			-Severe patients and discharged patients have greater proportions of increased level of ferritin than non-severe patients and hospitalized patients (100% vs. 50%, 92.3% vs. 37.9% respectively, P < .001) and suggested that serum ferritin is a potential risk factor of poor prognosis in COVID-19 patients. (Sun et al., 2020).	-Hyperferritinemia caused by the excessive inflammation due to the infection is associated with the admission to the intensive care unit and high mortality, and represents an indication to recognize high-risk patients to guide the therapeutic intervention to control inflammation (Cheng et al., 2020b)
M25	lactic dehydrogenase	- Severe group had higher levels of lactate dehydrogenase (LDH), compared with mild group (P < 0.05) (Ren et al., 2020)	- Related to severe coronavirus pneumonia and composite endpoint (Zhang et al., 2020b)	

		- Increased lactic dehydrogenase in the severe group (Ghahramani et al., 2020; Gong et al., 2020; Ouyang et al., 2020; Zhou et al., 2020a)	were significantly correlated with death events identified using bivariate logistic regression (Pan et al., 2020a)	
			- Elevated lactate dehydrogenase (261 U/L [IQR, 182-403]) in 55 patients (39.9%) (Wang et al., 2020a)	
BR	breathing rate	- Significantly correlated with death events identified using bivariate logistic regression (Pan et al., 2020a)	- Multivariate regression showed increasing odds ratio (OR) of in-hospital death associated with respiratory rates >20 breaths/min (OR: 5.14, 95% CI: 1.19-22.15, p = 0.028) (Vahedian-Azimi et al., 2021)	Chest pain (Andrikopoulou et al., 2020 in pregnant women)
		- SpO2 and respiration rate were consistently selected as predictive features across outcomes and modeling methods (Wang et al., 2021a)	- Severe patients had significantly higher value of respiratory rate, systemic blood pressure (SBP), and mortality and/or more likely to receive auxiliary ventilation, and invasive mechanical ventilation (all $P < 0.05$)(Ren et al., 2020)	
		- >24 breaths per minute is associated with severe cases in COVID-19 (Huang et al., 2020b; Pan et al., 2020a; Ren et al., 2020; Suleyman et al., 2020; Wan et al., 2020; Zhang et al., 2020c)		(Andrikopoulou et al., 2020; Huang et al., 2020b; Pan et al., 2020a; Ren et al., 2020; Suleyman et al., 2020; Wan et al., 2020; Wang et al., 2020a; Zhang et al., 2020b, 2020a)
M37	Fibrinogen	- Increased during COVID-19 (Connors and Levy, 2020) (Ghahramani et al., 2020; Ouyang et al., 2020)	- Fibrinogen levels in all patients were elevated on admission.	-The coagulopathy with COVID-19 is a result of the inflammatory response to SARS-CoV-2 infection resulting in thrombo inflammation and driving thrombosis.
		- Decreased in disseminated intravascular coagulation (Connors and Levy, 2020)	- Nonsurvivors had evidence of progressive disseminated intravascular coagulation with decreased fibrinogen (Connors and Levy, 2020)	-Coagulopathy is manifest as elevated fibrinogen, elevated D-dimers, and minimal change in PT, aPTT, and platelet count in early stages of infection (Ranucci et al., 2020)
				-IL-6 is a powerful pro-inflammatory cytokine, which induces tissue factor

				gene expression in endothelial cells and monocytes, fibrinogen synthesis, and platelet production, without affecting fibrinolysis.
				(Xie et al., 2020b)
M13	C-reactive protein (PCR)	- Elevated hs-CRP can effectively triage suspected COVID-19 patients (Li et al., 2020; Mardani et al., 2020)	- CRP level at admission represent a simple and independent factor that can be useful for early detection of severity during COVID-19 (Ahnach et al., 2020)	Hypercoagulability is due to the profound derangement of hemostasis and is the likely contributor to pulmonary embolism and/or deep vein thrombosis of the lower limbs observed in patients with COVID-19 (Panigada et al., 2020).
		-Higher concentrations of C-reactive protein was associated with an increased hazard of death (Berenguer et al., 2020) (Gong et al., 2020)	-Significantly correlated with death events identified using bivariate logistic regression (Pan et al., 2020a)	The increase in inflammation markers and acute phase reactants are linked with the underlying systemic vasculitic processes and the cytokine storm that cause most parenchymal lesions in vital organs (Ahmed and Ghani, 2020).
		- CRP was positively correlated to the severity of COVID-19 pneumonia (Chen et al., 2020c)	- Severe group had higher levels of aspartate aminotransferase (AST), lactate dehydrogenase (LDH), creatinine, uric acid (UA), CRP, and FPG, compared with mild group ($P < 0.05$) (Ren et al., 2020)	
		- Indexes related to myocardial injury, such as creatine kinase, glutamic oxaloacetylase, LDH, and C-reactive protein (CRP), increased more significantly in the severe patients (Wan et al., 2020)	- Higher concentration of C-reactive protein (91.9%) in severe patients vs non severe ones ($p<0.001$) (Zhang et al., 2020c)	
DH6				

	neutrophils-lymphocytes relation (NLR)	- NLR can serve as a predictor of COVID-19 disease severity (Wang et al., 2021b) (Fu et al., 2020; Ghahramani et al., 2020; Lagunas-Rangel, 2020; Liang et al., 2020; Ouyang et al., 2020; Ok et al., 2021)		A statistically significant positive correlation is present between PCT and NLR in the severe group at the first week of admission (Wang et al., 2021b)
H0	Leukocytes	- Related to severe coronavirus pneumonia and composite endpoint (Zhang et al., 2020b)		
		- Severe patients had higher values (Ren et al., 2020) (Ok et al., 2021)		
H1	total neutrophils	- Related to severe coronavirus pneumonia and composite endpoint (Zhang et al., 2020b)	- Increased in COVID-19, it is found in current infection (Deng et al., 2020; Huang et al., 2020b; Hu et al., 2020; Qin et al., 2020; Shi et al., 2020; Wan et al., 2020; Wu et al., 2020a; Zhou et al., 2020a; Abrishami et al., 2021)	
		- Significantly correlated with death events identified using bivariate logistic regression (Pan et al., 2020a)		
		- Severe patients had higher values (Ren et al., 2020) (Elshazli et al., 2020; Izcovich et al., 2020; Ouyang et al., 2020; Ok et al., 2021)		
M1	triglycerides	- Risk factor for atherosclerotic cardiovascular disease, elevated during COVID-19, excessive free fatty acids release		It is related to atherosclerotic cardiovascular disease, acute pancreatitis (Peng et al., 2020)
		(Aung et al., 2020; Deng et al., 2020; Huang et al., 2020b; Prilutskiy et al., 2020; Ren et al., 2020; Wang et al., 2020b; Webb et al., 2020; Saand et al., 2021)		

DM6	TyG index	- TyG index was significantly associated with an increased risk of severe case and mortality (Ren et al., 2020).	- TyG index levels were significantly higher in the severe cases and death group (mild vs. severe 8.7 ± 0.6 vs. 9.2 ± 0.6, P < 0.001; survivor vs. deceased 8.8 ± 0.6 vs. 9.3 ± 0.7, P < 0.001), respectively (Ren et al., 2020).	
			- TyG index, which calculated by triglycerides and glucose, was markedly higher in severe cases than in mild ones (P < 0.05) (Ren et al., 2020).	
M5	glucose	-Increased glucose level in the severe group compared with the non-severe group (Ghahramani et al., 2020)	- Severe COVID-19 was associated with higher blood glucose (WMD 2.21, 95% CI: 1.30-3.13, P < 0.001) (Chen et al., 2020a)	Hyperinsulinaemia, hyperglycaemia and hypertension increase inflammation, coagulation and thrombosis risk (Cooper et al., 2020)
			- Glucose was significantly increased (P < 0.05) in non-survivors compared with survivors (Ouyang et al., 2020)	
M8	BUN	- It can be used to predict renal function, higher in severe cases (Chen et al., 2020b; Cheng et al., 2020a; Ghahramani et al., 2020; Gong et al., 2020; Henry et al., 2020; Izcovich et al., 2020; Liu et al., 2020; Mudatsir et al., 2020; Qiu et al., 2020; Webb et al., 2020; Abrishami et al., 2021; Küçükceran et al., 2021; Ok et al., 2021; Zhao et al., 2021)	- Multivariate regression showed increasing odds ratio (OR) of in-hospital death associated with blood urea nitrogen (BUN) >19 mg/dL (OR: 4.54, 95% CI: 1.30-15.85, p = 0.017) (Vahedian-Azimi et al., 2021)	It is a marker for predicting persistent organ failure after 48 h of hospital admission; associated to renal failure, heart failure, pulmonary embolism, acute pancreatitis, ventilation failure, diabetes mellitus
				(Cheng et al., 2020a; Ghahramani et al., 2020; Qiu et al., 2020; Abrishami et al., 2021)
DM4	BUN to creatinine ratio	- Blood urea nitrogen-to-creatinine ratio (BCR) is a predictor for mortality in patients with COVID-19. (Liu et al., 2021b)	- In multivariate Cox proportional hazard model BUN/Cr ratio (hazard ratio [HR] = 1.02; 95% CI: 1.01-1.05; P = .030) was independent predictors for survival of COVID-19 disease. (Ok et al., 2021)	

M35	dimer D	- Were significantly correlated with death events identified using bivariate logistic regression (Pan et al., 2020a)	- A biomarker of fibrin formation and degradation. Coagulopathy disorder indicator. It highly correlates with a poor prognosis when increased (Chen et al., 2020b; Cheng et al., 2020a; Connors and Levy, 2020; Deng et al., 2020; Gao et al., 2020; He et al., 2020; Huang et al., 2020a; Qiu et al., 2020; Suleyman et al., 2020; Wan et al., 2020; Zhang et al., 2020c; Lenka et al., 2021)	It is correlated with venous thromboembolism, cardiac injury and sustained inflammatory response (Breakey and Escher, 2020; Cheng et al., 2020a; Deng et al., 2020; Lippi and Plebani, 2020; Zhang et al., 2020c)
		- Coagulation indexes of all of the patients were nearly in the normal range, the Pt, APTT, and d-dimer of the severe patients were higher than those of the mild patients (Wan et al., 2020)		
		- Increase of D-dimer in severe Covid-19 group (Ghahramani et al., 2020; Ouyang et al., 2020; Zhou et al., 2020a; Abderrahim et al., 2021)		
M29	Ultrasensitive troponin	- Elevated troponin is an independent predictor of 30-day mortality (García de Guadiana-Romualdo et al., 2021)	- Sex-specific elevated troponin levels were significantly associated with 30-day mortality, with adjusted odds ratios (ORs) of 3.00 for total population, 3.20 for cardiac troponin T and 3.69 for cardiac troponin I.(García de Guadiana-Romualdo et al., 2021)	
		- Associated to a worse prognosis in hospitalized patients with severe COVD-19 (Gómez-Mesa et al., 2021)	- Hypersensitive cardiac troponin I(hs-cTnI) > in COVID-10 patients (Zheng et al., 2020)	
		- Higher levels were observed in death events group (Pan et al., 2020a) (Mudatsir et al., 2020)		
D1	age	- Aging is an important risk factor for severe COVID-19 disease and its adverse health outcomes including hospitalization, ICU admission, and death (Wu et al., 2020a)	- Case fatality ratio (CFR) of COVID-19 increases with age, from 0.4 % or lower in patients aged in the 40s or younger, 1.3 % among those in their 50s, 3.6 % in their 60s, 8% in their 70s, to 14.8 % in	An immune hypothesis for COVID-19 vulnerability of older adults. It involves age-related impairment of immune defense against SARS-CoV-2 infection, or immunosenescence,

			their 80s or older; the overall CFR is 2.3 %(Wu et al., 2020a; Zhu et al., 2020)	and increased risk for immunopathology (Sardu et al., 2020)
		- The risk of fatal or critical care unit-treated COVID-19 increased with age(McGurnaghan et al., 2021)	-Non-survivors tend to be older (median age 80 vs. 64) (Bertsimas et al., 2020)	
		- Age was the most important predictor of all-cause mortality (Incerti et al., 2021)	- Increasing odds of in-hospital death associated with older age (odds ratio 1·10, 95% CI 1·03–1·17, per year increase; p=0·0043) (Zhou et al., 2020a)	
		- Elder age was an independent risk factors for death of severe patients (Zhang et al., 2021)	- Compared to mild patients, severe patients were significantly older (median age 56 years [IQR, 52-73] vs 44 years [IQR, 33-49]; P < .001) (Wan et al., 2020)	
			-Log-linear relationship between age and risk of death continues into older age groups(O'Driscoll et al., 2021)	
CT6	right main artery diameter	No items found.		
CT7	left main artery diameter	No items found.		
CT5	main trunk pulmonary artery diameter	-Increased main pulmonary artery diameter is associated with poorer prognosis for patients with COVID-19 pneumonia.(Grassi et al., 2020; Raoufi et al., 2020; Erdoğan et al., 2021; Esposito et al., 2021; Jalde et al., 2021; Yildiz et al., 2021)	- Increased in 8/126 (6.3%) patients. (Grassi et al., 2020)	Discrete pulmonary nodules, increased trunk diameter of the pulmonary artery, pleural effusion can be found but in a low non-significant percentage of cases (7.9%, 6.3%, 14.3%, respectively) (Grassi et al., 2020)

Abderrahim, E. K., Manal, M., Ghizlane, E. A., Mohammed, A. a. B. D. I., Abdelilah, E., Mohammed, M., et al. (2021). Predictive factors of mortality related to COVID-19: A retrospective cohort study of 600 cases in the intensive care unit of the university hospital of Oujda. Ann Med Surg (Lond) 69, 102711. doi:10.1016/j.amsu.2021.102711.
Abrishami, A., Eslami, V., Baharvand, Z., Khalili, N., Saghamanesh, S., Zarei, E., et al. (2021). Epicardial adipose tissue, inflammatory biomarkers and COVID-19: Is there a possible relationship? International

Immunopharmacology 90, 107174. doi:10.1016/j.intimp.2020.107174.
Ahmed, S., and Ghani, F. (2020). Trend analysis of lab tests requisitions of COVID-19 prognostic biomarkers at a clinical chemistry reference laboratory-an observational study. Annals of Medicine and Surgery 60, 522–525. doi:10.1016/j.amsu.2020.10.067.
Ahnach, M., Zbiri, S., Nejjari, S., Ousti, F., and Elkettani, C. (2020). C-reactive protein as an early predictor of COVID-19 severity. J Med Biochem 39, 500–507. doi:10.5937/jomb0-27554.
Al-Samkari, H., Karp Leaf, R. S., Dzik, W. H., Carlson, J. C. T., Fogerty, A. E., Waheed, A., et al. (2020). COVID-19 and coagulation: bleeding and thrombotic manifestations of SARS-CoV-2 infection. Blood 136, 489–500. doi:10.1182/blood.2020006520.
Albashir, A. A. D. (2020). The potential impacts of obesity on COVID-19. Clin Med (Lond) 20, e109–e113. doi:10.7861/clinmed.2020-0239.
Amin, M. (2021). COVID-19 and the liver: overview. European Journal of Gastroenterology & Hepatology 33, 309–311. doi:10.1097/MEG.0000000000001808.
Andrikopoulou, M., Madden, N., Wen, T., Aubey, J. J., Aziz, A., Baptiste, C. D., et al. (2020). Symptoms and Critical Illness Among Obstetric Patients With Coronavirus Disease 2019 (COVID-19) Infection. Obstetrics & Gynecology 136, 291–299. doi:10.1097/AOG.0000000000003996.
Aung, N., Khanji, M. Y., Munroe, P. B., and Petersen, S. E. (2020). Causal Inference for Genetic Obesity, Cardiometabolic Profile and COVID-19 Susceptibility: A Mendelian Randomization Study. Front. Genet. 11. doi:10.3389/fgene.2020.586308.
Berenguer, J., Ryan, P., Rodríguez-Baño, J., Jarrín, I., Carratalà, J., Pachón, J., et al. (2020). Characteristics and predictors of death among 4035 consecutively hospitalized patients with COVID-19 in Spain. Clin Microbiol Infect 26, 1525–1536. doi:10.1016/j.cmi.2020.07.024.
Bertsimas, D., Lukin, G., Mingardi, L., Nohadani, O., Orfanoudaki, A., Stellato, B., et al. (2020). COVID-19 mortality risk assessment: An international multi-center study. PLoS One 15, e0243262. doi:10.1371/journal.pone.0243262.
Bhasin, A., Nam, H., Yeh, C., Lee, J., Liebovitz, D., and Achenbach, C. (2020). Is BMI Higher in Younger Patients with COVID-19? Association Between BMI and COVID-19 Hospitalization by Age. Obesity 28, 1811–1814. doi:https://doi.org/10.1002/oby.22947.
Bihan, H., Heidar, R., Beloeuvre, A., Allard, L., Ouedraogo, E., Tatulashvili, S., et al. (2021). Epicardial adipose tissue and severe Coronavirus Disease 19. Cardiovasc Diabetol 20, 147. doi:10.1186/s12933-021-01329-z.
Breakey, N., and Escher, R. (2020). D-dimer and mortality in COVID-19: a self-fulfilling prophecy or a pathophysiological clue? Swiss Medical Weekly 150. doi:10.4414/smw.2020.20293.
Caillon, A., Zhao, K., Klein, K. O., Greenwood, C. M. T., Lu, Z., Paradis, P., et al. (2021). High Systolic Blood Pressure at Hospital Admission Is an Important Risk Factor in Models Predicting Outcome of COVID-19 Patients. Am J Hypertens 34, 282–290. doi:10.1093/ajh/hpaa225.
Carlino, M. V., Valenti, N., Cesaro, F., Costanzo, A., Cristiano, G., Guarino, M., et al. (2020). Predictors of Intensive Care Unit admission in patients with coronavirus disease 2019 (COVID-19). Monaldi Arch Chest Dis 90. doi:10.4081/monaldi.2020.1410.
Chen, J., Wu, C., Wang, X., Yu, J., and Sun, Z. (2020a). The Impact of COVID-19 on Blood Glucose: A Systematic Review and Meta-Analysis. Front Endocrinol (Lausanne) 11, 574541. doi:10.3389/fendo.2020.574541.
Chen, N., Zhou, M., Dong, X., Qu, J., Gong, F., Han, Y., et al. (2020b). Epidemiological and clinical characteristics of 99 cases of 2019 novel coronavirus pneumonia in Wuhan, China: a descriptive study. The Lancet 395, 507–513. doi:10.1016/S0140-6736(20)30211-7.
Chen, W., Zheng, K. I., Liu, S., Yan, Z., Xu, C., and Qiao, Z. (2020c). Plasma CRP level is positively associated with the severity of COVID-19. Ann Clin Microbiol Antimicrob 19, 18. doi:10.1186/s12941-020-00362-2.
Cheng, A., Hu, L., Wang, Y., Huang, L., Zhao, L., Zhang, C., et al. (2020a). Diagnostic performance of initial blood urea nitrogen combined with D-dimer levels for predicting in-hospital mortality in COVID-19 patients. International Journal of Antimicrobial Agents 56, 106110. doi:10.1016/j.ijantimicag.2020.106110.
Cheng, L., Li, H., Li, L., Liu, C., Yan, S., Chen, H., et al. (2020b). Ferritin in the coronavirus disease 2019 (COVID-19): A systematic review and meta-analysis. J Clin Lab Anal 34. doi:10.1002/jcla.23618.
Connors, J. M., and Levy, J. H. (2020). COVID-19 and its implications for thrombosis and anticoagulation. Blood 135, 2033–2040. doi:10.1182/blood.2020006000.
Cooper, I. D., Crofts, C. A. P., DiNicolantonio, J. J., Malhotra, A., Elliott, B., Kyriakidou, Y., et al. (2020). Relationships between hyperinsulinaemia, magnesium, vitamin D, thrombosis and COVID-19: rationale for clinical management. Open Heart 7. doi:10.1136/openhrt-2020-001356.
Deng, M., Qi, Y., Deng, L., Wang, H., Xu, Y., Li, Z., et al. (2020). Obesity as a Potential Predictor of Disease Severity in Young COVID-19 Patients: A Retrospective Study. Obesity 28, 1815–1825. doi:https://doi.org/10.1002/oby.22943.
Dhont, S., Derom, E., Van Braeckel, E., Depuydt, P., and Lambrecht, B. N. (2020). The pathophysiology of “happy” hypoxemia in COVID-19. Respir Res 21, 198. doi:10.1186/s12931-020-01462-5.
Du, Y., Lv, Y., Zha, W., Zhou, N., and Hong, X. (2021). Association of body mass index (BMI) with critical COVID-19 and in-hospital mortality: A dose-response meta-analysis. Metabolism 117, 154373. doi:10.1016/j.metabol.2020.154373.
Elshazli, R. M., Toraih, E. A., Elgaml, A., El-Mowafy, M., El-Mesery, M., Amin, M. N., et al. (2020). Diagnostic and prognostic value of hematological and immunological markers in COVID-19 infection: A meta-analysis of 6320 patients. PLoS One 15, e0238160. doi:10.1371/journal.pone.0238160.
Erdoğan, M., Öztürk, S., Erdöl, M. A., Kasapkara, A., Beşler, M. S., Kayaaslan, B., et al. (2021). Prognostic utility of pulmonary artery and ascending aorta diameters derived from computed tomography in COVID-19 patients. Echocardiography. doi:10.1111/echo.15170.
Esposito, A., Palmisano, A., Toselli, M., Vignale, D., Cereda, A., Rancoita, P. M. V., et al. (2021). Chest CT-derived pulmonary artery enlargement at the admission predicts overall survival in COVID-19 patients: insight from 1461 consecutive patients in Italy. Eur Radiol 31, 4031–4041. doi:10.1007/s00330-020-07622-x.
Faes, C., Abrams, S., Van Beckhoven, D., Meyfroidt, G., Vlieghe, E., Hens, N., & Belgian Collaborative Group on COVID-19 Hospital Surveillance. (2020). Time between Symptom Onset, Hospitalisation and Recovery or Death: Statistical Analysis of Belgian COVID-19 Patients. International Journal of Environmental Research and Public Health, 17(20), 7560. https://doi.org/10.3390/ijerph17207560
Ferrari, D., Motta, A., Strollo, M., Banfi, G., and Locatelli, M. (2020). Routine blood tests as a potential diagnostic tool for COVID-19. Clin Chem Lab Med 58, 1095–1099. doi:10.1515/cclm-2020-0398.

Fu, J., Kong, J., Wang, W., Wu, M., Yao, L., Wang, Z., et al. (2020). The clinical implication of dynamic neutrophil to lymphocyte ratio and D-dimer in COVID-19: A retrospective study in Suzhou China. Thrombosis Research 192, 3–8. doi:10.1016/j.thromres.2020.05.006.

Gao, Y., Li, T., Han, M., Li, X., Wu, D., Xu, Y., et al. (2020). Diagnostic utility of clinical laboratory data determinations for patients with the severe COVID-19. Journal of Medical Virology 92, 791–796. doi:https://doi.org/10.1002/jmv.25770.

García de Guadiana-Romualdo, L., Morell-García, D., Rodríguez-Fraga, O., Morales-Indiano, C., María Lourdes Padilla Jiménez, A., Gutiérrez Revilla, J. I., et al. (2021). Cardiac troponin and COVID-19 severity: Results from BIOCOVID study. Eur J Clin Invest, e13532. doi:10.1111/eci.13532.

Gattinoni, L., Chiumello, D., Caironi, P., Busana, M., Romitti, F., Brazzi, L., et al. (2020). COVID-19 pneumonia: different respiratory treatments for different phenotypes? Intensive Care Med 46, 1099–1102. doi:10.1007/s00134-020-06033-2.

Ghahramani, S., Tabrizi, R., Lankarani, K. B., Kashani, S. M. A., Rezaei, S., Zeidi, N., et al. (2020). Laboratory features of severe vs. non-severe COVID-19 patients in Asian populations: a systematic review and meta-analysis. European Journal of Medical Research 25, 30. doi:10.1186/s40001-020-00432-3.

Gómez-Mesa, J. E., Galindo-Coral, S., Montes, M. C., and Muñoz Martin, A. J. (2021). Thrombosis and Coagulopathy in COVID-19. Curr Probl Cardiol 46, 100742. doi:10.1016/j.cpcardiol.2020.100742.

Gong, J., Ou, J., Qiu, X., Jie, Y., Chen, Y., Yuan, L., et al. (2020). A Tool for Early Prediction of Severe Coronavirus Disease 2019 (COVID-19): A Multicenter Study Using the Risk Nomogram in Wuhan and Guangdong, China. Clin Infect Dis 71, 833–840. doi:10.1093/cid/ciaa443.

Grassi, R., Fusco, R., Belfiore, M. P., Montanelli, A., Patelli, G., Urraro, F., et al. (2020). Coronavirus disease 2019 (COVID-19) in Italy: features on chest computed tomography using a structured report system. Sci Rep 10, 17236. doi:10.1038/s41598-020-73788-5.

Grodecki, K., Lin, A., Razipour, A., Cadet, S., McElhinney, P. A., Chan, C., et al. (2021). Epicardial adipose tissue is associated with extent of pneumonia and adverse outcomes in patients with COVID-19. Metabolism 115, 154436. doi:10.1016/j.metabol.2020.154436.

Hastie, C. E., Mackay, D. F., Ho, F., Celis-Morales, C. A., Katikireddi, S. V., Niedzwiedz, C. L., et al. (2020). Vitamin D concentrations and COVID-19 infection in UK Biobank. Diabetes Metab Syndr 14, 561–565. doi:10.1016/j.dsx.2020.04.050.

He, X., Wang, L., Wang, H., Xie, Y., Yu, Y., Sun, J., et al. (2020). Factors associated with acute cardiac injury and their effects on mortality in patients with COVID-19. Scientific Reports 10, 20452. doi:10.1038/s41598-020-77172-1.

Henry, B. M., Benoit, S. W., de Oliveira, M. H. S., Hsieh, W. C., Benoit, J., Ballout, R. A., et al. (2020). Laboratory abnormalities in children with mild and severe coronavirus disease 2019 (COVID-19): A pooled analysis and review. Clinical Biochemistry 81, 1–8. doi:10.1016/j.clinbiochem.2020.05.012.

Hu, X., Hu, C., Yang, Y., Chen, J., Zhong, P., Wen, Y., et al. (2020). Clinical characteristics and risk factors for severity of COVID-19 outside Wuhan: a double-center retrospective cohort study of 213 cases in Hunan, China. Ther Adv Respir Dis 14, 1753466620963035. doi:10.1177/1753466620963035.

Huang, C., Wang, Y., Li, X., Ren, L., Zhao, J., Hu, Y., et al. (2020a). Clinical features of patients infected with 2019 novel coronavirus in Wuhan, China. The Lancet 395, 497–506. doi:10.1016/S0140-6736(20)30183-5.

Huang, W., Li, C., Wang, Z., Wang, H., Zhou, N., Jiang, J., et al. (2020b). Decreased serum albumin level indicates poor prognosis of COVID-19 patients: hepatic injury analysis from 2,623 hospitalized cases. Sci. China Life Sci. 63, 1678–1687. doi:10.1007/s11427-020-1733-4.

Iacobellis, G., Malavazos, A. E., and Ferreira, T. (2020a). COVID-19 Rise in Younger Adults with Obesity: Visceral Adiposity Can Predict the Risk. Obesity 28, 1795–1795. doi:https://doi.org/10.1002/oby.22951.

Iacobellis, G., Secchi, F., Capitanio, G., Basilico, S., Schiaffino, S., Boveri, S., et al. (2020b). Epicardial Fat Inflammation in Severe COVID-19. Obesity 28, 2260–2262. doi:https://doi.org/10.1002/oby.23019.

Iavarone, M., D'Ambrosio, R., Soria, A., Triolo, M., Pugliese, N., Del Poggio, P., et al. (2020). High rates of 30-day mortality in patients with cirrhosis and COVID-19. J Hepatol 73, 1063–1071. doi:10.1016/j.jhep.2020.06.001.

ICNARC – Intensive Care National Audit & Research Centre Available at: https://www.icnarc.org/ [Accessed May 5, 2021].

Incerti, D., Rizzo, S., Li, X., Lindsay, L., Yau, V., Keebler, D., et al. (2021). Prognostic model to identify and quantify risk factors for mortality among hospitalised patients with COVID-19 in the USA. BMJ Open 11, e047121. doi:10.1136/bmjopen-2020-047121.

Izcovich, A., Ragusa, M. A., Tortosa, F., Lavena Marzio, M. A., Agnoletti, C., Bengolea, A., et al. (2020). Prognostic factors for severity and mortality in patients infected with COVID-19: A systematic review. PLoS One 15, e0241955. doi:10.1371/journal.pone.0241955.

Jalde, F. C., Beckman, M. O., Svensson, A. M., Bell, M., Sköld, M., Strand, F., et al. (2021). Widespread Parenchymal Abnormalities and Pulmonary Embolism on Contrast-Enhanced CT Predict Disease Severity and Mortality in Hospitalized COVID-19 Patients. Frontiers in Medicine 8, 917. doi:10.3389/fmed.2021.666723.

Kang, Y., Chen, T., Mui, D., Ferrari, V., Jagasia, D., Scherrer-Crosbie, M., et al. (2020). Cardiovascular manifestations and treatment considerations in COVID-19. Heart 106, 1132–1141. doi:10.1136/heartjnl-2020-317056.

Kim, I.-C., and Han, S. (2020). Epicardial adipose tissue: fuel for COVID-19-induced cardiac injury? European Heart Journal 41, 2334–2335. doi:10.1093/eurheartj/ehaa474.

Kim, L., Garg, S., O'Halloran, A., Whitaker, M., Pham, H., Anderson, E. J., et al. (2021). Risk Factors for Intensive Care Unit Admission and In-hospital Mortality Among Hospitalized Adults Identified through the US Coronavirus Disease 2019 (COVID-19)-Associated Hospitalization Surveillance Network (COVID-NET). Clinical Infectious Diseases 72, e206–e214. doi:10.1093/cid/ciaa1012.

Küçükceran, K., Ayrancı, M. K., Girişgin, A. S., Koçak, S., and Dündar, Z. D. (2021). The role of the BUN/albumin ratio in predicting mortality in COVID-19 patients in the emergency department. Am J Emerg Med 48, 33–37. doi:10.1016/j.ajem.2021.03.090.

Kumar, R., Rathi, H., Haq, A., Wimalawansa, S. J., and Sharma, A. (2021). Putative roles of vitamin D in modulating immune response and immunopathology associated with COVID-19. Virus Research 292, 198235. doi:10.1016/j.virusres.2020.198235.

Lagunas-Rangel, F. A. (2020). Neutrophil-to-lymphocyte ratio and lymphocyte-to-C-reactive protein ratio in patients with severe coronavirus disease 2019 (COVID-19): A meta-analysis. Journal of Medical Virology 92, 1733–1734. doi:10.1002/jmv.25819.

Lang, M., Som, A., Mendoza, D. P., Flores, E. J., Reid, N., Carey, D., et al. (2020). Hypoxaemia related to COVID-19: vascular and perfusion abnormalities on dual-energy CT. Lancet Infect Dis 20, 1365–1366. doi:10.1016/S1473-3099(20)30367-4.

Larici, A. R., Cicchetti, G., Marano, R., Merlino, B., Elia, L., Calandriello, L., et al. (2020). Multimodality imaging of COVID-19 pneumonia: from diagnosis to follow-up. A comprehensive review. Eur J Radiol 131, 109217. doi:10.1016/j.ejrad.2020.109217.

Lei, F., Liu, Y.-M., Zhou, F., Qin, J.-J., Zhang, P., Zhu, L., et al. (2020). Longitudinal Association Between Markers of Liver Injury and Mortality in COVID-19 in China. Hepatology 72, 389–398. doi:10.1002/hep.31301.

Lenka, J., Chhabria, M. S., Sharma, N., Tan, B. E.-X., Boppana, L. K. T., Venugopal, S., et al. (2021). Clinical characteristics and outcomes of critically ill patients with COVID-19 in a tertiary community hospital in upstate New York. J Community Hosp Intern Med Perspect 10, 491–500. doi:10.1080/20009666.2020.1811070.

Li, Q., Ding, X., Xia, G., Chen, H.-G., Chen, F., Geng, Z., et al. (2020). Eosinopenia and elevated C-reactive protein facilitate triage of COVID-19 patients in fever clinic: A retrospective case-control study. EClinicalMedicine 23, 100375. doi:10.1016/j.eclinm.2020.100375.

Li, Y., and Xia, L. (2020). Coronavirus Disease 2019 (COVID-19): Role of Chest CT in Diagnosis and Management. AJR Am J Roentgenol 214, 1280–1286. doi:10.2214/AJR.20.22954.

Liang, W., Liang, H., Ou, L., Chen, B., Chen, A., Li, C., et al. (2020). Development and Validation of a Clinical Risk Score to Predict the Occurrence of Critical Illness in Hospitalized Patients With COVID-19. JAMA Intern Med 180, 1081–1089. doi:10.1001/jamainternmed.2020.2033.

Liao, D., Zhou, F., Luo, L., Xu, M., Wang, H., Xia, J., et al. (2020). Haematological characteristics and risk factors in the classification and prognosis evaluation of COVID-19: a retrospective cohort study. Lancet Haematol 7, e671–e678. doi:10.1016/S2352-3026(20)30217-9.

Lippi, G., and Plebani, M. (2020). Laboratory abnormalities in patients with COVID-2019 infection. Clinical Chemistry and Laboratory Medicine (CCLM) 58, 1131–1134. doi:10.1515/cclm-2020-0198.

Liu, J., Liu, Z., Jiang, W., Wang, J., Zhu, M., Song, J., et al. (2021a). Clinical predictors of COVID-19 disease progression and death: Analysis of 214 hospitalised patients from Wuhan, China. Clin Respir J 15, 293–309. doi:10.1111/crj.13296.

Liu, K.-C., Xu, P., Lv, W.-F., Qiu, X.-H., Yao, J.-L., Gu, J.-F., et al. (2020). CT manifestations of coronavirus disease-2019: A retrospective analysis of 73 cases by disease severity. European Journal of Radiology 126, 108941. doi:10.1016/j.ejrad.2020.108941.

Liu, Q., Wang, Y., Zhao, X., Wang, L., Liu, F., Wang, T., et al. (2021b). Diagnostic Performance of a Blood Urea Nitrogen to Creatinine Ratio-based Nomogram for Predicting In-hospital Mortality in COVID-19 Patients. Risk Manag Healthc Policy 14, 117–128. doi:10.2147/RMHP.S278365.

Lopes, D. V., Lazar Neto, F., Marques, L. C., Lima, R. B. O., and Brandão, A. A. G. S. (2021). Methemoglobinemia and hemolytic anemia after COVID-19 infection without identifiable eliciting drug: A case-report. IDCases 23, e01013. doi:10.1016/j.idcr.2020.e01013.

Lu, X., Jiang, L., Chen, T., Wang, Y., Zhang, B., Hong, Y., et al. (2020). Continuously available ratio of SpO2/FiO2 serves as a noninvasive prognostic marker for intensive care patients with COVID-19. Respiratory Research 21, 194. doi:10.1186/s12931-020-01455-4.

Malavazos, A. E., Goldberger, J. J., and Iacobellis, G. (2020). Does epicardial fat contribute to COVID-19 myocardial inflammation? European Heart Journal 41, 2333–2333. doi:10.1093/eurheartj/ehaa471.

Malik, P., Patel, U., Mehta, D., Patel, N., Kelkar, R., Akrmah, M., et al. (2021). Biomarkers and outcomes of COVID-19 hospitalisations: systematic review and meta-analysis. BMJ Evid Based Med 26, 107–108. doi:10.1136/bmjebm-2020-111536.

Mardani, R., Vasmehjani, A. A., Zali, F., Gholami, A., Nasab, S. D. M., Kaghazian, H., et al. (2020). Laboratory Parameters in Detection of COVID-19 Patients with Positive RT-PCR; a Diagnostic Accuracy Study. Archives of Academic Emergency Medicine 8. Available at: https://www.ncbi.nlm.nih.gov/pmc/articles/PMC7130449/ [Accessed May 10, 2021].

McGurnaghan, S. J., Weir, A., Bishop, J., Kennedy, S., Blackbourn, L. A. K., McAllister, D. A., et al. (2021). Risks of and risk factors for COVID-19 disease in people with diabetes: a cohort study of the total population of Scotland. Lancet Diabetes Endocrinol 9, 82–93. doi:10.1016/S2213-8587(20)30405-8.

Medeiros, A. K., Barbisan, C. C., Cruz, I. R., de Araújo, E. M., Libânio, B. B., Albuquerque, K. S., et al. (2020). Higher frequency of hepatic steatosis at CT among COVID-19-positive patients. Abdom Radiol (NY) 45, 2748–2754. doi:10.1007/s00261-020-02648-7.

Mudatsir, M., Fajar, J. K., Wulandari, L., Soegiarto, G., Ilmawan, M., Purnamasari, Y., et al. (2020). Predictors of COVID-19 severity: a systematic review and meta-analysis. F1000Res 9, 1107. doi:10.12688/f1000research.26186.2.

Munshi, R., Hussein, M. H., Toraih, E. A., Elshazli, R. M., Jardak, C., Sultana, N., et al. (2021). Vitamin D insufficiency as a potential culprit in critical COVID-19 patients. Journal of Medical Virology 93, 733–740. doi:https://doi.org/10.1002/jmv.26360.

O'Driscoll, M., Ribeiro Dos Santos, G., Wang, L., Cummings, D. A. T., Azman, A. S., Paireau, J., et al. (2021). Age-specific mortality and immunity patterns of SARS-CoV-2. Nature 590, 140–145. doi:10.1038/s41586-020-2918-0.

Ok, F., Erdogan, O., Durmus, E., Carkci, S., and Canik, A. (2021). Predictive values of blood urea nitrogen/creatinine ratio and other routine blood parameters on disease severity and survival of COVID-19 patients. J Med Virol 93, 786–793. doi:10.1002/jmv.26300.

Ou, M., Zhu, J., Ji, P., Li, H., Zhong, Z., Li, B., et al. (2020). Risk factors of severe cases with COVID-19: a meta-analysis. Epidemiol Infect 148. doi:10.1017/S095026882000179X.

Ouyang, S.-M., Zhu, H.-Q., Xie, Y.-N., Zou, Z.-S., Zuo, H.-M., Rao, Y.-W., et al. (2020). Temporal changes in laboratory markers of survivors and non-survivors of adult inpatients with COVID-19. BMC Infectious Diseases 20, 952. doi:10.1186/s12879-020-05678-0.

Paliogiannis, P., and Zinellu, A. (2020). Bilirubin levels in patients with mild and severe Covid-19: A pooled analysis. Liver Int 40, 1787–1788. doi:10.1111/liv.14477.

Pan, F., Yang, L., Li, Y., Liang, B., Li, L., Ye, T., et al. (2020a). Factors associated with death outcome in patients with severe coronavirus disease-19 (COVID-19): a case-control study. Int. J. Med. Sci. 17, 1281–1292. doi:10.7150/ijms.46614.

Pan, Y., Guan, H., Zhou, S., Wang, Y., Li, Q., Zhu, T., et al. (2020b). Initial CT findings and temporal changes in patients with the novel coronavirus pneumonia (2019-nCoV): a study of 63 patients in Wuhan, China. Eur Radiol 30, 3306–3309. doi:10.1007/s00330-020-06731-x.

Panigada, M., Bottino, N., Tagliabue, P., Grasselli, G., Novembrino, C., Chantarangkul, V., et al. (2020). Hypercoagulability of COVID-19 patients in intensive care unit: A report of thromboelastography findings and other parameters of hemostasis. J Thromb Haemost 18, 1738–1742. doi:10.1111/jth.14850.
Parlak, S., Çıvgın, E., Beşler, M. S., and Kayıpmaz, A. E. (2021). The effect of hepatic steatosis on COVID-19 severity: Chest computed tomography findings. Saudi J Gastroenterol 27, 105–110. doi:10.4103/sjg.sjg_540_20.
Pelayo, J., Lo, K. B., Bhargav, R., Gul, F., Peterson, E., DeJoy Iii, R., et al. (2020). Clinical Characteristics and Outcomes of Community- and Hospital-Acquired Acute Kidney Injury with COVID-19 in a US Inner City Hospital System. Cardiorenal Med 10, 223–231. doi:10.1159/000509182.
Peng, Y., Wan, L., Fan, C., Zhang, P., Wang, X., Sun, J., et al. (2020). Cholesterol Metabolism—Impacts on SARS-CoV-2 Infection Prognosis. Infectious Diseases (except HIV/AIDS) doi:10.1101/2020.04.16.20068528.
Petrilli, C. M., Jones, S. A., Yang, J., Rajagopalan, H., O'Donnell, L., Chernyak, Y., et al. (2020). Factors associated with hospitalization and critical illness among 4,103 patients with Covid-19 disease in New York City. medRxiv, 2020.04.08.20057794. doi:10.1101/2020.04.08.20057794.
Pimentel, M. A. F., Redfern, O. C., Hatch, R., Young, J. D., Tarassenko, L., and Watkinson, P. J. (2020). Trajectories of vital signs in patients with COVID-19. Resuscitation 156, 99–106. doi:10.1016/j.resuscitation.2020.09.002.
Prilutskiy, A., Kritselis, M., Shevtsov, A., Yambayev, I., Vadlamudi, C., Zhao, Q., et al. (2020). SARS-CoV-2 Infection–Associated Hemophagocytic Lymphohistiocytosis. American Journal of Clinical Pathology 154, 466–474. doi:10.1093/ajcp/aqaa124.
Qin, C., Zhou, L., Hu, Z., Zhang, S., Yang, S., Tao, Y., et al. (2020). Dysregulation of Immune Response in Patients With Coronavirus 2019 (COVID-19) in Wuhan, China. Clinical Infectious Diseases 71, 762–768. doi:10.1093/cid/ciaa248.
Qiu, H., Wu, J., Hong, L., Luo, Y., Song, Q., and Chen, D. (2020). Clinical and epidemiological features of 36 children with coronavirus disease 2019 (COVID-19) in Zhejiang, China: an observational cohort study. The Lancet Infectious Diseases 20, 689–696. doi:10.1016/S1473-3099(20)30198-5.
Radujkovic, A., Hippchen, T., Tiwari-Heckler, S., Dreher, S., Boxberger, M., and Merle, U. (2020). Vitamin D Deficiency and Outcome of COVID-19 Patients. Nutrients 12, E2757. doi:10.3390/nu12092757.
Raharusun, P. (2020). Patterns of COVID-19 Mortality and Vitamin D: An Indonesian Study. Available at: https://pesquisa.bvsalud.org/global-literature-on-novel-coronavirus-2019-ncov/resource/en/ppcovidwho-936 [Accessed May 5, 2021].
Ran, J., Song, Y., Zhuang, Z., Han, L., Zhao, S., Cao, P., et al. (2020). Blood pressure control and adverse outcomes of COVID-19 infection in patients with concomitant hypertension in Wuhan, China. Hypertens Res 43, 1267–1276. doi:10.1038/s41440-020-00541-w.
Ranucci, M., Ballotta, A., Di Dedda, U., Bayshnikova, E., Dei Poli, M., Resta, M., et al. (2020). The procoagulant pattern of patients with COVID-19 acute respiratory distress syndrome. J Thromb Haemost 18, 1747–1751. doi:10.1111/jth.14854.
Raoufi, M., Safavi Naini, S. A. A., Azizan, Z., Jafar Zade, F., Shojaeian, F., Ghanbari Boroujeni, M., et al. (2020). Correlation between Chest Computed Tomography Scan Findings and Mortality of COVID-19 Cases; a Cross sectional Study. Arch Acad Emerg Med 8, e57.
Ren, H., Yang, Y., Wang, F., Yan, Y., Shi, X., Dong, K., et al. (2020). Association of the insulin resistance marker TyG index with the severity and mortality of COVID-19. Cardiovascular Diabetology 19, 58. doi:10.1186/s12933-020-01035-2.
Rod, J. E., Oviedo-Trespalacios, O., Cortes-Ramirez, J., Rod, J. E., Oviedo-Trespalacios, O., and Cortes-Ramirez, J. (2020). A brief-review of the risk factors for covid-19 severity. Revista de Saúde Pública 54. doi:10.11606/s1518-8787.2020054002481.
Rodriguez-Morales, A. J., Cardona-Ospina, J. A., Gutiérrez-Ocampo, E., Villamizar-Peña, R., Holguin-Rivera, Y., Escalera-Antezana, J. P., et al. (2020). Clinical, laboratory and imaging features of COVID-19: A systematic review and meta-analysis. Travel Medicine and Infectious Disease 34, 101623. doi:10.1016/j.tmaid.2020.101623.
Rostami, M., and Mansouritorghabeh, H. (2020). D-dimer level in COVID-19 infection: a systematic review. Expert Rev Hematol 13, 1265–1275. doi:10.1080/17474086.2020.1831383.
Saand, A. R., Flores, M., Kewan, T., Alqaisi, S., Alwakeel, M., Griffiths, L., et al. (2021). Does inpatient hyperglycemia predict a worse outcome in COVID-19 intensive care unit patients? Journal of Diabetes 13, 253–260. doi:https://doi.org/10.1111/1753-0407.13137.
Sardu, C., Gambardella, J., Morelli, M. B., Wang, X., Marfella, R., and Santulli, G. (2020). Hypertension, Thrombosis, Kidney Failure, and Diabetes: Is COVID-19 an Endothelial Disease? A Comprehensive Evaluation of Clinical and Basic Evidence. JCM 9, 1417. doi:10.3390/jcm9051417.
Sattar, N., Ho, F. K., Gill, J. MR., Ghouri, N., Gray, S. R., Celis-Morales, C. A., et al. (2020). BMI and future risk for COVID-19 infection and death across sex, age and ethnicity: Preliminary findings from UK biobank. Diabetes & Metabolic Syndrome: Clinical Research & Reviews 14, 1149–1151. doi:10.1016/j.dsx.2020.06.060.
Shao, M., Li, X., Liu, F., Tian, T., Luo, J., and Yang, Y. (2020). Acute kidney injury is associated with severe infection and fatality in patients with COVID-19: A systematic review and meta-analysis of 40 studies and 24,527 patients. Pharmacol Res 161, 105107. doi:10.1016/j.phrs.2020.105107.
Shi, S., Qin, M., Cai, Y., Liu, T., Shen, B., Yang, F., et al. (2020). Characteristics and clinical significance of myocardial injury in patients with severe coronavirus disease 2019. European Heart Journal 41, 2070–2079. doi:10.1093/eurheartj/ehaa408.
Slipczuk, L., Castagna, F., Schonberger, A., Novogrodsky, E., Sekerak, R., Dey, D., et al. (2021). Coronary artery calcification and epicardial adipose tissue as independent predictors of mortality in COVID-19. Int J Cardiovasc Imaging. doi:10.1007/s10554-021-02276-2.
Suleyman, G., Fadel, R. A., Malette, K. M., Hammond, C., Abdulla, H., Entz, A., et al. (2020). Clinical Characteristics and Morbidity Associated With Coronavirus Disease 2019 in a Series of Patients in Metropolitan Detroit. JAMA Netw Open 3. doi:10.1001/jamanetworkopen.2020.12270.
Sun, C., Hong, S., Song, M., Li, H., and Wang, Z. (2021). Predicting COVID-19 disease progression and patient outcomes based on temporal deep learning. BMC Medical Informatics and Decision Making 21, 45. doi:10.1186/s12911-020-01359-9.
Sun, L., Shen, L., Fan, J., Gu, F., Hu, M., An, Y., et al. (2020). Clinical features of patients with coronavirus disease 2019 from a designated hospital in Beijing, China. J Med Virol 92, 2055–2066. doi:10.1002/jmv.25966.

Taneri, P. E., Gómez-Ochoa, S. A., Llanaj, E., Raguindin, P. F., Rojas, L. Z., Roa-Díaz, Z. M., et al. (2020). Anemia and iron metabolism in COVID-19: a systematic review and meta-analysis. Eur J Epidemiol 35, 763–773. doi:10.1007/s10654-020-00678-5.
Terpos, E., Ntanasis-Stathopoulos, I., Elalamy, I., Kastritis, E., Sergentanis, T. N., Politou, M., et al. (2020). Hematological findings and complications of COVID-19. Am J Hematol 95, 834–847. doi:10.1002/ajh.25829.
Tian, W., Jiang, W., Yao, J., Nicholson, C. J., Li, R. H., Sigurslid, H. H., et al. (2020). Predictors of mortality in hospitalized COVID-19 patients: A systematic review and meta-analysis. J Med Virol 92, 1875–1883. doi:10.1002/jmv.26050.
Vahedian-Azimi, A., Ashtari, S., Alishiri, G., Shahriary, A., Saberi, M., Rahimi-Bashar, F., et al. (2021). The Primary Outcomes and Epidemiological and Clinical Features of Coronavirus Disease 2019 (COVID-19) in Iran. Adv Exp Med Biol 1321, 199–210. doi:10.1007/978-3-030-59261-5_17.
Wan, S., Xiang, Y., Fang, W., Zheng, Y., Li, B., Hu, Y., et al. (2020). Clinical features and treatment of COVID-19 patients in northeast Chongqing. Journal of Medical Virology 92, 797–806. doi:https://doi.org/10.1002/jmv.25783.
Wang, D., Hu, B., Hu, C., Zhu, F., Liu, X., Zhang, J., et al. (2020a). Clinical Characteristics of 138 Hospitalized Patients With 2019 Novel Coronavirus–Infected Pneumonia in Wuhan, China. JAMA 323, 1061–1069. doi:10.1001/jama.2020.1585.
Wang, J. M., Liu, W., Chen, X., McRae, M. P., McDevitt, J. T., and Fenyö, D. (2021a). Predictive modeling of morbidity and mortality in COVID-19 hospitalized patients and its clinical implications. medRxiv, 2020.12.02.20235879. doi:10.1101/2020.12.02.20235879.
Wang, K., Wang, X., Du, J., Liu, C., Jiang, Y., Zhang, H., et al. (2021b). Relationship between changes in the course of COVID-19 and ratio of neutrophils-to-lymphocytes and related parameters in patients with severe vs. common disease. Epidemiol Infect 149, e81. doi:10.1017/S0950268821000674.
Wang, L., Zhang, Y., Cheng, Y., Li, C., Wang, Y., Yan, X., et al. (2020b). Serum triglyceride level and hypertension are highly associated with the recovery of COVID-19 patients. Am J Transl Res 12, 6646–6654. Available at: https://www.ncbi.nlm.nih.gov/pmc/articles/PMC7653618/ [Accessed April 29, 2021].
Webb, B. J., Peltan, I. D., Jensen, P., Hoda, D., Hunter, B., Silver, A., et al. (2020). Clinical criteria for COVID-19-associated hyperinflammatory syndrome: a cohort study. Lancet Rheumatol 2, e754–e763. doi:10.1016/S2665-9913(20)30343-X.
Wool, G. D., and Miller, J. L. (2021). The Impact of COVID-19 Disease on Platelets and Coagulation. PAT 88, 15–27. doi:10.1159/000512007.
Wu, C., Chen, X., Cai, Y., Xia, J., Zhou, X., Xu, S., et al. (2020a). Risk Factors Associated With Acute Respiratory Distress Syndrome and Death in Patients With Coronavirus Disease 2019 Pneumonia in Wuhan, China. JAMA Intern Med 180, 934–943. doi:10.1001/jamainternmed.2020.0994.
Wu, G., Zhou, S., Wang, Y., Lv, W., Wang, S., Wang, T., et al. (2020b). A prediction model of outcome of SARS-CoV-2 pneumonia based on laboratory findings. Sci Rep 10, 14042. doi:10.1038/s41598-020-71114-7.
Xie, J., Covassin, N., Fan, Z., Singh, P., Gao, W., Li, G., et al. (2020a). Association Between Hypoxemia and Mortality in Patients With COVID-19. Mayo Clinic Proceedings 95, 1138–1147. doi:10.1016/j.mayocp.2020.04.006.
Xie, Y., Wang, X., Yang, P., and Zhang, S. (2020b). COVID-19 Complicated by Acute Pulmonary Embolism. Radiology: Cardiothoracic Imaging 2, e200067. doi:10.1148/ryct.2020200067.
Xie, Y., You, Q., Wu, C., Cao, S., Qu, G., Yan, X., et al. (2020c). Impact of Cardiovascular Disease on Clinical Characteristics and Outcomes of Coronavirus Disease 2019 (COVID-19). Circ J 84, 1277–1283. doi:10.1253/circj.CJ-20-0348.
Xu, Z., Shi, L., Wang, Y., Zhang, J., Huang, L., Zhang, C., et al. (2020). Pathological findings of COVID-19 associated with acute respiratory distress syndrome. Lancet Respir Med 8, 420–422. doi:10.1016/S2213-2600(20)30076-X.
Yang, X., Yu, Y., Xu, J., Shu, H., Xia, J., Liu, H., et al. (2020). Clinical course and outcomes of critically ill patients with SARS-CoV-2 pneumonia in Wuhan, China: a single-centered, retrospective, observational study. The Lancet Respiratory Medicine 8, 475–481. doi:10.1016/S2213-2600(20)30079-5.
Yildiz, M., Yadigar, S., Yildiz, B. Ş., Aladag, N. B., Keskin, O., Ozer, R. S., et al. (2021). Evaluation of the relationship between COVID-19 pneumonia severity and pulmonary artery diameter measurement. Herz 46, 56–62. doi:10.1007/s00059-020-05014-x.
Youssef, M., H Hussein, M., Attia, A. S., M Elshazli, R., Omar, M., Zora, G., et al. (2020). COVID-19 and liver dysfunction: A systematic review and meta-analysis of retrospective studies. J Med Virol 92, 1825–1833. doi:10.1002/jmv.26055.
Zhang, B., Dong, C., Li, S., Song, X., Wei, W., and Liu, L. (2020a). Triglyceride to High-Density Lipoprotein Cholesterol Ratio is an Important Determinant of Cardiovascular Risk and Poor Prognosis in Coronavirus Disease-19: A Retrospective Case Series Study. DMSO Volume 13, 3925–3936. doi:10.2147/DMSO.S268992.
Zhang, G., Zhang, J., Wang, B., Zhu, X., Wang, Q., and Qiu, S. (2020b). Analysis of clinical characteristics and laboratory findings of 95 cases of 2019 novel coronavirus pneumonia in Wuhan, China: a retrospective analysis. Respir Res 21, 74. doi:10.1186/s12931-020-01338-8.
Zhang, J.-J., Cao, Y.-Y., Tan, G., Dong, X., Wang, B.-C., Lin, J., Yan, Y.-Q., Liu, G.-H., Akdis, M., Akdis, C. A., & Gao, Y.-D. (2021). Clinical, radiological, and laboratory characteristics and risk factors for severity and mortality of 289 hospitalized COVID-19 patients. Allergy, 76(2), 533–550. https://doi.org/10.1111/all.14496
Zhang, J., Dong, X., Cao, Y., Yuan, Y., Yang, Y., Yan, Y., et al. (2020c). Clinical characteristics of 140 patients infected with SARS-CoV-2 in Wuhan, China. Allergy 75, 1730–1741. doi:10.1111/all.14238.
Zhang, N., Yun, R., Liu, L., and Yang, L. (2020d). Association of glycosylated hemoglobin and outcomes in patients with COVID-19 and pre-existing type 2 diabetes: A protocol for systematic review and meta-analysis. Medicine 99, e23392. doi:10.1097/MD.0000000000023392.
Zhao, L. (2020). Obesity Accompanying COVID-19: The Role of Epicardial Fat. Obesity 28, 1367–1367. doi:https://doi.org/10.1002/oby.22867.
Zhao, Y., Yu, C., Ni, W., Shen, H., Qiu, M., and Zhao, Y. (2021). Peripheral blood inflammatory markers in predicting prognosis in patients with COVID-19. Some differences with influenza A. J Clin Lab Anal 35, e23657. doi:10.1002/jcla.23657.
Zheng, Z., Peng, F., Xu, B., Zhao, J., Liu, H., Peng, J., et al. (2020). Risk factors of critical & mortal COVID-19 cases: A systematic literature review and meta-analysis. J Infect 81, e16–e25.

doi:10.1016/j.jinf.2020.04.021.
Zhou, F., Yu, T., Du, R., Fan, G., Liu, Y., Liu, Z., et al. (2020a). Clinical course and risk factors for mortality of adult inpatients with COVID-19 in Wuhan, China: a retrospective cohort study. The Lancet 395, 1054–1062. doi:10.1016/S0140-6736(20)30566-3.
Zhu, N., Zhang, D., Wang, W., Li, X., Yang, B., Song, J., et al. (2020). A Novel Coronavirus from Patients with Pneumonia in China, 2019. N Engl J Med 382, 727–733. doi:10.1056/NEJMoa2001017.
Zinellu, A., Sotgia, S., Fois, A. G., & Mangoni, A. A. (2021). Serum CK-MB, COVID-19 severity and mortality: An updated systematic review and meta-analysis with meta-regression. Advances in Medical Sciences, 66(2), 304–314. https://doi.org/10.1016/j.advms.2021.07.001

5 Supplementary Figure 1

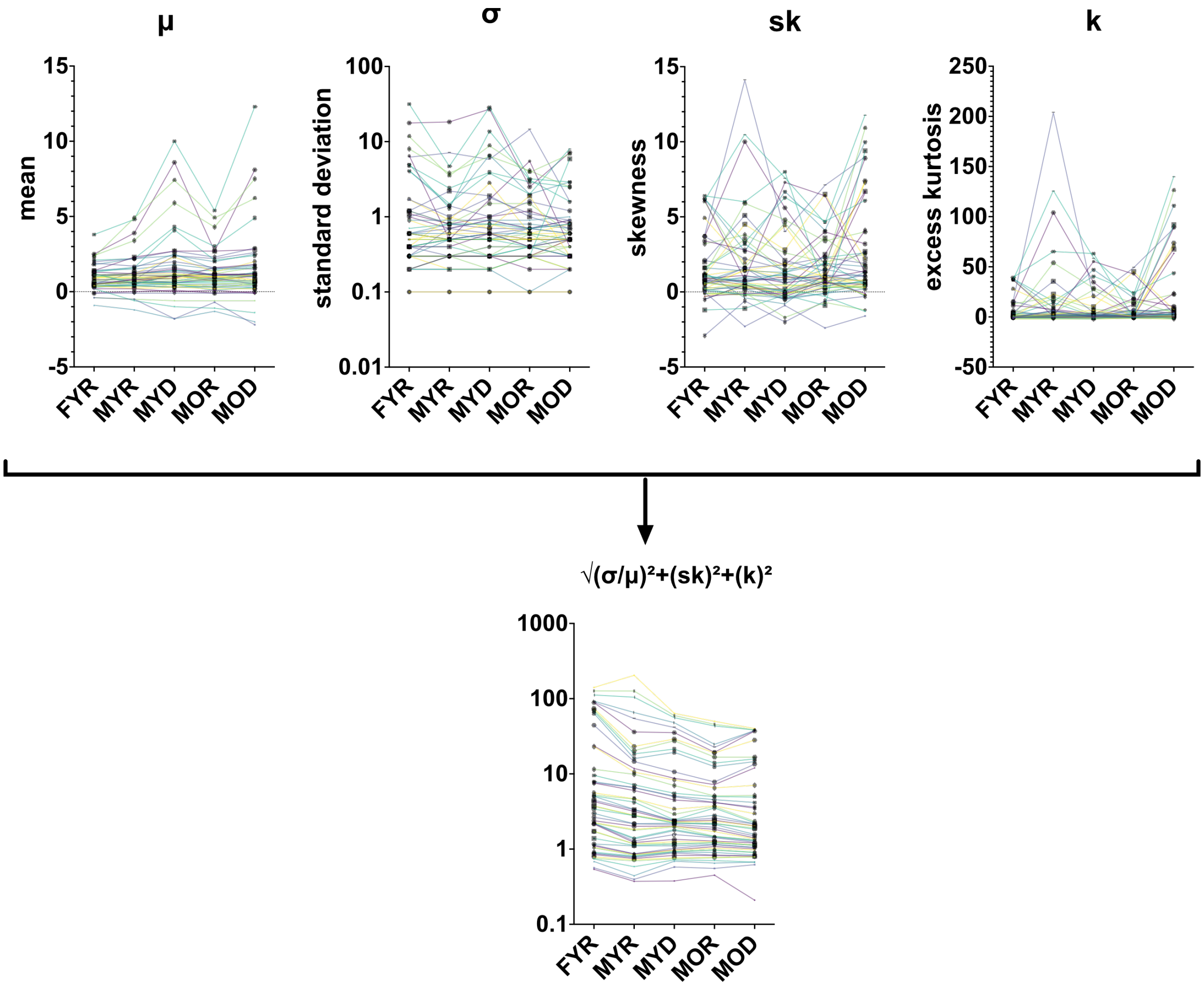

Supplementary Figure 1. Descriptive statistics behavior of each group. The four moments of the distribution of each physiological variable are plotted for each group and summarized by α.

6 Supplementary Figure 2

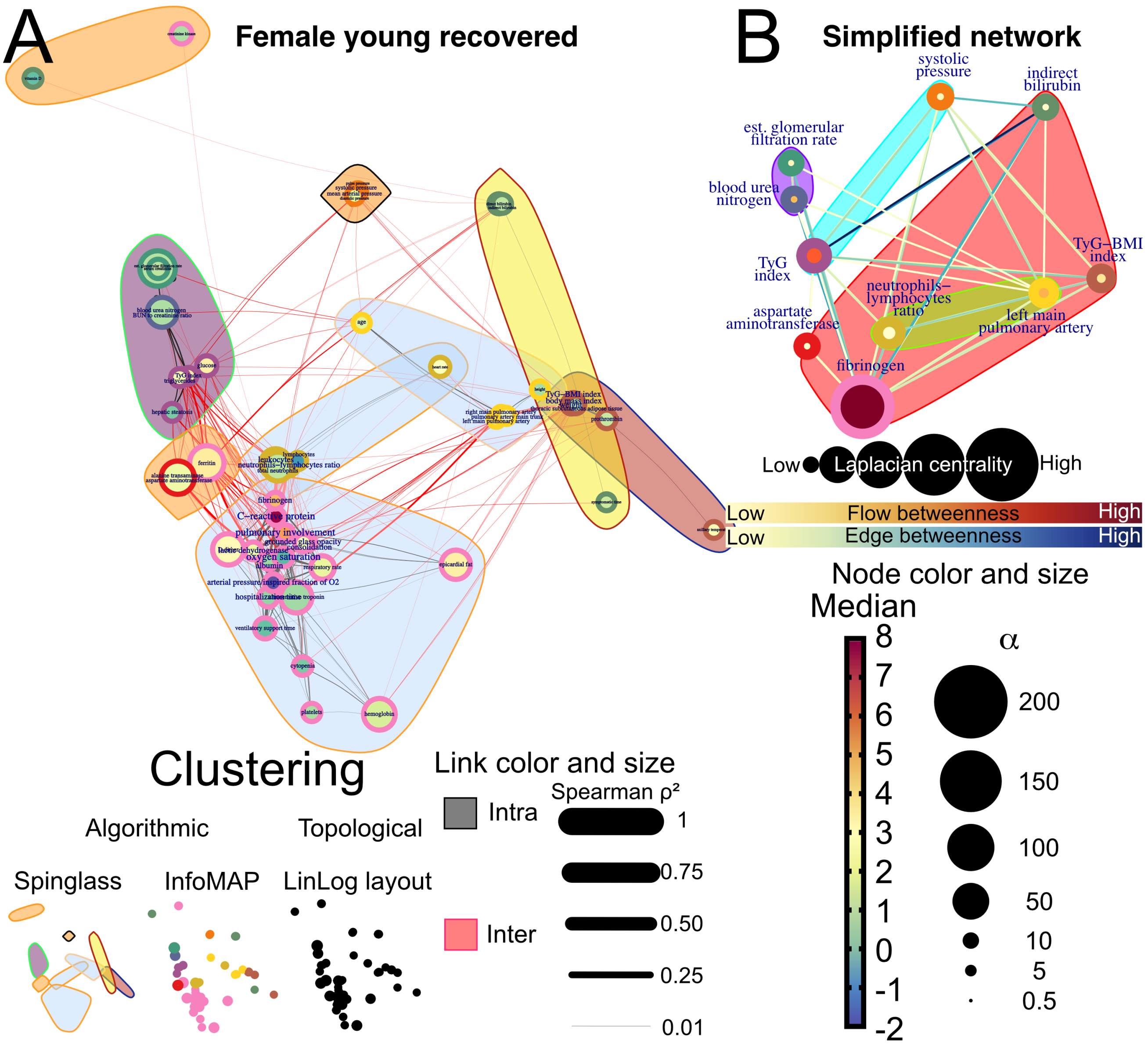

Supplementary Figure 2. Physiological network in female young recovered group. (A) The five study groups' networks were plotted. Links between nodes in the same cluster are shown in black, while shared links are shown in red. Each correlation's strength is indicated by the width of the links. LinLog layout was used to arrange the nodes. The color of the node represents the normalized median value for each study group, and the size represents the deviation from the normal distribution. The color-shaded areas in this network represent spinglass clusters, while the node border represents the nodes that will be collapsed into a single supernode by InfoMAP clustering. (B) depicts a simplified network in which supernodes are labeled with the name of the physiological variable with the greatest influence within the InfoMAP community. Edges were kept, resulting in a network with multiple edges. The color of the edge represents its betweenness. The flow betweenness centrality is represented by node color, and the laplacian centrality is represented by node size.

7 Supplementary Figure 3

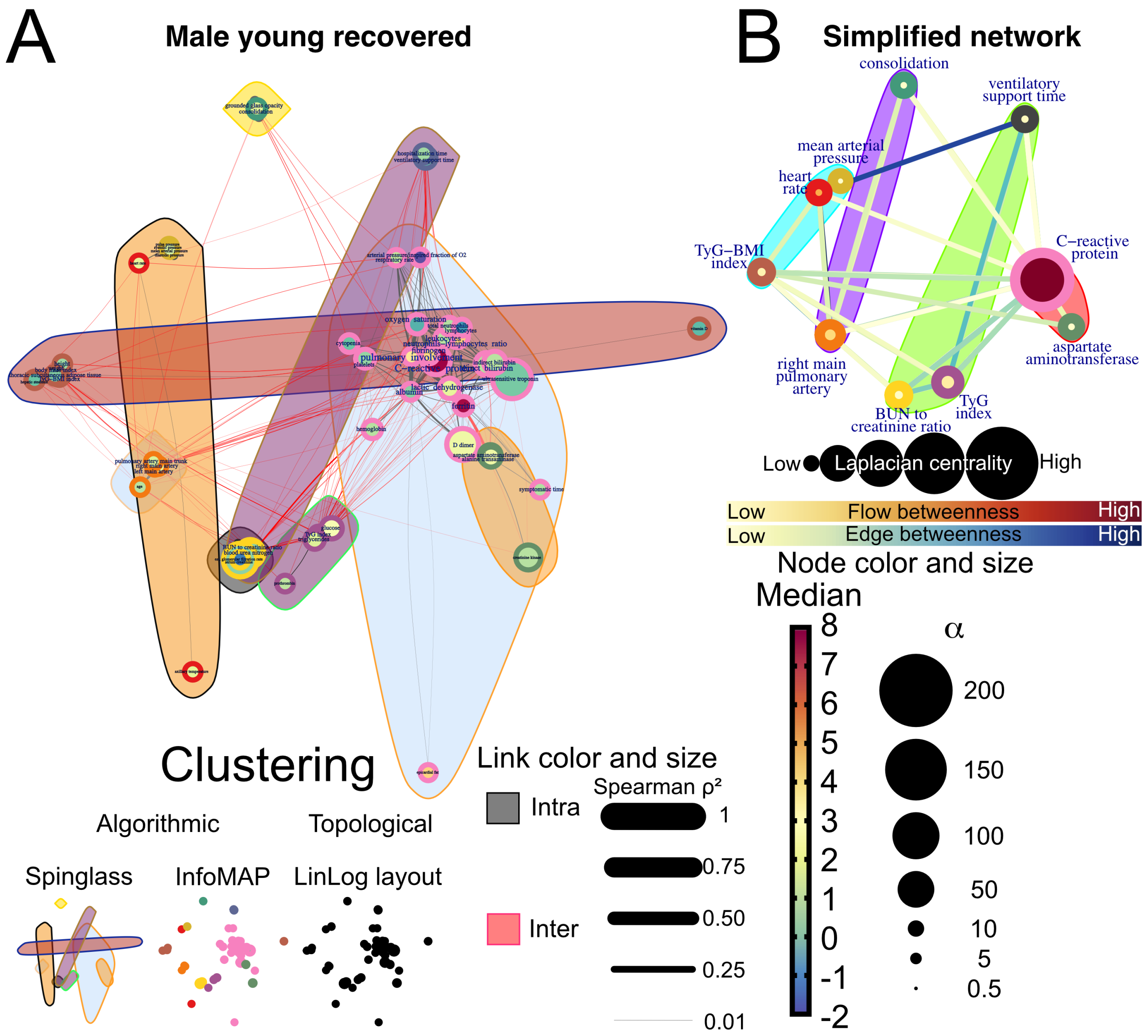

Supplementary Figure 3. Physiological network in male young recovered group. (A) The five study groups' networks were plotted. Links between nodes in the same cluster are shown in black, while shared links are shown in red. Each correlation's strength is indicated by the width of the links. LinLog layout was used to arrange the nodes. The color of the node represents the normalized median value for each study group, and the size represents the deviation from the normal distribution. The color-shaded areas in this network represent spinglass clusters, while the node border represents the nodes that will be collapsed into a single supernode by InfoMAP clustering. (B) depicts a simplified network in which supernodes are labeled with the name of the physiological variable with the greatest influence within the InfoMAP community. Edges were kept, resulting in a network with multiple edges. The color of the edge represents its betweenness. The flow betweenness centrality is represented by node color, and the laplacian centrality is represented by node size.

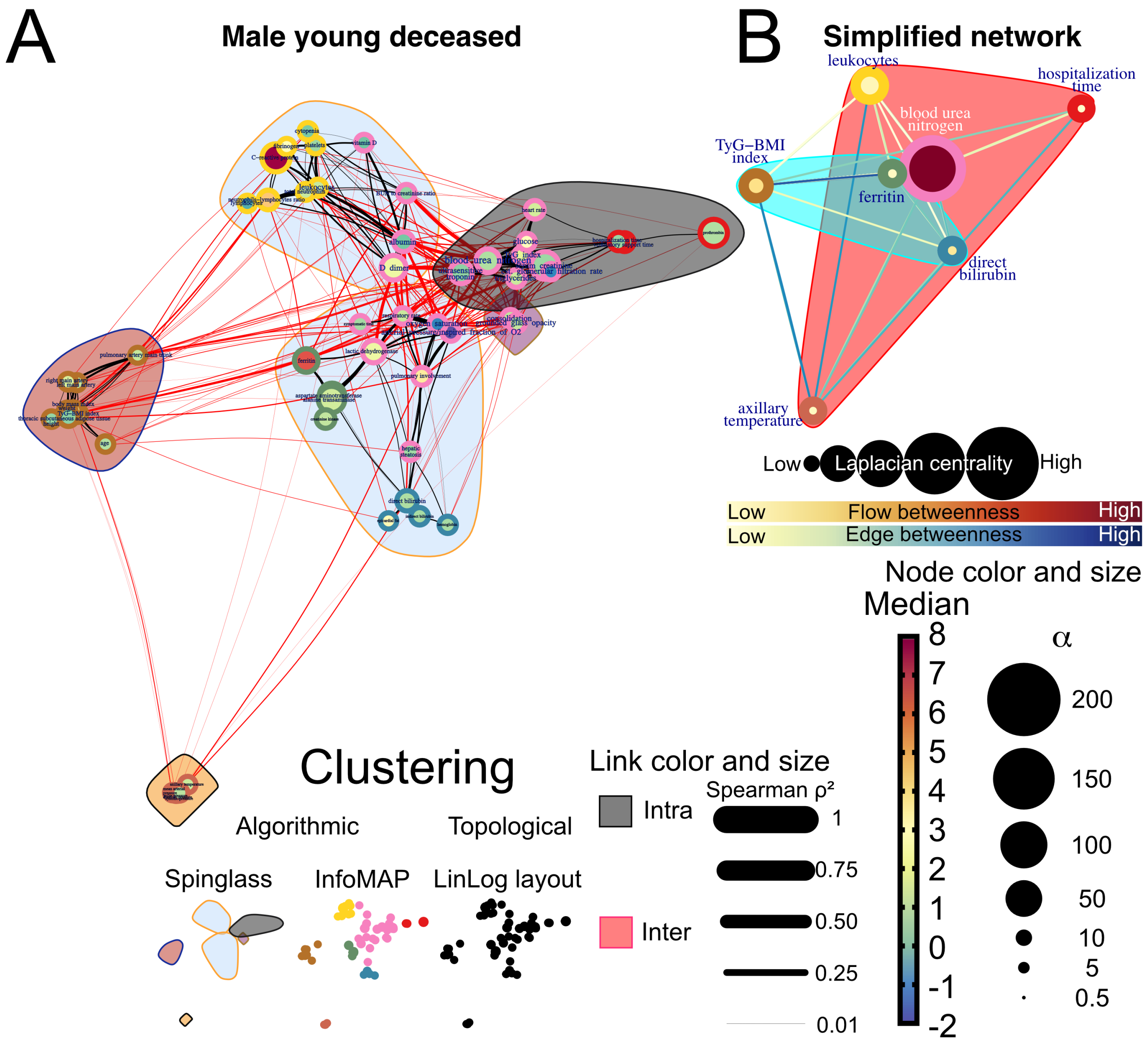

Supplementary Figure 4. Physiological network in male young deceased group. (A) The five study groups' networks were plotted. Links between nodes in the same cluster are shown in black, while shared links are shown in red. Each correlation's strength is indicated by the width of the links. LinLog layout was used to arrange the nodes. The color of the node represents the normalized median value for each study group, and the size represents the deviation from the normal distribution. The color-shaded areas in this network represent spinglass clusters, while the node border represents the nodes that will be collapsed into a single supernode by InfoMAP clustering. (B) depicts a simplified network in which supernodes are labeled with the name of the physiological variable with the greatest influence within the InfoMAP community. Edges were kept, resulting in a network with multiple edges. The color of the edge represents its betweenness. The flow betweenness centrality is represented by node color, and the laplacian centrality is represented by node size.

9 Supplementary Figure 5

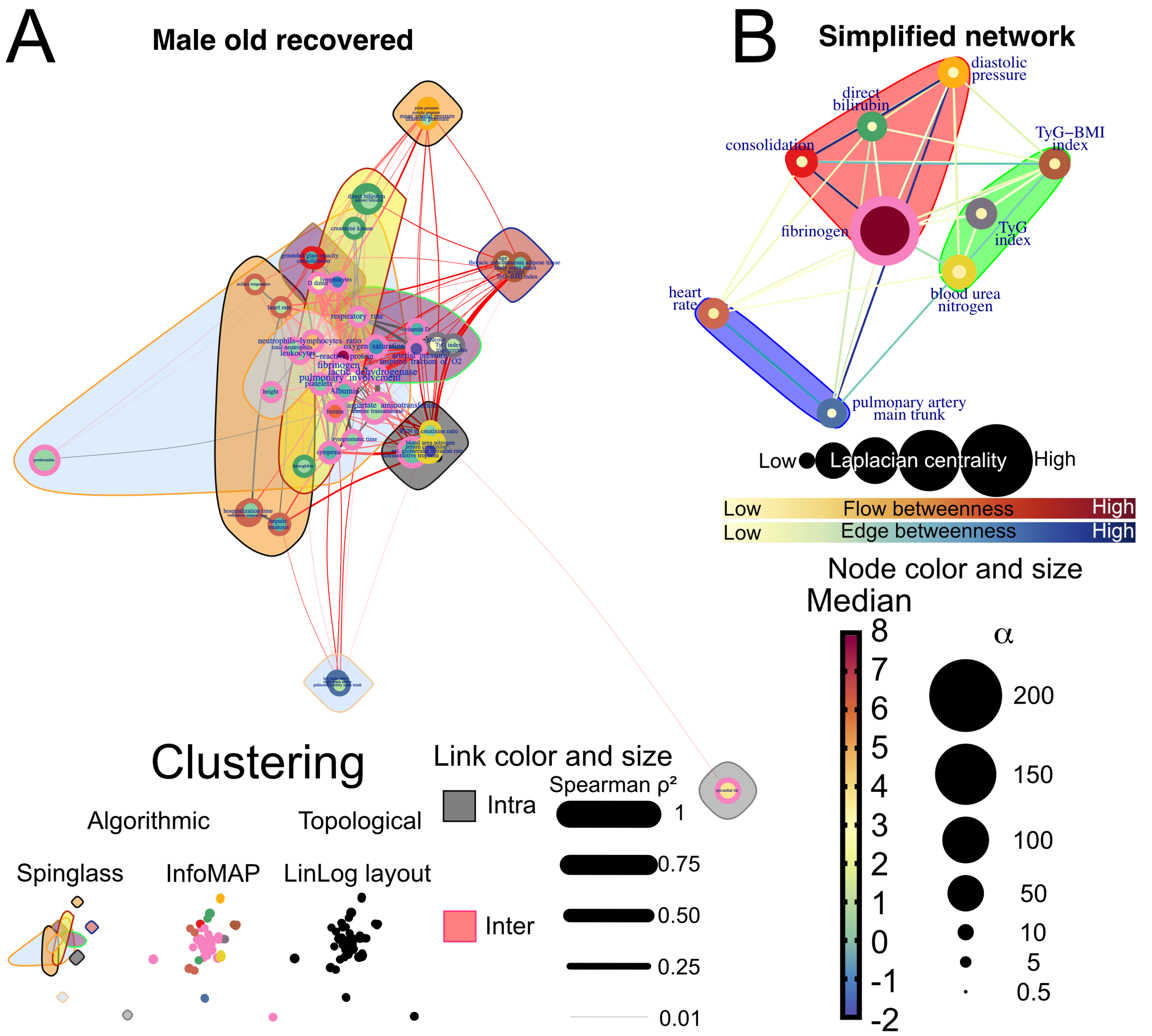

Supplementary Figure 5. Physiological network in male old recovered group. (A) The five study groups' networks were plotted. Links between nodes in the same cluster are shown in black, while shared links are shown in red. Each correlation's strength is indicated by the width of the links. LinLog layout was used to arrange the nodes. The color of the node represents the normalized median value for each study group, and the size represents the deviation from the normal distribution. The color-shaded areas in this network represent spinglass clusters, while the node border represents the nodes that will be collapsed into a single supernode by InfoMAP clustering. (B) depicts a simplified network in which supernodes are labeled with the name of the physiological variable with the greatest influence within the InfoMAP community. Edges were kept, resulting in a network with multiple edges. The color of the edge represents its betweenness. The flow betweenness centrality is represented by node color, and the laplacian centrality is represented by node size.

10 Supplementary Figure 6

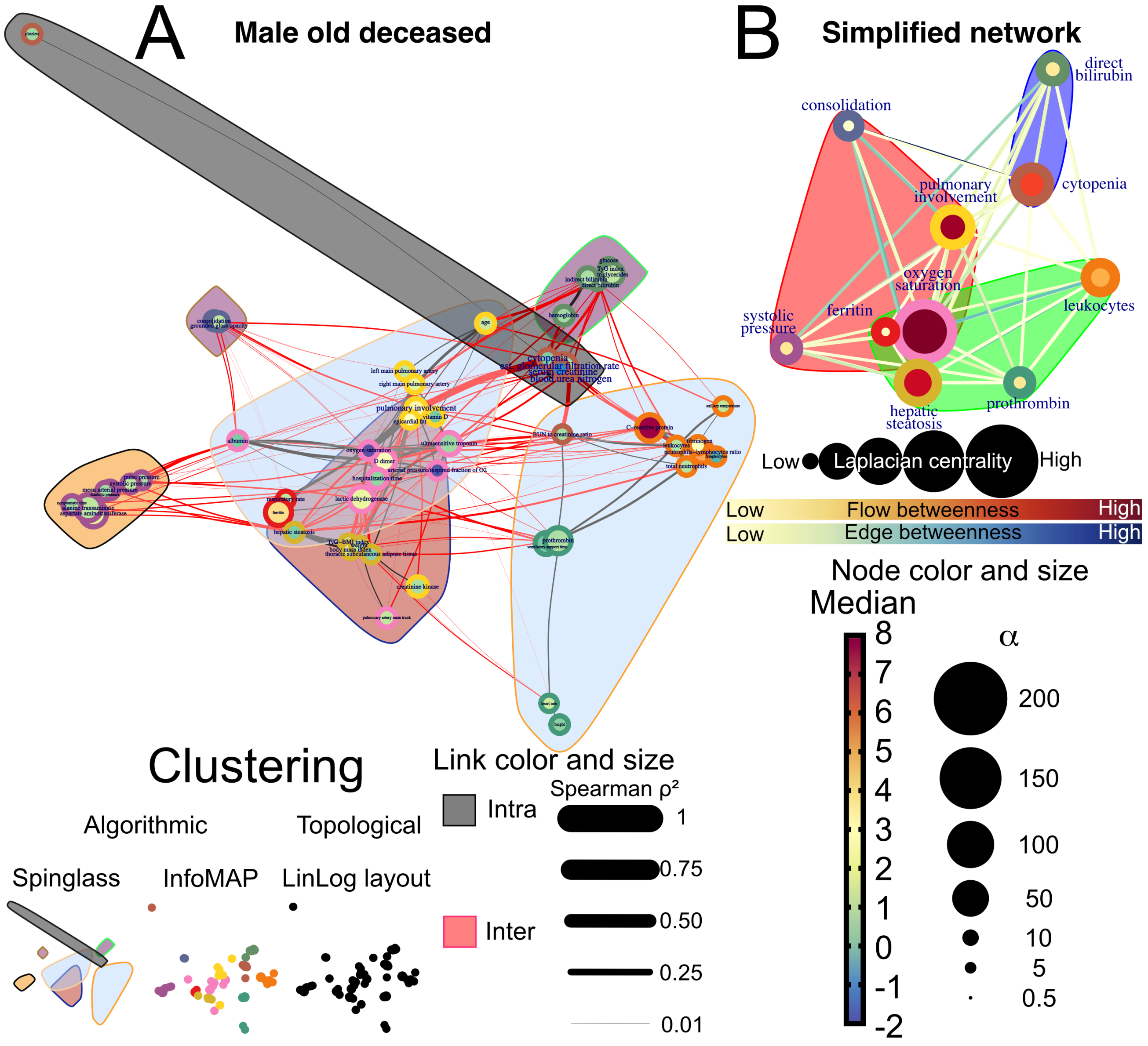

Supplementary Figure 6. Physiological network in male old deceased group. (A) The five study groups' networks were plotted. Links between nodes in the same cluster are shown in black, while shared links are shown in red. Each correlation's strength is indicated by the width of the links. LinLog layout was used to arrange the nodes. The color of the node represents the normalized median value for each study group, and the size represents the deviation from the normal distribution. The color-shaded areas in this network represent spinglass clusters, while the node border represents the nodes that will be collapsed into a single supernode by InfoMAP clustering. (B) depicts a simplified network in which supernodes are labeled with the name of the physiological variable with the greatest influence within the InfoMAP community. Edges were kept, resulting in a network with multiple edges. The color of the edge represents its betweenness. The flow betweenness centrality is represented by node color, and the laplacian centrality is represented by node size.

11 Supplementary Figure 7

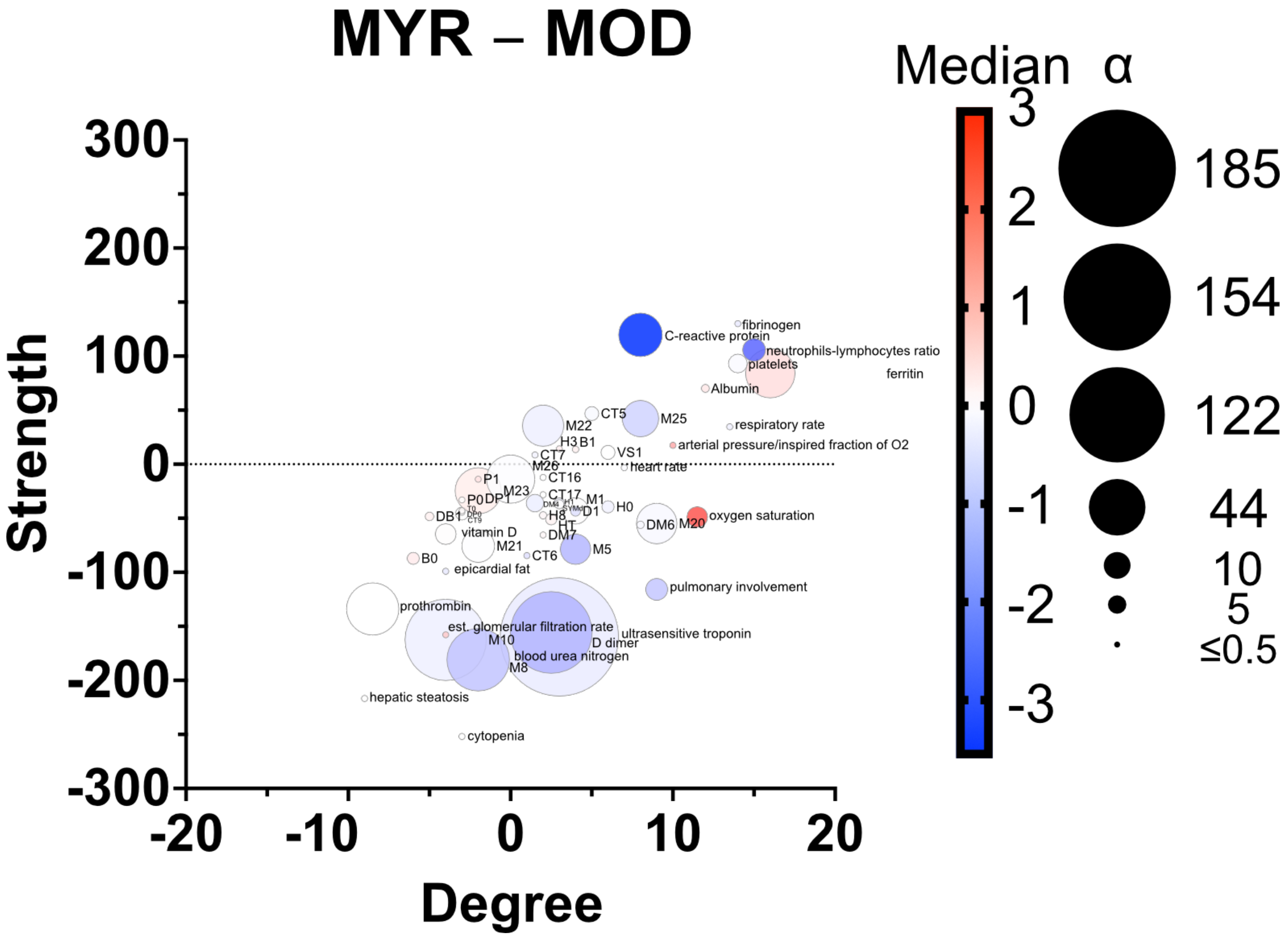

Supplementary Figure 7. Difference between MYR and MOD networks. Physiological variables degree and strength are compared. The gradient of the normalized median value difference is shown in red to blue. The difference between the distributions is indicated by the size of the nodes.

12 Supplementary Figure 8

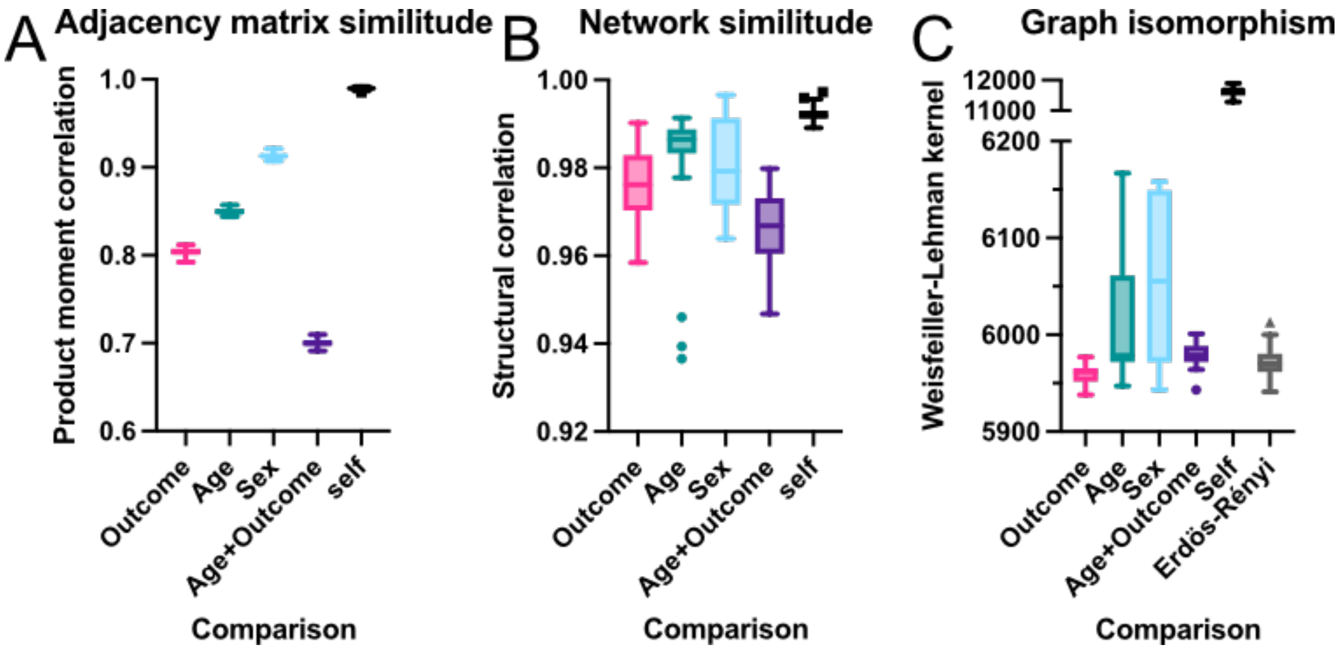

Supplementary Figure 8. Network structure difference. To compare the network structure to the reference, the product-moment correlation (D), structural correlation (F), and Weisfeiler-Lehman isomorphism tests (G) were used. Normalized mutual information (H) was used to quantify cluster similarity, while information variation was used to quantify cluster difference (I). In black we show the comparisson of each network against themselves and in gray against a random Erdös-Rényi network.

13 Supplementary Figure 9

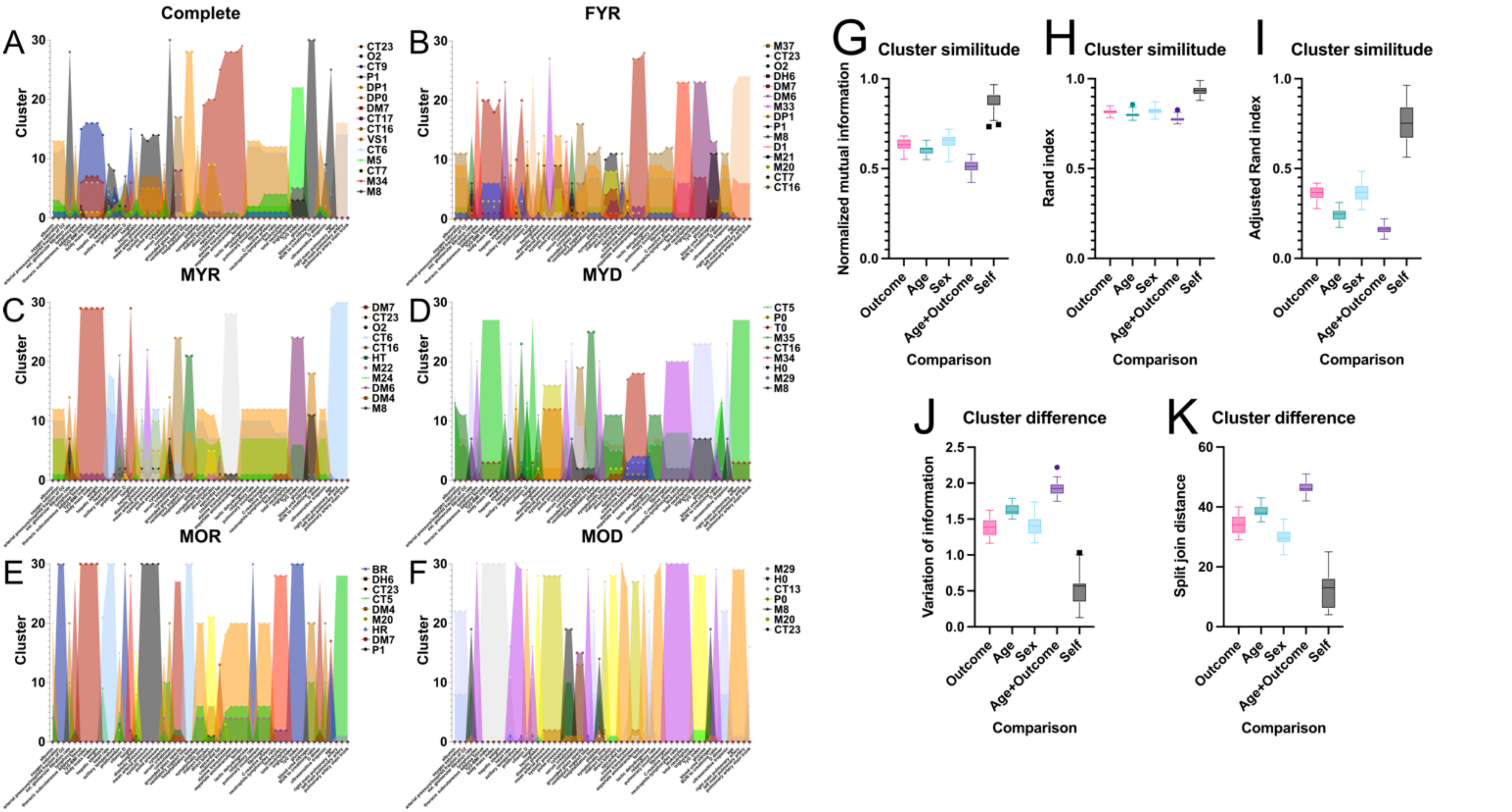

Supplementary Figure 9. Network clusters. Physiological variables were classified into clusters using spinglass algorithm. Clusters were designated after the most relevant variable within each of the 30 network iterations, i.e. the variables with the highest laplacian centrality in each cluster are the core variables. Five clustering comparison methods were employed. In black we show the comparisson of clusters on different iterations of the same network.

14 **Supplementary Figure 10**

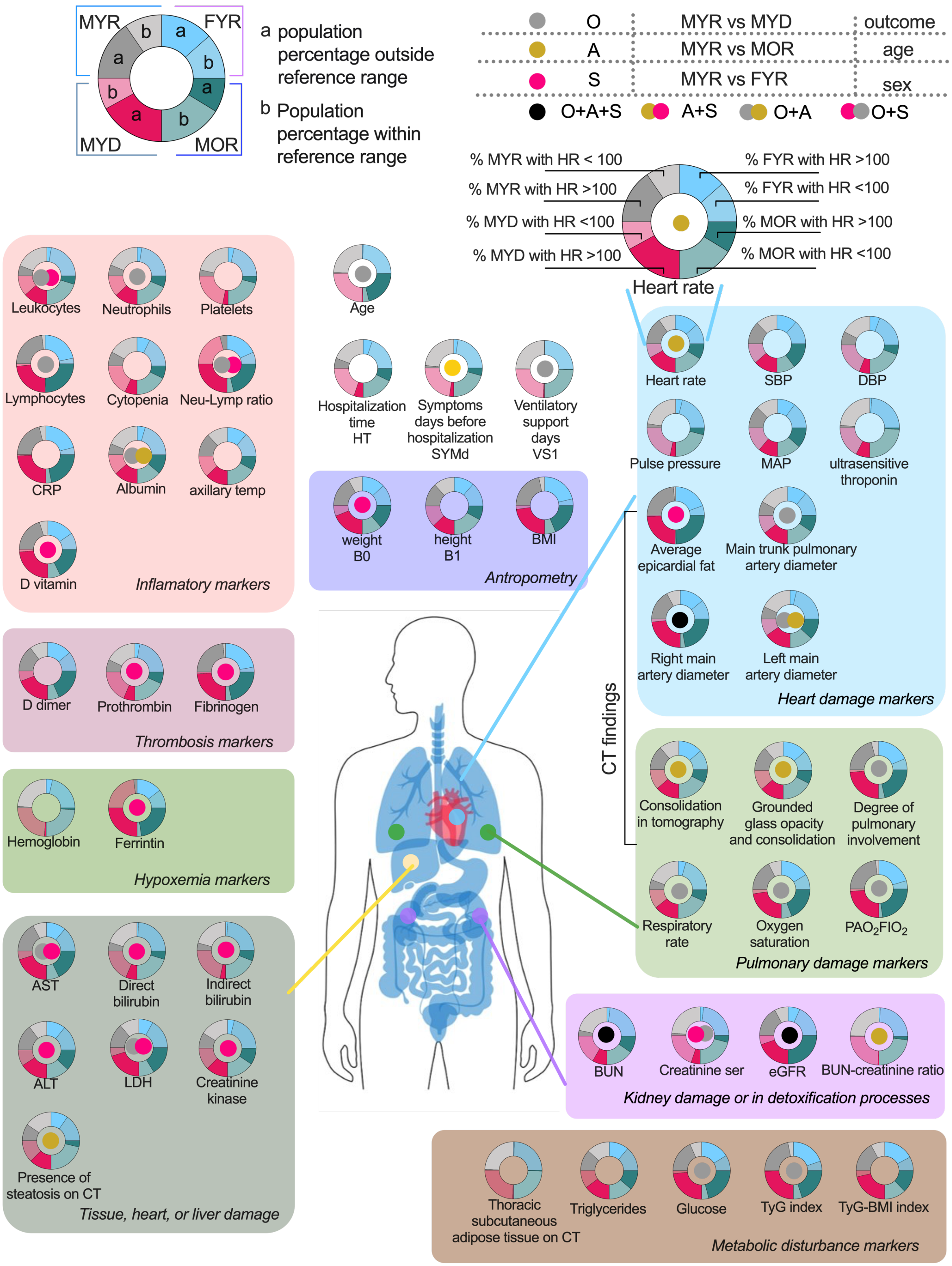

Supplementary Figure 10. Physiological variables differences and pathologic states prevalence among groups. The variables are grouped according to their usefulness as disorders alterations markers. The pie chart graphics show the behavior of the main study groups, the male young recovered (MYR) group is shown in the light blue pair, the female young recovered (FYR) group in the pink pair, the male old recovered (MOR) group in blue and the male young deceased (MYD) group in gray. Each pair is made up of a solid shade (a) and a light shade (b). The solid tone (a) represents the population percentage with values outside the reference limits, while the light tone (b) represents the population percentage with values within the reference limits. The circle in the center indicates the statistically significant differences using Mann-Whitney U. Gray circle indicates a significant difference between the MYR and MYD groups (attributed to the outcome), gold circle indicates the difference between the MYR vs MOR groups (attributed to age), red circle indicates the difference between the MYR vs FYR groups (attributed to sex), while the black circle indicates the presence of statistically significant differences between all groups.
